# Antibiotic Resistance Genes and Bacterial Communities of Farmed Rainbow Trout Fillets (*Oncorhynchus mykiss*)

**DOI:** 10.3389/fmicb.2020.590902

**Published:** 2020-12-03

**Authors:** Nicolas Helsens, Ségolène Calvez, Hervé Prevost, Agnès Bouju-Albert, Aurélien Maillet, Albert Rossero, Dominique Hurtaud-Pessel, Monique Zagorec, Catherine Magras

**Affiliations:** ^1^INRAE, Oniris, SECALIM, Nantes, France; ^2^INRAE, Oniris, BIOEPAR, Nantes, France; ^3^ANSES Laboratoire de Fougères, Unité Analyse des Résidus et Contaminants, Fougères, France

**Keywords:** antibiotic residues, factory processing, raceway, fish fillet, bacterial communities, antibiotic resistance genes

## Abstract

The rise of antibiotic resistance is not only a challenge for human and animal health treatments, but is also posing the risk of spreading among bacterial populations in foodstuffs. Farmed fish-related foodstuffs, the food of animal origin most consumed worldwide, are suspected to be a reservoir of antibiotic resistance genes and resistant bacterial hazards. However, scant research has been devoted to the possible sources of diversity in fresh fillet bacterial ecosystems (farm environment including rivers and practices, and factory environment). In this study bacterial communities and the antibiotic resistance genes of fresh rainbow trout fillet were described using amplicon sequencing of the V3-V4 region of the 16S rRNA gene and high-throughput qPCR assay. The antibiotic residues were quantified using liquid chromatography/mass spectrometry methods. A total of 56 fillets (composed of muscle and skin tissue) from fish raised on two farms on the same river were collected and processed under either factory or laboratory sterile filleting conditions. We observed a core-bacterial community profile on the fresh rainbow trout fillets, but the processing conditions of the fillets has a great influence on their mean bacterial load (3.38 ± 1.01 log CFU/g vs 2.29 ± 0.72 log CFU/g) and on the inter-individual diversity of the bacterial community. The bacterial communities were dominated by Gamma- and Alpha-proteobacteria, Bacteroidetes, Firmicutes, and Actinobacteria. The most prevalent genera were *Pseudomonas*, *Escherichia*-*Shigella*, *Chryseobacterium*, and *Carnobacterium*. Of the 73 antibiotic residues searched, only oxytetracycline residues were detected in 13/56 fillets, all below the European Union maximum residue limit (6.40–40.20 μg/kg). Of the 248 antibiotic resistance genes searched, 11 were found to be present in at least 20% of the fish population (tetracycline resistance genes *tetM* and *tetV*, β-lactam resistance genes *bla*_DHA_ and *bla*_ACC_, macrolide resistance gene *mphA*, vancomycin resistance genes *vanTG* and *vanWG* and multidrug-resistance genes *mdtE*, *mexF*, *vgaB* and *msrA*) at relatively low abundances calculated proportionally to the 16S rRNA gene.

## Introduction

The rise of antibiotic resistance is not only a challenge for human and animal health treatments, but also is posing the risk of spreading among bacterial populations in foodstuffs (pathogen, commensal, spoilage bacteria…) (Woolhouse and Ward, 2013). Recent studies have demonstrated a diverse collection of antibiotic resistance genes maintained in environmental bacterial communities in a variety of ecosystems, including marine sediments (Yang et al., 2013), soils (D’Costa et al., 2006), fish farms (Fernández-Alarcón et al., 2010; Seyfried et al., 2010; Ndi and Barton, 2011; Cesare et al., 2012), processing environments (Chen et al., 2010), and commercialized (supermarket and fish market) fish (Romero et al., 2017). This pool of genetic material, named the resistome (Wright, 2007), which provides the molecular functions for protecting bacteria against most classes of human clinically important antibiotics, also has been recently described in bacterial hazards from fish. Tetracycline resistance profiles and the presence of antibiotic resistance genes have been observed in *Listeria* isolates from catfish fillets (Chen et al., 2010) and *aadA*, *sul1*, and *tet* genes have been found in *Aeromonas* spp. isolated from rainbow trout (gut, liver, skin, mucus, gills, and flesh) (Ndi and Barton, 2011). Fish foodstuffs have therefore been suspected of being antibiotic resistance gene reservoirs but the sources of contamination, such as food production chains and integrated processes (farming practices, environmental contaminations associated with farm environment, and slaughtering process) have rarely been considered.

Among the commonly consumed foodstuffs of animal origin, farmed fish have the lowest carbon footprint and the largest worldwide social and nutritional acceptability. Given the importance of the sector, aquaculture is likely to have a major antibiotic resistance-related public health impact. In 2018, the worldwide consumption of fish-related foodstuffs surpassed 20.5 kg per capita, and global aquaculture production reached as high as 82.1 million tons. Rainbow trout (*Oncorhynchus mykiss*, Walbaum, 1792) accounted for over 848,000 tons of that production, a large part of which was produced in Western Europe (Food and Agriculture Organization of the United Nations, 2020). Farming practices and environments vary greatly around the world. They can strongly influence the resistome, particularly as animal disease treatments, growth promoters and disinfecting or decontaminating agents used in factory environments or on foodstuffs. In Europe, due to the regulatory prohibition of decontamination methods including antimicrobial substances (Regulation EC No 853/2004), the factory processing of food of animal origin does not seem to be a source of antimicrobial residues under any conditions. Furthermore, the control of antimicrobial residues is currently based on a risk assessment and the establishment of maximum residue limits. During this assessment, the effect of residues on human intestinal microbiota is taken into account but the effect of sub-inhibitory concentrations on the development of microbes on foodstuffs is not assessed, with the exception of milk products (Cerniglia and Kotarski, 2005).

A fresh rainbow trout fillet is a complex matrix (skin with mucus, muscles and a thin layer of adipose tissue) with a relatively low bacterial load (3 log CFU/g on average) (Helsens et al., 2020). Nevertheless, this bacterial ecosystem is the result of different sources of contamination. The mucus and the skin-associated bacterial ecosystem can be influenced during the breeding phase, by the breeding practices and by the environment (ponds, river). During factory filleting, the sources of contamination are the digestive tract microbiota and the processing surfaces (González et al., 1999; Chytiri et al., 2004; Galié et al., 2018). Regarding the bacterial communities on the surface of fillets or on fish skin, most studies focused on the presence of human and fish pathogens (Castro-Escarpulli et al., 2003; Chen et al., 2018). The few data obtained on farmed rainbow trout show that skin microbiota is primarily composed of Proteobacteria (Gamma- and Alpha-proteobacteria) and Bacteroidetes (Lowrey et al., 2015; Zhang X. et al., 2018), with *Pseudomonas*, *Chryseobacterium*, and *Shewanella* genera being the most abundant (Zhang X. et al., 2018). The probable diversity of the fresh rainbow trout fillet bacterial ecosystem needs to be better described. This ecosystem could be subject to bacterial fluxes and, consequently, gene transfers, including the transfer of antibiotic resistance genes.

While the presence of antibiotic resistance genes is often reflected by the expression of a resistance phenotype, some genes may be unexpressed, yet still transferable. Such genes can be transferred to other bacteria, through mobile elements as class 1 integrons (Ndi and Barton, 2011; Muziasari et al., 2017) and transposases (Muziasari et al., 2017). It therefore would be interesting to investigate the entire resistome of a matrix or an environment. However, the use of standard microbiological methods alone is not adapted to the investigation of antibiotic resistance gene patterns in large food bacterial communities for several reasons as uncultivable state or the requirement of numerous conditions for detection. To avoid a limitation on the number of genes investigated, high-throughput qPCR assay enables the parallel investigation of a large number of genes and may provide useful data regarding the composition of a resistome associated with a matrix or an environment (Looft et al., 2012; Muziasari et al., 2017).

The objectives of this study were to describe the bacterial communities of fresh rainbow trout fillets using amplicon sequencing of the V3-V4 region of the 16S rRNA gene and to describe the presence of antibiotic resistance genes by high-throughput qPCR, and to quantify antimicrobial residues using liquid chromatography/mass spectrometry (LC-MS/MS) methods. This combined approach allows an overall view of the antibiotic resistance profiles in one example of aquaculture-related foodstuffs.

## Materials and Methods

### Sampling Plan

Our sampling strategy sought to identify the conditions where the bacterial microbiota and the antibiotic resistance profiles of the fillets could be observed. These conditions were the fish farming environment (named the Raceway condition in the study) and the farming and factory environments combined (named the Factory condition in the study). The study also was designed to have the ability to detect a low-frequency event – the presence of antibiotic resistance genes – within a community of bacterial species which may itself be small (Gordon et al., 2007; Muziasari et al., 2017; Helsens et al., 2020). Published data regarding the occurrence of antibiotic resistance in specific pathogenic species and in the bacterial populations of an environment are available. However, the *a priori* occurrence of antibiotic resistance genes within a bacterial community associated with a fresh food matrix is difficult to determine. A compromise therefore had to be made between a sample size needed to detect an event with a 20% prevalence in a study population of 100 individuals or more and the economic and technical context of the study. Finally, a total of 56 fish/fillets (14 per sampling condition) were studied, according to the epidemiological guidelines provided by Cannon and Roe (1982).

**FIGURE 1:**
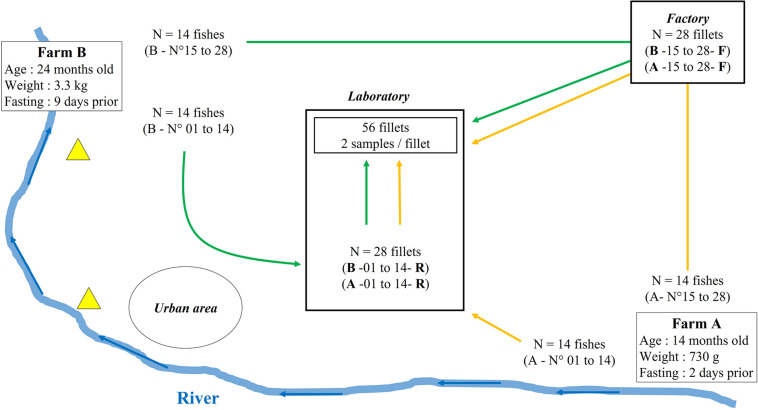
Schematic representation of the sampling plan. Location and zootechnical characteristics of both tested farms are indicated. Yellow (farm A) and green (farm B) arrows indicate fish sampling and filleting either in the factory or aseptically in the laboratory. Yellow triangles represent wastewater treatment plants and blue arrows represent the stream of the river.

Two rainbow trout farms (flow-through systems) located on the same river and processing their fish in the same factory were recruited on a voluntary basis (Figure 1). The first, farm A, is located at the headwaters of the river and produces 150 tons a year. The second, farm B, is located downstream from farm A and produces 250 tons a year. Two wastewater treatment facilities and an urban area were located between farm A and farm B. The fish of each farm were collected one week apart in January 2019. The sampled raceways each contained around 10,000 fish. The zootechnical characteristics of the fish from each farm are described in Figure 1. In parallel, a questionnaire was filled in by the farmers to gather information concerning the conditions in which the fish were bred (bacterial pathologies, antibiotic treatments, vaccinations, water supplying of the raceways, etc…).

On each farm, 28 fish ready to be processed were randomly sampled from a raceway, stunned according to animal welfare regulations (Council Regulation (EC) No 1099/2009) and stored on ice. Half (14 fish) were transformed in the processing factory within three hours following the sampling, where they were subjected to the operator’s processing procedures – automatic slicing, gutting and filleting. The second half (14 fish) were placed in insulated boxes with ice, transported to the laboratory within six hours of handling, and filleted under sterile conditions. Each fillet was given an identification code such as “A11R,” corresponding to the farm (A or B), the chronological order of sampling and filleting of the fish (01–28 for each farm) and the condition influencing the bacterial communities and antibiotic resistance genes (R for Raceway condition, reflecting the raceway environment and sterile filleting conditions in a laboratory, and F for Factory condition, reflecting the raceway and the factory environment with the usual factory processing conditions). From each fillet, two 30 g samples were cut, with flesh 1–1.5 cm thick and a skin surface of about 20–25 cm^2^. One portion was used for antibiotic residue analysis and the second for DNA extraction and analysis (antibiotic resistance gene -qPCR and amplicon sequencing). Each sample was stored at −20°C until analysis.

### Analysis of Antibiotic Residues

The physico-chemical characterization of antibiotic residues was conducted in two steps using liquid chromatography/mass spectrometry (LC-MS/MS) methods: a first screening analysis was implemented for all samples to detect positive samples, and in the case of the presence of an antibiotic, a second analysis was performed to quantify the identified substance with a specific method. For the first screening method, antibiotic residues were extracted and analyzed by liquid chromatography/mass spectrometry (LC-MS/MS) as previously described by Dubreil et al. (2017). Briefly, 2 g of a fillet sample were ground then extracted with 8 mL acetonitrile (Fisher Scientific, France), evaporated, then re-dissolved in 600 μL of 0.2 M ammonium acetate (Merck, Fontenay-sous-Bois, France), and injected into the LC-MS/MS system. The liquid chromatography was performed on a Shimadzu LC-20AD-XR system (Kyoto, Japan) fitted with a Waters Symmetry C18 column (150 × 3.9 mm, 5 μm) (Guyancourt, France) and connected to the Sciex API 5500 mass spectrometer (San Jose, CA, United States).

A panel of 73 antibiotics belonging to different families (including penicillins, cephalosporins, sulfamides, tetracyclines, macrolides, cephalosporins, macrolides, quinolones, phenicols) were searched for using highly sensitive methods (Dubreil et al., 2017). For the present study, a second method was used for the quantification of tetracycline compound residues (*internal reference F/CHIM/SM/PTC/007*) as follows: 2 g of minced sample was extracted with 10 mL Mac Ilvaine / EDTA buffer, and mixed for 10 min. A deproteinization step was then carried out adding 1 mL trichloroacetic acid (Fisher Scientific, Illkirch, France) solution at 1 g/L. The extract was further purified using SPE clean-up with C18 Bond-Elut cartridge (Agilent Technologies, Les Ulis, France). Tetracyclines were finally recovered with 1.2 mL of oxalic acid in methanol (Fisher Scientific, France). The final extract was diluted with 1.8 mL of water before injection into the LC-MS/MS system. These analyses were performed at the European Union Reference Laboratory (EU-RL) for antimicrobial and dye residues in food operating under the French Agency for Food, Environmental and Occupational Health & Safety (ANSES, Fougères, France).

### DNA Extraction

DNA extraction for amplicon sequencing and antibiotic resistance gene detection was performed as previously described (Helsens et al., 2020). Briefly, 30 g of a fillet sample were rinsed with 50 ml phosphate buffer saline (Interchim, Montluçon, France) and 5% Tween 80 (Sigma-Aldrich, MO, United States) in a stomacher bag with a 63-μm porosity filter (BagPage 400 F, Interscience). After rinsing, the liquid phase was filtered through the stomacher bag membrane and centrifuged, and the bacterial pellet was stored at −20°C until DNA extraction. DNA was extracted using the Dneasy^®^ PowerFood^®^ Microbial Kit (Qiagen, Courtaboeuf, France) according to manufacturer’s instructions, with added enzymatic and mechanical cell lysis steps. Afterward, DNA was quantified using a N60 NanoPhotometer^®^ (Implen, München, Germany) and then stored at −20°C until use. As amplicon sequencing needs negative controls to exclude DNA contamination during extraction, mock extractions (fish fillet samples omitted) were also performed.

### Enumeration of Bacterial Counts by q-PCR Targeting the *tuf* Gene

To quantify the bacterial load of each sample, a bacterial enumeration was performed using a quantitative PCR targeting the *tuf* gene, as described by Tanaka et al. (2010). A quantitative *tuf* PCR kit for bacteria (Takara Bio, United States), using SYBR^®^ green technology, was used. Quantitative PCR was performed with 5 μL of template DNA in a total volume of 25 μL. The reaction mix was composed according to the manufacturer’s instructions. The amplification program included an initial denaturing step of 30 s at 95°C followed by 35 cycles of 5 s at 95°C and 30 s at 60°C. A negative control was included in each run. The amplification was achieved on a CFX Connect^TM^ Real-Time PCR Detection System (Bio-Rad, Marnes-la-Coquette, France). All amplification reactions were run in triplicate. A positive *tuf* gene standard stock solution provided in the kit was used to generate a linear standard curve by plotting the C_T_ values versus 5 × 10^5^ to 5 × 10^2^ copies/reaction. This standard curve was used to calculate copies/reaction then copies/g (and CFU/g) (see Supplementary Figure 1 for amplification data).

### Antibiotic Resistance Gene qPCR Array

In France, the antibiotics authorized for the treatments of fish diseases are florfenicol, flumequine, oxolinic acid, oxytetracycline (OTC), sulphadiazine and trimethoprim. We primarily aimed to assess the presence of genes conferring resistances to these antibiotics, but we also included resistance genes for critically important antibiotics and human therapeutic antibiotics to avoid being limited by our choice of genes targeted. A set of 248 primer pairs (Supplementary Table 1) was selected from a list of primers published by Muziasari et al. (2017) which were designed to target sequence diversity within a gene and had been validated in previous studies (Pitkanen et al., 2011; Tamminen et al., 2011; Looft et al., 2012; Zhu et al., 2013; Muziasari et al., 2014). To this list were added five primer pairs targeting colistin resistance genes *mcr-1* (Liu et al., 2016), *mcr-2* (Xavier et al., 2016), *mcr-3* (Yin et al., 2017), *mcr-4* (Carattoli et al., 2017) and *mcr-5* (Borowiak et al., 2017). The primer set used in this study contained 190 primer pairs specific to genes encoding resistance to the nine main antibiotic classes (aminoglycosides, beta-lactams, colistin, fluoroquinolones, macrolides, phenicols, tetracycline, trimethoprim and vancomycin) and 58 targeting genes encoding multidrug-resistance and efflux pumps (*N* = 35), resistance to antiseptics (*N* = 7), antibacterial peptide resistance genes (*N* = 10) and housekeeping genes (*N* = 6). A negative control (no DNA) was added to each qPCR run.

The qPCR amplification was performed by the “Human and Environmental Genomics” Platform (Rennes, France), using the Takara SmartChip Real-time PCR system (Takara, United States) which runs a high-throughput, nanoliter-scale real-time PCR. The 5184-well plates with a reaction volume of 100 nl were filled with the SmartChip MultiSample NanoDispenser (Takara, United States). The SmartChip MyDesign Kit (Takara, United States) was used and the PCR cycling conditions were as follows: denaturation at 95°C for 5 min followed by 42 cycles of a cycle including denaturation at 95°C for 10 s, annealing at 60°C for 30 s and elongation at 72°C for 30 s. A final round of denaturation-annealing was performed. The specificity of amplification was assessed through the analysis of the melting curve of each PCR product. The detection limit of amplification was set at a threshold cycle (C_T_) of 27 (Zhu et al., 2013; Muziasari et al., 2017). The relative abundance of each detected genes was calculated proportionally to the 16S rRNA gene in each sample using the 2^–ΔCT^ method, in which ΔC_T_ = (C_T_ detected gene – C_T_ 16S rRNA gene).

### 16S rRNA Gene Sequencing and Data Processing

To detect the bacterial communities, the V3-V4 amplicons of the 16S rRNA gene were sequenced. Control quality, PCR amplification, and sequencing were performed by Genoscreen (Lille, France) according to the Metabiote^®^v2.0 protocol. The 16S rRNA gene V3-V4 region was sequenced on an Illumina Miseq sequencer (Illumina, San Diego, CA, United States) using the MiSeq Reagent Kit v3 (2 × 250 bp paired-end reads). Demultiplexing, merging of the reads and trimming of the barcodes and primers were performed by the sequencing provider. Data were imported into the FROGS (Find Rapidly OTUs with Galaxy Solution) pipeline (Escudié et al., 2018). Denoising and clustering of the reads into Operational Taxonomic Units (OTUs) were performed using the SWARM method (Mahé et al., 2014). Sequences with an abundance <0.005% to the total number of sequences in the entire dataset were removed. Taxonomy assignments were performed using NCBI blastn+ against the Silva 16S rRNA gene database (SSURef_132_SILVA) (Quast et al., 2013). Clusters affiliated to the matrix (rainbow trout) DNA and OTUs with a BLAST coverage or identity below 97% were removed.

### Statistical Analysis

The statistical analyses of the bacterial load of the fillets according to sampling conditions (farm A or B, Raceway or Factory) were realized using Student’s *t*-tests on XLSTAT (v. 2019.2.2). A significant difference was expressed by a *p*-value below the 5% confidence interval.

The statistical analysis of the treated amplicon sequencing data, namely data analysis of the affiliated OTUs, α-diversity indices (number of observed OTUs, Chao1, Shannon, and Inverse-Simpson indices) and β-diversity indices (Jaccard and Bray-Curtis), were calculated in the R environment using the Phyloseq package (v. 1.20.0) (McMurdie and Holmes, 2013) including data visualization through multidimensional scaling on the FROGSSTAT tool. Permutational analysis of variance (PERMANOVA) statistical analysis were calculated using the Phyloseq package (v. 1.30.0) on R software (v. 3.6.2), in order to evaluate the significance of bacterial community differences. UpSet plot was used to assess shared OTUs depending on the farm localization and filleting condition (Lex et al., 2014). This plot was generated using UpSetR package (v. 1.4.0) (Conway et al., 2017) on R software (v. 3.6.2).

ANOVA with multiples comparisons were used to assessed farming and processing effects on genera relative abundances. *P*-values were adjusted using the Benjamini-Hochberg correction (Benjamini and Hochberg, 1995). Tukey test was used as an ANOVA post-hoc test.

## Results

### Antibiotic Residues Assay

Among the 73 antibiotic residues which could be quantified, none were detected in the rainbow trout fillets from farm A. Only OTC residues were detected in 13 out of the 28 fillets from farm B (Table 1). In the 13 OTC positive fillets, residues were detected at concentrations ranging from 5.84 to 40.20 μg/kg, thus below 100 μg/kg which is the maximum residue limit set for muscle-related foodstuffs in the European Union (Commission Regulation (EU) No 37/2010). According to the questionnaire we submitted, the batch from farm B underwent an OTC treatment which ended 27 weeks (192 days) before the sampling day.

**TABLE 1:** Detection and quantification of antibiotic residues in the rainbow-trout fillets, depending on farm and filleting conditions.

**Fillets**			**Antibiotic residues**	
	
**Farm**	**Conditions of filleting**	**Sample number**	**Detection**	**Concentration (μg/kg)**
A	R^a^	01 to 14	–^c^	ND^d^
	F^b^	15 to 28	–	ND
B	R	01, 03, 05, 06, 07, 10, 11, 12, 13	–	ND
		09	Oxytetracycline	5.84
		04		6.40
		02		30.10
		14		33.60
		08		40.20
	F	17, 20, 21, 24, 26, 27	–	ND
		15	Oxytetracycline	7.02
		19		7.37
		18		9.00
		22		9.85
		25		12.60
		16		22.60
		23		31.20
		28		33.90

*^a^Raceway. ^b^Factory. ^c^Not detected. ^d^Not determined as no antibiotic residue was detected.*

### Analysis of the Fillet-Related Bacterial Microbiota

#### Bacterial Load of the Fillets

The mean bacterial contamination of the samples, depending on farm (A/B) and filleting conditions (F/R), are shown in Table 2. For 3 samples (B06R, B12R and B22F), the amplification was not satisfactory and the data were not considered for calculation of the mean bacterial quantification. The mean of AF fillets bacterial counts was significantly higher than the mean counts of AR fillets (respectively, 3.65 ± 1.20 log CFU/g and 1.7 ± 0.35 log CFU/g, *p*-value <0.0001). Conversely for farm B fillets, no significant difference was observed in the bacterial counts between the filleting conditions (2.97 ± 0.30 log CFU/g vs 3.09 ± 0.70 log CFU/g respectively). Comparison by farm source of fillets showed that there was a significant difference, with a higher bacterial load of fillets from farm B than those from farm A (*p*-value <0.0001), as BR fillets had higher bacterial load than AR fillets (2.97 ± 0.3 log CFU/g vs 1.70 ± 0.35 log CFU/g). When combining the fillets from both farms, F fillets had higher bacterial load than R fillets (3.38 ± 1.01 log CFU/g vs 2.29 ± 0.72 log CFU/g, *p*-value <0.0001).

**TABLE 2:** Mean bacterial load of fillets quantified using the *tuf* gene-targeted qPCR, in log CFU/g.

**Farm**	**R fillets**			**F fillets**		
	***N***	**Mean log CFU/g**	**SD**	***N***	**Mean log CFU/g**	**SD**
A	14	1.70^a,b^	0.35	14	3.65^a^	1.20
B	12	2.97^b^	0.30	13	3.09	0.70
A+B	26	2.29^c^	0.72	27	3.38^c^	1.01

*^a,b,c^Different superscripts in a row or in a column indicate significant difference (*p* value <0.0001).*

#### Characterization of the Bacterial Communities

A total of 1,030,580 sequences were obtained by amplicon sequencing of the V3-V4 region of the 16S rRNA gene. After quality-checking, filtering and elimination of matrix-related (rainbow trout) reads and chimeras, a total of 330,473 reads were obtained. The samples with the lowest number of reads contained mainly rainbow trout related sequences, accounting for up to 96% of the reads, independently from the amount of DNA that could be extracted (Table 3). For some samples, rarefaction curves (Supplementary Figure 2) displayed no asymptote, suggesting that the bacterial community characterization sometimes was limited by the sequence number. The number of reads per sample ranged from 523 to 25,729 (Table 3). The abundance table is provided in Supplementary Table 2. The sequences were classified into 203 OTUs affiliated with a 97% similarity level. We were able to identify 93 different genera. The rest of the OTUs were multi-affiliated at the genus level. Only 5 OTUs were identified at the species level: *Chryseobacterium jeonii*, *Patulibacter minatonensis*, *Roseimicrobium gellanilyticum*, *Vagococcus fessus*, and *Yimella radicis* (Supplementary Table 2).

**TABLE 3:** Number of reads, affiliated OTUs, and DNA concentration (ng/μL) in each sample.

**Sample name**	**No of Reads**	**No of OTUs**	**DNA concentration (ng/μL)**	**Sample name**	**No of Reads**	**No of OTUs**	**DNA concentration (ng/μL)**
	
**A_Raceway**				**B_Raceway**			
	
A11R	523	19	2.45	B06R	1360	20	8.6
A10R	758	20	12.95	B05R	2007	35	3.55
A09R	1193	22	1.88	B12R	2097	16	3.25
A01R	1711	21	4.52	B13R	2268	45	17.29
A03R	1734	23	3.2	B10R	2510	40	2.3
A07R	1765	20	2.71	B01R	2537	36	3.84
A14R	1836	18	3.86	B03R	3146	51	2.42
A08R	2658	33	1.47	B14R	3325	33	40.34
A12R	2901	17	4.24	B02R	4552	34	5.27
A04R	3740	32	5.67	B07R	4780	38	4.83
A05R	4096	29	3.76	B08R	5076	40	12.95
A13R	4948	44	0.44	B04R	6711	58	1.99
A06R	6324	30	4.66	B09R	10270	53	3.49
A02R	9352	20	8.56	B11R	16973	43	3.52
	
**A_Factory**				**B_Factory**			
	
A19F	707	25	31.64	B15F	970	32	43.23
A18F	1261	29	28.94	B21F	569	17	21.8
A16F	1578	23	6.4	B22F	1603	15	5.9
A20F	1887	29	1.56	B20F	1606	17	7.37
A17F	2127	25	8.08	B28F	1901	19	107.24
A15F	4040	53	12.51	B25F	2215	22	10.01
A24F	7199	22	5.04	B24F	3019	18	3.28
A22F	13018	30	36.49	B27F	3630	34	26.7
A25F	16249	24	35.73	B19F	3914	20	28.14
A27F	18149	28	83.564	B26F	4482	32	6.17
A26F	19400	28	27.72	B23F	7452	28	31.13
A28F	19608	20	98.916	B18F	7689	18	23
A21F	21732	32	22.32	B17F	11443	32	58.65
A23F	25729	29	13.23	B16F	16145	20	31.39

**FIGURE 2:**
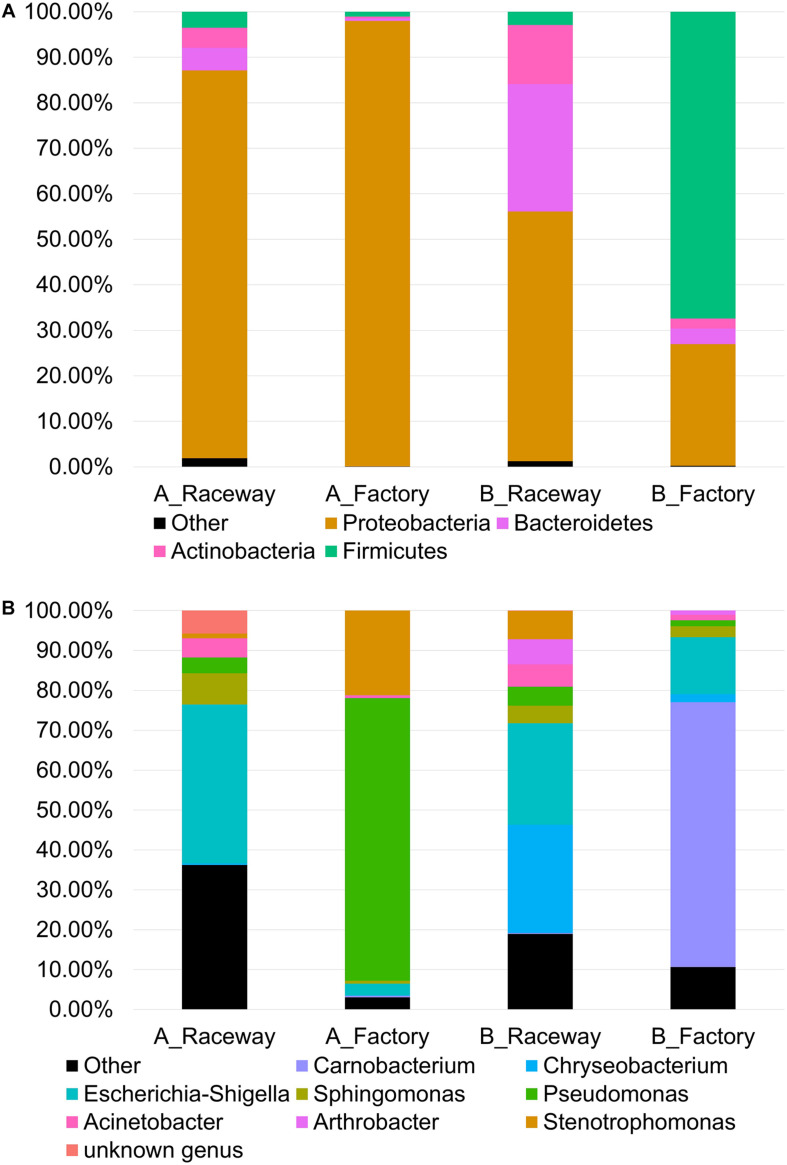
Cumulated histograms of the relative abundance (%) of the bacterial communities belonging to the most abundant **(A)** Phyla and **(B)** genera on fresh rainbow-trout fillets, depending on the farm and filleting conditions. Each histogram represents the sequences summed from the 14 fillets in each condition.

The bacterial communities of the fillets, regardless of the four sampling conditions (AR, AF, BR, BF), were globally dominated by Proteobacteria (71.96% of total sequences), mainly composed of Gamma-proteobacteria (62.21%) and Alpha-proteobacteria (6.69%), Firmicutes (15.14%), Bacteroidetes (7.33%), and Actinobacteria (4.94%) classes. Figure 2 shows the cumulated histograms of the relative abundance (%), obtained through aggregation of all sequences obtained for the 14 samples in each condition. The dominant phyla in each condition are shown in Figure 2A and the dominant genera in each condition are shown in Figure 2B. On farm A, the bacterial populations of the AR fillets were dominated by Gamma-proteobacteria (59.88%) represented by *Escherichia*-*Shigella* (39.87%), *Verticia* (5.98%), *Acinetobacter* (4.80%), and *Pseudomonas* (3.92%), and by Alpha-proteobacteria (24.98%) represented by an unknown genus belonging to the *Rickettsiaceae* family (7.84%) and *Sphingomonas* (7.83%). Bacteroidetes accounted for 4.97% of the bacterial populations and were represented by *Hymenobacter* (2.33%) and *Pedobacter* (1.53%). Actinobacteria accounted for 4.36% of the bacterial populations and were represented by *Rhodococcus* as the dominant genus of this phylum, and Firmicutes accounted for 3.53%. After factory-processing, AF fillets also were dominated by Gamma-proteobacteria, accounting for 96.19% of the bacterial populations and represented by *Pseudomonas* (70.82%), *Stenotrophomonas* (21.26%) and *Escherichia*-*Shigella* (2.91%), and by Alpha-proteobacteria, accounting for 1.78% of the bacterial populations.

On farm B, the bacterial populations of the BR fillets were dominated by Gamma-proteobacteria (45.90%) represented by *Escherichia*-*Shigella* (25.47%), *Acinetobacter* (5.63%), and *Pseudomonas* (4.70%), and by Alpha-proteobacteria (*Sphingomona*s, 4.42%). Bacteroidetes accounted for 28.02% of the bacterial populations and were mostly represented by *Chryseobacterium* (27.14%). Actinobacteria (12.98%) were represented by *Arthrobacter* (6.29%). After factory-processing, the bacterial populations of the BF fillets were dominated by Firmicutes (67.41%) represented by *Carnobacterium* (66.38%), then by Proteobacteria (25.88%) represented by *Escherichia*-*Shigella* (14.31%) and *Sphingomonas* (2.73%). The last two phyla detected were Bacteroidetes (3.44%) represented by *Chryseobacterium* (1.97%), and Actinobacteria (2.93%) represented by *Arthrobacter* (1.03%).

**FIGURE 3:**
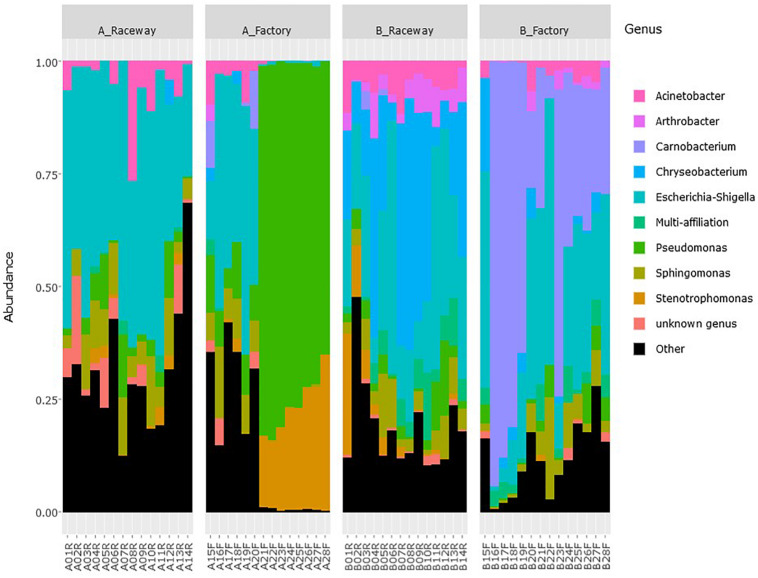
Relative abundance of the most abundant genera (top 10) on fresh rainbow-trout fillets, depending on the farm and filleting conditions. Each histogram represents a single fillet.

Although the bacterial microbiota of fillets from the same sampling condition were dominated by similar bacterial communities, OTUs had various relative abundances from one fillet to another (Figure 3).

**FIGURE 4:**
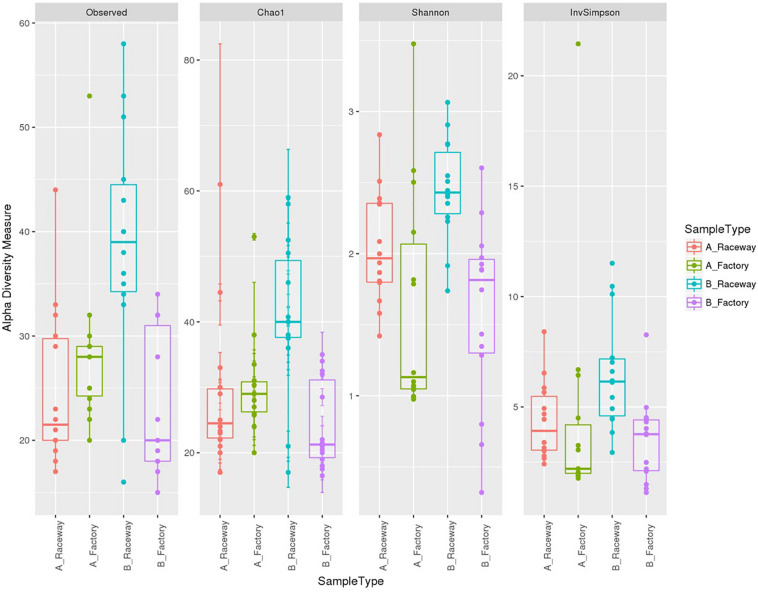
α-diversity indices with boxplots for all samples depending on the farm and filleting conditions. Each dot represents a single fillet.

The BR fillets harbored a larger amount of observed OTUs than fillets sampled from farm A (Figure 4). The Chao1 and Observed OTUs indices were similar for three out of four batches, indicating a good description of the bacterial communities. The upper Chao1 index values from the BR fillets suggested that a large amount of OTUs were possibly not detected in the fillets. The Shannon indices indicate that the factory-processed fillets (F) had a lower evenness of sequence repartition than the laboratory-processed fillets (R), regardless of the sampled farm. This suggests that the communities from the factory-processed fillets (F) were dominated by few taxa with a high relative abundance, namely *Pseudomonas*, *Stenotrophomonas* in fillets originating from farm A and *Carnobacterium* in fillets originating from farm B. The Inverse-Simpson indices displayed the same tendency and confirmed this observation (Figure 4).

**FIGURE 5:**
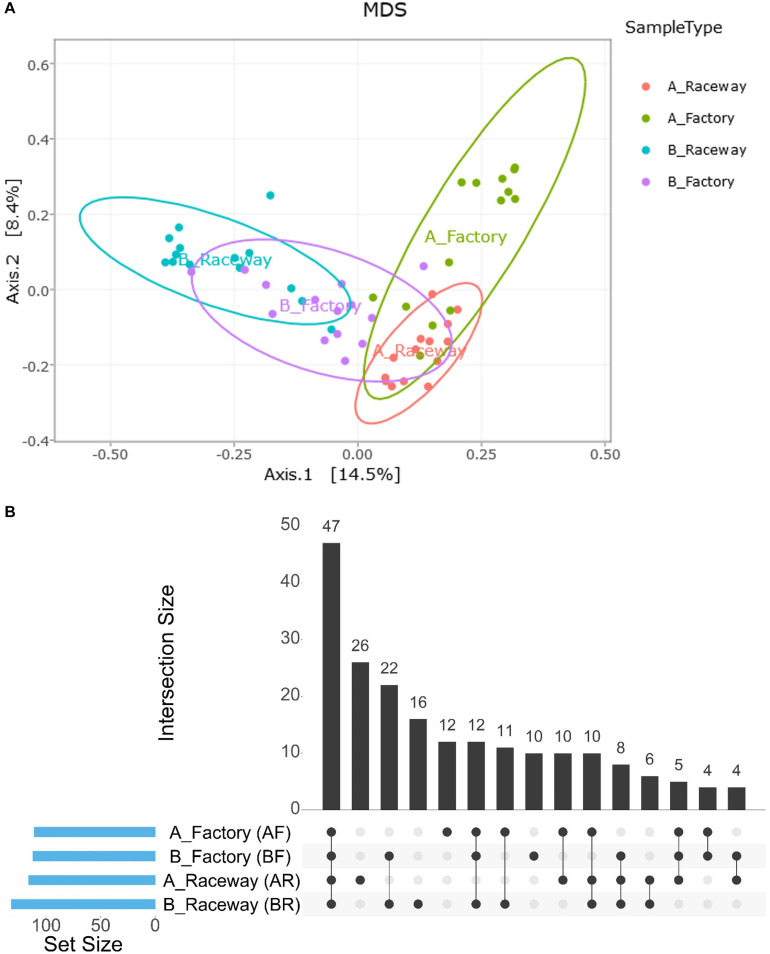
**(A)** Visualization of the impact of the farm and filleting conditions on the bacterial community structures of the fillets through MDS distribution of the samples’ β-diversity according to the Jaccard dissimilarity. Each dot represents a single fillet. **(B)** UpSet plot showing the intersection of OTUs depending on the farm localization and/or filleting condition. Blue bars in the left panel display the total number of OTUs per condition. The number of OTUs detected in one to four conditions is shown as barplots.

**FIGURE 6:**
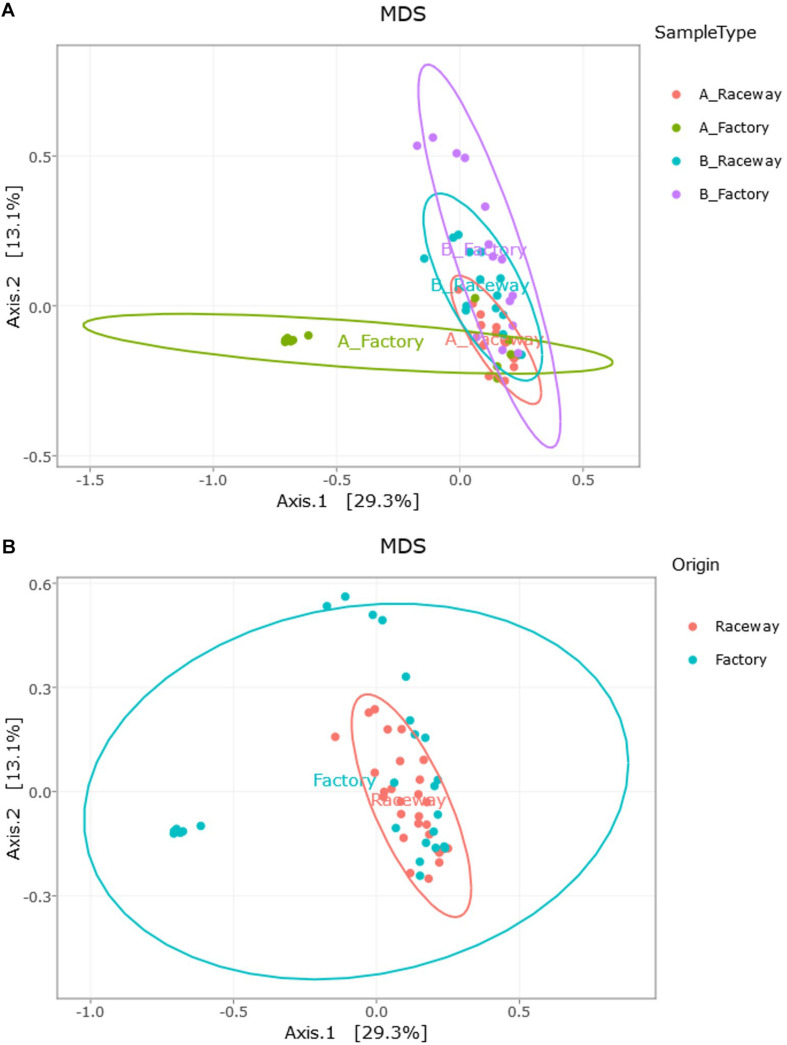
Visualization of the impact of **(A)** the farm and filleting conditions (Bray-Curtis dissimilarity); and of **(B)** the impact of the filleting conditions alone (Farm A and B combined, Bray-Curtis dissimilarity), through MDS distribution of the samples’ β-diversity. Each dot represents a single fillet.

The MDS visualization of the data showed slight bacterial community differences between fillets from farm A and farm B, and a separation regarding the filleting procedure. Moreover, the MDS highlights a potential common bacterial community structure (Figure 5A). Furthermore, PERMANOVA analysis based on Jaccard dissimilarity showed that the bacterial community structures were significantly different (*p* value <0.0001) regarding the different conditions (farm A or B, laboratory or factory-filleting). UpSet diagram showing OTUs intersection (Figure 5B) showed that out of the 203 affiliated OTUs, 47 were common to all conditions. OTUs specific to each conditions were also observed. Indeed, 26 OTUs were present only in the AR fillets, 16 only in the BR fillets, 12 only in the AF fillets and 10 only in the BF fillets. Furthermore, 22 OTUs were found only in fillets from farm B (BR and BF fillets), and 10 were found only in fillets from farm A (AR and AF fillets). Finally, 6 OTUs were specific to AR and BR fillets, and 4 were specific to the AF and BF fillets. The MDS visualization of the data based on the Bray-Curtis dissimilarity also showed that the bacterial community were differentially abundant depending on the conditions. PERMANOVA analysis confirmed this observation (*p* value <0.0001). In fact, the factory processing impacted the evenness observed, as *Pseudomonas* and *Stenotrophomonas* were significantly more abundant in AF fillets (*p* value <0.0001), and *Carnobacterium* were significantly more abundant in BF fillets (*p* value <0.0001) (Figure 6A). In fact, most of the fillets were closely related and some factory-processed fillets (F) appeared to separate from the main cluster (Figure 6B).

### Detection of Antibiotic Resistance Genes

**FIGURE 7:**
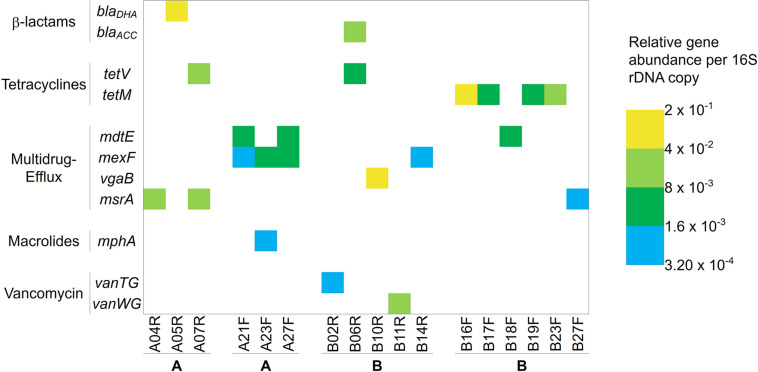
Antibiotic resistance genes detected and their relative abundance per 16S rDNA copy in the rainbow-trout fillets. Gene names are indicated on the left panel and samples at the bottom. Colored boxes indicate antibiotic resistance genes detected, with color scale ranging from blue (lowest abundance) to yellow (highest abundance), representing the relative abundance of the genes compared to the 16S rRNA gene.

The Smartchip Real-time PCR assays showed the presence of 11 antibiotic resistance genes out of the 248 tested, detected in 17/56 fillets, each fillet possessing one or two antibiotic resistance genes (Figure 7). The detected genes included β-lactam (*bla*_DHA_, *bla*_ACC_), tetracycline (*tetV* and *tetM*), macrolides (*mphA*), vancomycin (*vanTG* and *vanWG*) resistance genes and genes coding for multidrug-resistance or efflux pumps (*mdtE*, *msrA*, *mexF*, and *vgaB*). Among the 28 fillets originating from farm A, only six carried one or two detectable resistance genes, three from AR fillets and three from AF fillets. Concerning the AR fillets in which resistance genes were detected, one carried *msrA*, one *bla*_DHA_ and one carried both *msrA* and *tetV*, with relative abundances between 2 × 10^–1^ and 8 × 10^–3^ compared to the 16S rRNA gene. All three positive AF fillets carried two antibiotic resistance genes (two harbored *mdtE* and *mexF* and one harbored *mexF* and *mphA*), with relative abundances between 8 × 10^–3^ and 3.2 × 10^–4^ compared to the 16S rRNA gene. Thus overall, six different antibiotic resistance genes were detected in fillets originating from farm A. Eleven fillets from farm B harbored antibiotic resistance genes. Ten carried one detectable gene (*vanTG*, *vanWG*, *vgaB*, *mexF*, *tetV*, or *bla*_ACC_) and one carried two (*tetV* and *bla*_ACC_). Concerning the BR fillets, four carried one detectable resistance gene and one carried two. In addition, six BF fillets harbored either *tetM*, *mdtE*, or *msrA*. Relative abundances of the genes varied from 2 × 10^–1^ to 3.2 × 10^–4^ compared to the 16S rRNA gene copies. Overall, nine different antibiotic resistance genes were detected in fillets sampled from farm B. In the fillets B16F, B19F and B23F carrying a tetracycline resistance gene, OTC residues were also detected. In the fillets A07R, B02R, B06R, B14R, B17F, and B18F, OTC residues along with another resistance gene were detected.

**FIGURE 8:**
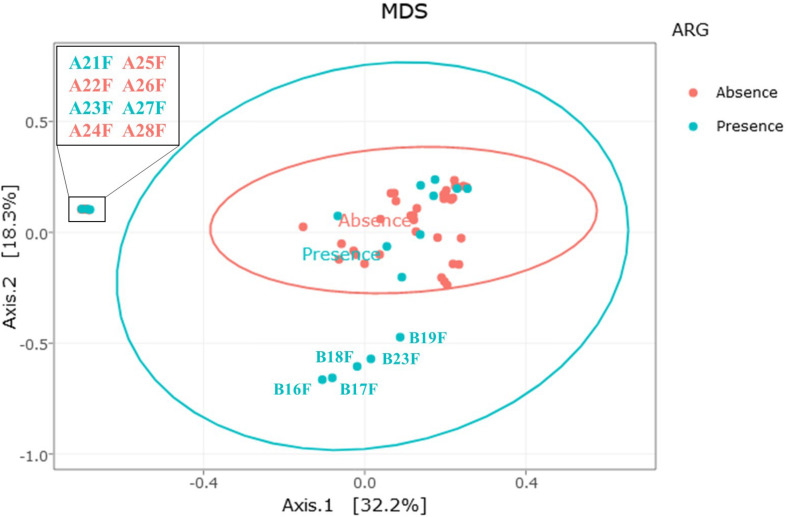
Relationship between the presence (blue dots) or absence (red dots) of antibiotic resistance genes and bacterial communities. MDS distribution is based on the Bray-Curtis dissimilarity index of β-diversity. Each dot represents a single fillet.

The multidimensional scaling (MDS) distribution of the bacterial community profiles, displaying the Bray-Curtis index and regarding the presence or absence of a detected antibiotic resistance gene is shown on Figure 8. Most of the samples appear to share similar communities, regardless of the absence or presence of a detected resistance gene. The five samples on the lower part of the figure (samples B16F, B17F, B18F, B19F and B23F) had both a distinct bacterial community structure and a detectable antibiotic resistance gene. Those samples were the factory-processed samples from farm B displaying a higher *Carnobacterium* abundance. Similarly, among the samples on the left part of the figure (A21F, A23F, and A27F) had a distinct bacterial community structure, namely a higher *Pseudomonas* abundance, and two detectable resistance genes. However, the other samples with a different bacterial community structure, namely samples A22F, A24F, A25F, A26F and A28F, also displayed a higher *Pseudomonas* abundance but did not carry a detectable resistance gene.

## Discussion

We hypothesized that the laboratory-processed fillets (R) carried a bacterial microbiota that mainly reflected the fish’s farming environment (raceway environment and breeding practices), whereas the factory-processed fillets (F) additionally encompassed contaminations originating from the factory and filleting process. Indeed, Giatsis et al. (2015) observed a correlation between the bacterial communities from water and tilapia larvae gut, and Sullam et al. (2012) showed as well that the rearing environment shaped the gut microbiota in fish. We observed that the bacterial communities of the raceway fillets from both farms were dominated by Proteobacteria and Bacteroidetes. This was consistent with the findings of Zhang X. et al. (2018) and Lowrey et al. (2015), who both described the skin-associated bacterial communities of rainbow trout to be dominated by Proteobacteria, Bacteroidetes and Firmicutes. Gamma-proteobacteria was the most represented class, probably due to the high prevalence of *Escherichia-Shigella* and *Pseudomonas*. However, Boutin et al. (2014), described Alpha-proteobacteria to be the most represented class in the skin bacterial microbiota of brook char (*Salvelinus fontinalis*), another river water trout belonging to the same subfamily as the rainbow trout (Salmoninae). The most prevalent genera in AR and BR fillets were *Escherichia-Shigella*, *Sphingomonas*, *Acinetobacter*, *Pseudomonas* and an unknown genus belonging to the *Rickettsiaceae* family. The genus *Shigella* is commonly associated with waterborne diseases (Cabral, 2010) and the genus *Escherichia* is a marker of fecal contamination (Harwood et al., 2014). A contamination of the fish by the river environment thus could explain their presence on the fillets from both farms. *Sphingomonas* species also are commonly found in water (Koskinen et al., 2000) and have been described in influent and tanks from salmon farms (Miranda and Zemelman, 2002). River water also can host *Acinetobacter* species (Doughari et al., 2011), which have been described in the intestinal content of rainbow trout (Guardabassi et al., 1999). Finally, *Pseudomonas* species are found in the skin-associated microbiota of rainbow trout (Zhang X. et al., 2018) and in aquaculture water (Fernández-Alarcón et al., 2010). In the findings of Zhang et al., the genera *Chryseobacterium*, *Pseudomonas* and *Acinetobacter* also were among the most represented genera, as well as an unidentified *Rickettsiaceae*.

The AR and BR fillets had communities with various relative abundances depending on the farm. We considered: (i) the age of the fish (14-months old or 26-months old), (ii) the geographical position of the farm (upstream or downstream from urban areas and wastewater treatment plants) and, (iii) the fasting time before sampling (2 days or 9 days) to explain these variations. The bacterial microbiota is known to be influenced by the age of the host during the first months of development (Zhang Z. et al., 2018). However, as far as we know no data are available regarding a change during the adult stage of farmed fish. The fillets from farm B had a higher relative abundance of *Chryseobacterium* and *Arthrobacter*. *Arthrobacter* spp. are commonly found in soil and wastewater effluents (Gobbetti and Rizzello, 2014), and *Chryseobacterium* spp. have also been described in soils (Benmalek et al., 2010) and from several fresh water systems (Loch and Faisal, 2015). Both genera could be more abundant in the BR fillets because of the presence of urban areas and wastewater treatment plants upstream from farm B. Such settlements may promote the enrichment and diversification of the bacterial microbiota of the rainbow trout fillets, as expressed by the higher α-diversity indices displayed by the BR fillets. The fasting time also could be a variation factor, as it has been described to increase the bacterial diversity in tilapia gut microbiota (Kohl et al., 2014). The Bacteroidetes phylum in the gut microbiota of seabass increased after eight days of fasting whereas the Flavobacteriales order (encompassing the *Chryseobacterium* genus) decreased after fasting (Xia et al., 2014). These results did not correlate with our findings. While we indeed observed an increase of Bacteroidetes, it was due to *Chryseobacterium* species.

Our α-diversity analysis showed a core-bacterial community shared by the fillets from all four conditions (47 OTUs), to which can be added 24 more OTUs shared by AR and BR fillets, constituting 71 OTUs present in AR and BR fillets. As this fillets were processed in sterile conditions, we can hypothesize that these 71 OTUs represent the native microbiota of fish fillets. Out of them, 49 were Proteobacteria (28 Gammaproteobacteria and 21 Alphaproteobacteria), 8 were Firmicutes (6 Bacilli and 2 Clostridia) and 8 were Actinobacteria. Condition-specific OTUs, each accounting for less than 400 cumulated reads, were observed in either AR (26 specific OTUs) or BR (16 specific OTUs) fillets. We observed that factory processing increased the bacterial load of the fillets and modified the structuration of the bacterial communities. Twenty-six OTUs were detected only in factory-processed fillets (AF, BF or both), compared to laboratory-processed ones. Nevertheless, each of them accounted for 500 cumulated reads. This is consistent with the finding of Møretrø et al. (2016) who described that the genera contaminating salmon fillets most likely originate from fish and environmental water. Actually, since no dominant OTU was detected in factory-processed fillets only, we concluded that the factory processing impacted the evenness more than the richness of the bacterial communities. This impact was different in the AF and the BF fillets, suggesting that different events could contribute to this modification.

A sudden shift appeared between fillets A20F and A21F (Figure 3). As samples were numbered in chronological order of processing, it is possible that an event occurred during the processing of fillet A20F. This shift was characterized by a dominance of *Pseudomonas* and *Stenotrophomonas*, two genera previously isolated from conveyor belts in salmon-processing plants (Langsrud et al., 2016). As bacteria from processing surfaces can be transferred to the fillets (Møretrø et al., 2016), our findings may thus result from a factory environment contamination originating from previous fish. The gutting process of the fish also could be the cause of this shift. In fact, both genera have been isolated from rainbow trout intestinal tracts (Wong et al., 2013). It is therefore possible that the shift we observed was due to conveyor and then fillet contamination by intestinal content. Concerning the fillets from farm B, we observed a higher abundance of *Carnobacterium* in factory-processed fillets, compared to the laboratory-processed ones. This genus has previously been isolated from conveyor belts (Langsrud et al., 2016). *Carnobacterium divergens* and *Carnobacterium maltaromaticum* are known as contaminant of food from animal origin including fish. They are also well adapted to gut and harbor adhesion capacity (Iskandar et al., 2017). It is therefore possible that fish processing resulted in an accumulation of *Carnobacterium* on the processing surfaces. This would be consistent with the findings of Chaillou et al. (2015) and Møretrø et al. (2016) who both described the bacterial communities of processed salmon fillets to be of both environmental and animal origins. It has to be noticed that fillets from farm A and B were processed on a different day, which could explain why we observed different contaminations.

We detected 11 different antibiotic resistance genes (*bla*_DHA_, *bla*_ACC_, *tetV*, *tetM*, *mdtE*, *mexF*, *vanTG*, *vanWG*, *vgaB*, *msrA* and *mphA*) in 17/56 fillets (Figure 7). In fillets originating from farm A, six different resistance genes were detected (*mphA*, *bla*_DHA_, *tetV*, *mdtE*, *mexF*, and *msrA*), in higher relative abundance in AR fillets than in AF fillets. In fillets originating from farm B, nine different resistance genes were detected (*bla*_ACC_, *tetV*, *tetM*, *vanTG*, *vanWG*, *mdtE*, *mexF*, *msrA*, and *vgaB*). The higher number of resistance genes in fillets from farm B could be explained by the presence of wastewater treatment plants between the two farms. Indeed, effluents from such plants have been described to increase the prevalence of antibiotic resistance genes and antibiotic resistant bacteria in rivers (Gordon et al., 2007; Rowe et al., 2016). Some of these genes have been described in aquatic and fish-related bacteria. *bla*_DHA_ has been described in river sediments (Dang et al., 2017), *bla*_ACC_ in *Aeromonas* spp. strains isolated from urban wastewater treatment plants (Piotrowska et al., 2017), and *tetM* in *Listeria innocua* isolated from catfish fillets and processing environments (Chen et al., 2010). *mphA* has been found in wastewater treatment plants, *vgaB* has been described in *Clostridium perfringens* isolates from water samples in South Africa (Fourie et al., 2019), and *msrA* in an *Enterococcus faecium* strain isolated from ready-to-eat raw fish (Hammad et al., 2014) and in the intestinal content of a farmed rainbow trout (Muziasari et al., 2017).

The spread of antibiotic resistance genes in the environment is a major issue. Some of the genes observed in this study could be associated with plasmids or mobile genetic elements, as previously shown for *mphA* (Su et al., 2020), *bla*_DHA_ (Barnaud et al., 1998), *msrA* (Ross et al., 1990), *tetM* (Fiedler et al., 2016), *vgaB* (Schwarz et al., 2015), and the *vanG* operon (Du et al., 2019).

Regarding the bacteria harboring the detected antibiotic resistance genes, Su et al. (2015) could correlate bacterial communities of sewage sludge and antibiotic resistome, as they observed up to 120 antibiotic resistance genes per sample. However, in our study fewer resistance genes detected in only a low number of fillets prevented us from performing such a statistical analysis. The fillets in which a resistance gene was detected did not clearly share a particular bacterial community profile. However, two outgroups were observed among the samples with antibiotic resistance genes. One included five samples dominated by *Carnobacterium*, with four positive for *tetM*. This resistance gene has previously been described in *Carnobacterium* species (Li and Wang, 2010). The second outgroup included 8 samples dominated by *Pseudomonas*, out of which three exhibited *mexF*, a gene conferring multidrug resistance to *Pseudomonas aeruginosa* (Köhler et al., 1997). Two of them also harbored *mdtE* and one harbored *mphA*. Those data may suggest that the afore mentioned antibiotic resistance genes could be carried by *Carnobacterium* in BF fillets or *Pseudomonas* in AF fillets.

We quantified OTC residues only at low concentrations (below 100 μg/kg) and in fillets from farm B. This was consistent with an OTC treatment applied to the fish 192 days prior to the sampling, thus respecting the mandatory withdrawal period between the treatments and slaughtering (500 degree-days), and complying with the maximum OTC residue limit. We hypothesized that antibiotic residues persisted in the sediments of the raceway and caused a low continuous exposure of the fish. This is consistent with previous data on the persistence of antibiotic in sediments. In marine sediments, OTC has a half-life of 151 to over 300 days, depending on the depth of sampling (Hektoen et al., 1995). We detected in five fillets the tetracycline resistance genes tetM or tetV, which as we above suggested could be carried by Carnobacterium. It is also possible that these genes were selected or spread among the bacterial communities due to the OTC treatment. The other genes, unrelated to antibiotic treatment history, could be naturally present in the bacteria or selected by anterior undocumented antibiotic pressure in the environment, then transferred to the fish-associated bacterial communities. The presence of resistance genes unrelated to selective pressure has indeed been documented (Looft et al., 2012). The detection of antibiotic resistance genes depends greatly on their copy number.

Our sampling plan based on the Cannon and Roe (1982) epidemiological guidelines, was dedicated to observe an event (presence of an antibiotic resistance gene or a bacterial population) whose prevalence in the entire raceway population was 20%, with 95% confidence. Thus, the detection of antibiotic resistance genes on one or more fillets per batch enabled to assess they had a prevalence of at least 20% in the entire fish raceway population. We detected only a few genes which displayed high C_T_ values (between 24 and 27), revealing their presence at low concentrations in the DNA extracts of the fillets. We cannot exclude the presence of other genes present at very low copy number or low prevalence in the population, that we may not have been able to detect.

To conclude, we were able to observe the existence of a shared bacterial community on the fresh rainbow trout fillets. We also observed bacterial community variations depending on the farm localization or processing conditions. These variations were due to low abundance OTUs. We hypothesized that the microbiota of fresh farmed rainbow trout fillets is most likely shaped by the river environment, which was common to both farms. In our case, it did not seem to be strongly influenced by the effluents from human activities (urban areas, wastewater treatment plants) localized between farm A and B. The OTU evenness variations we observed in factory-processed fillets might result from surface contaminations during the filleting process. The presence of antibiotic resistance genes seems to be influenced by the farming environment, including farming practices and river effluents. Further studies would be necessary to assess the phenotypic antibiotic resistance expressed by some species or genera of interest. The correlation of this phenotypic expression to genotypic features could provide complementary insights into the resistance potential of the bacterial communities on the rainbow trout fillets.

## Data Availability Statement

The datasets presented in this study can be found in online repositories. The names of the repository/repositories and accession number(s) can be found below: https://www.ebi.ac.uk/ena, PRJEB38652.

## Author Contributions

NH, SC, CM, and HP: experimental design, data analysis, and manuscript drafting and revision. NH and SC: sampling. NH, SC, AB-A, AR, and CM: sample processing. NH, AB-A, and AR: bacterial DNA extraction and enumeration of bacterial counts. DH-P: antibiotic residues screening, data acquisition and analysis, revising the manuscript. MZ: amplicon sequencing analysis design and data analysis, and drafting and revising the manuscript. AM: statistical analysis and data analysis, and manuscript revision. All authors contributed to the article and approved the submitted version.

## Conflict of Interest

The authors declare that the research was conducted in the absence of any commercial or financial relationships that could be construed as a potential conflict of interest.

## Supplementary Material

Supplementary Figure 1 PCR targeting gene *tuf* data. Standard curves chart, amplification chart, melting curve chart and melting peak chart for each samples. Bacterial loads were calculated from standard curves as described in 3.4.

Supplementary Figure 2 16S DNA sequencing Rarefaction curves for each sample.

Supplementary Table 1 List of primers used in the qPCR array. Primer names indicate the targeted genes.

Supplementary Table 2 Abundance table of the different OTUs obtained for each sample.
