# Supplementary material for: Antibiotic Resistance Genes and Bacterial Communities of Farmed Rainbow Trout Fillets (Oncorhynchus mykiss)
Source: Front Microbiol. 2020 Dec 3;11:590902. doi: 10.3389/fmicb.2020.590902 (PMC7744637; doi:10.3389/fmicb.2020.590902)

Supplementary Figures

Figure S1: PCR targeting gene tuf data: Standard curves chart, amplification chart, melting curve chart and melting peak chart for each samples. Bacterial loads were calculated from standard curves as described in 3.4.

Figure S2: 16S DNA sequencing : Rarefaction curves for each sample

Figure S1: PCR targeting gene *tuf* data : Standard curves chart, amplification chart, melting curve chart and melting peak chart for each samples. Bacterial loads were calculated from standard curves as described in 3.4. For each chart, standards are shown in red, negative controls are shown in black and samples are shown in green.

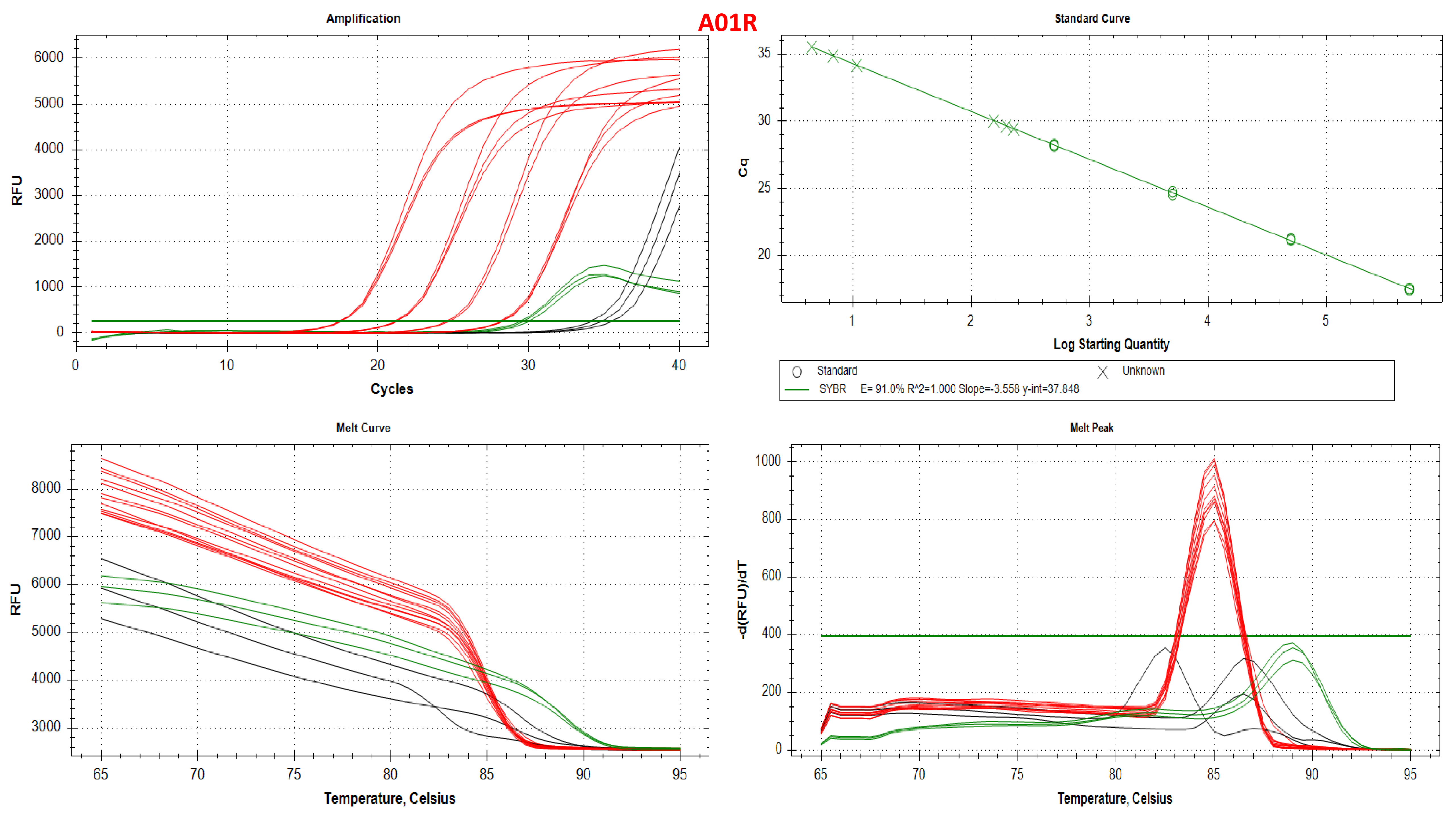

A01R
Amplification
RFU
Cycles
Standard Curve
Cq
Log Starting Quantity
Standard
Unknown
SYBR E= 91.0% R^2=1.000 Slope=-3.558 y-int=37.848
Melt Curve
RFU
Temperature, Celsius
Melt Peak
-d(RFU)/dT
Temperature, Celsius

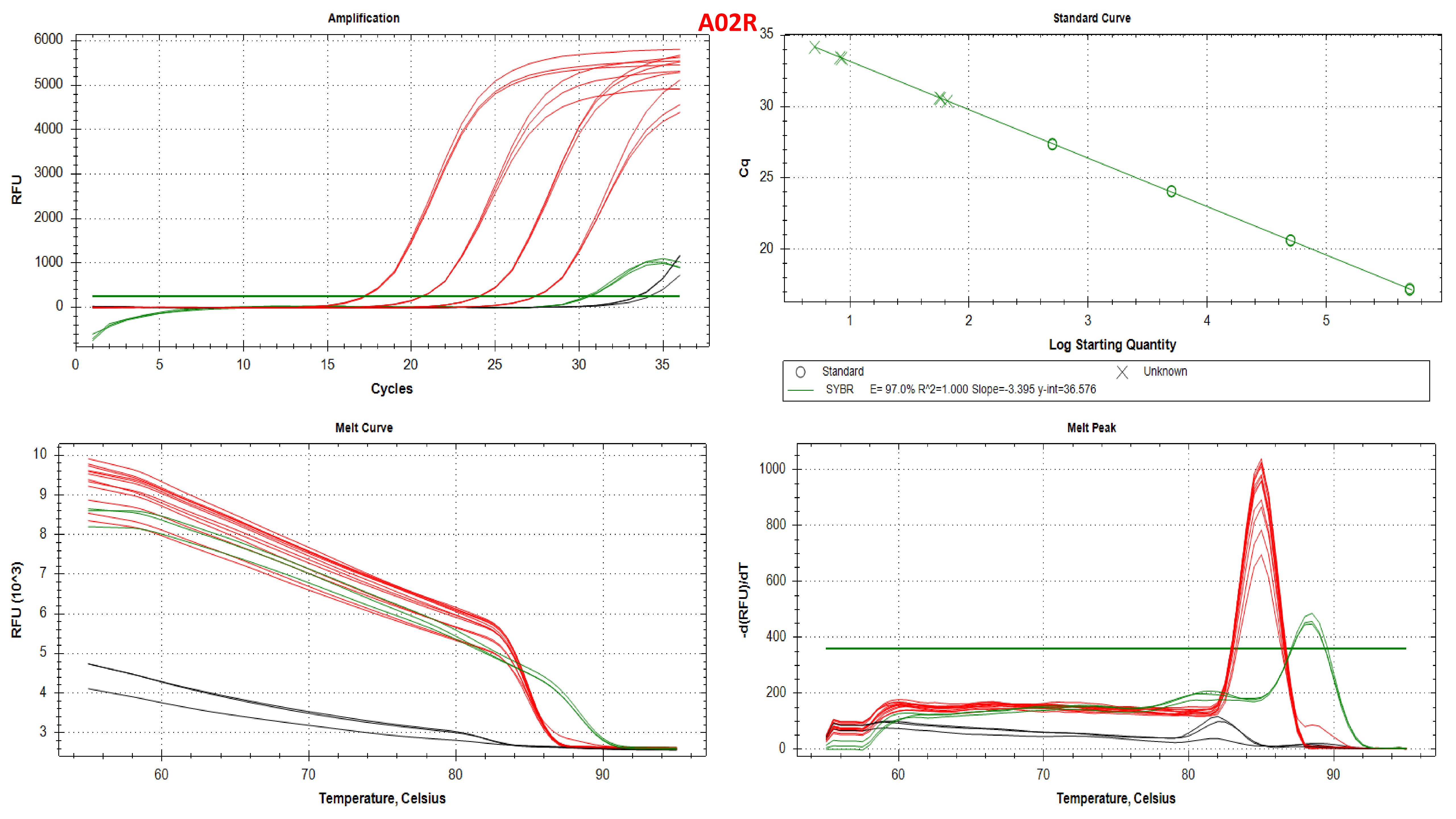
A02R
Amplification
RFU
Cycles
Standard Curve
Cq
Log Starting Quantity
Standard
Unknown
SYBR E= 97.0% R^2=1.000 Slope=-3.395 y-int=36.576
Melt Curve
RFU (10^3)
Temperature, Celsius
Melt Peak
-d(RFU)/dT
Temperature, Celsius

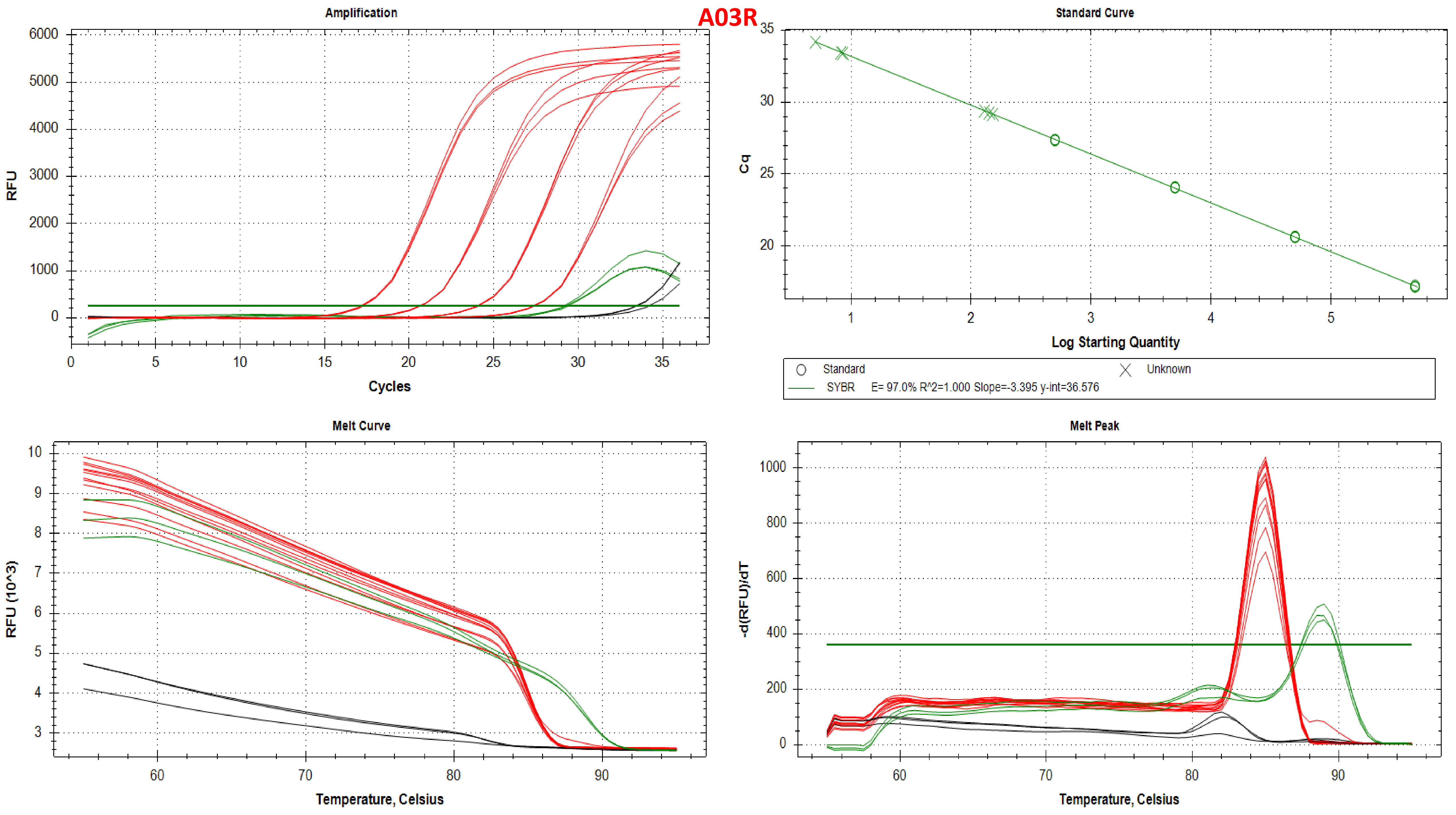

A03R
Amplification
RFU
6000
5000
4000
3000
2000
1000
0
0
5
10
15
20
25
30
35
Cycles
Standard Curve
Cq
35
30
25
20
1
2
3
4
5
Log Starting Quantity
Standard
Unknown
SYBR E= 97.0% R^2=1.000 Slope=-3.395 y-int=36.576
Melt Curve
RFU (10^3)
10
9
8
7
6
5
4
3
60
70
80
90
Temperature, Celsius
Melt Peak
-d(RFU)/dT
1000
800
600
400
200
0
60
70
80
90
Temperature, Celsius

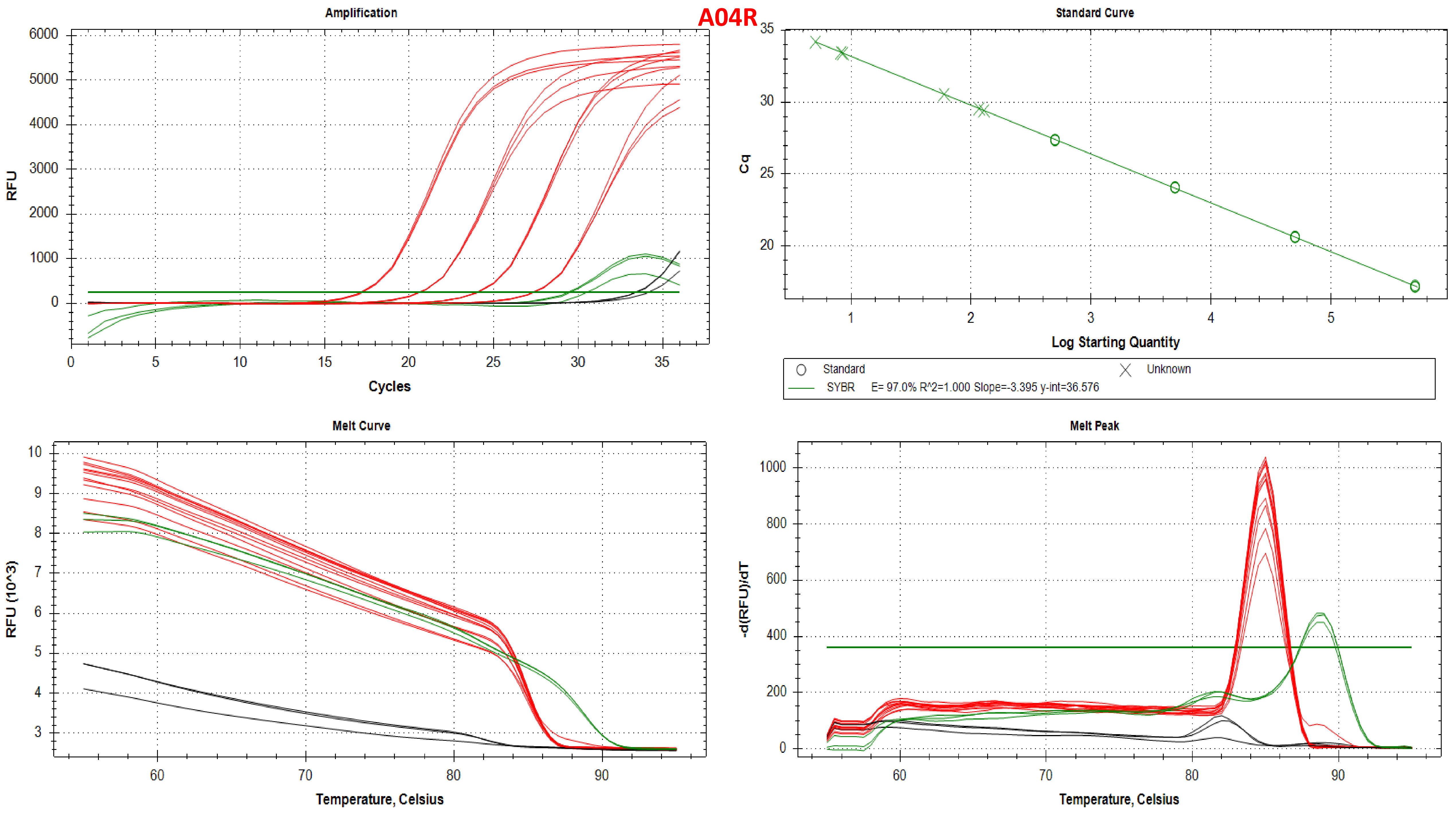

A04R
Amplification
RFU
Cycles
Standard Curve
Cq
Log Starting Quantity
Standard
Unknown
SYBR E= 97.0% R^2=1.000 Slope=-3.395 y-int=36.576
Melt Curve
RFU (10^3)
Temperature, Celsius
Melt Peak
-d(RFU)/dT
Temperature, Celsius

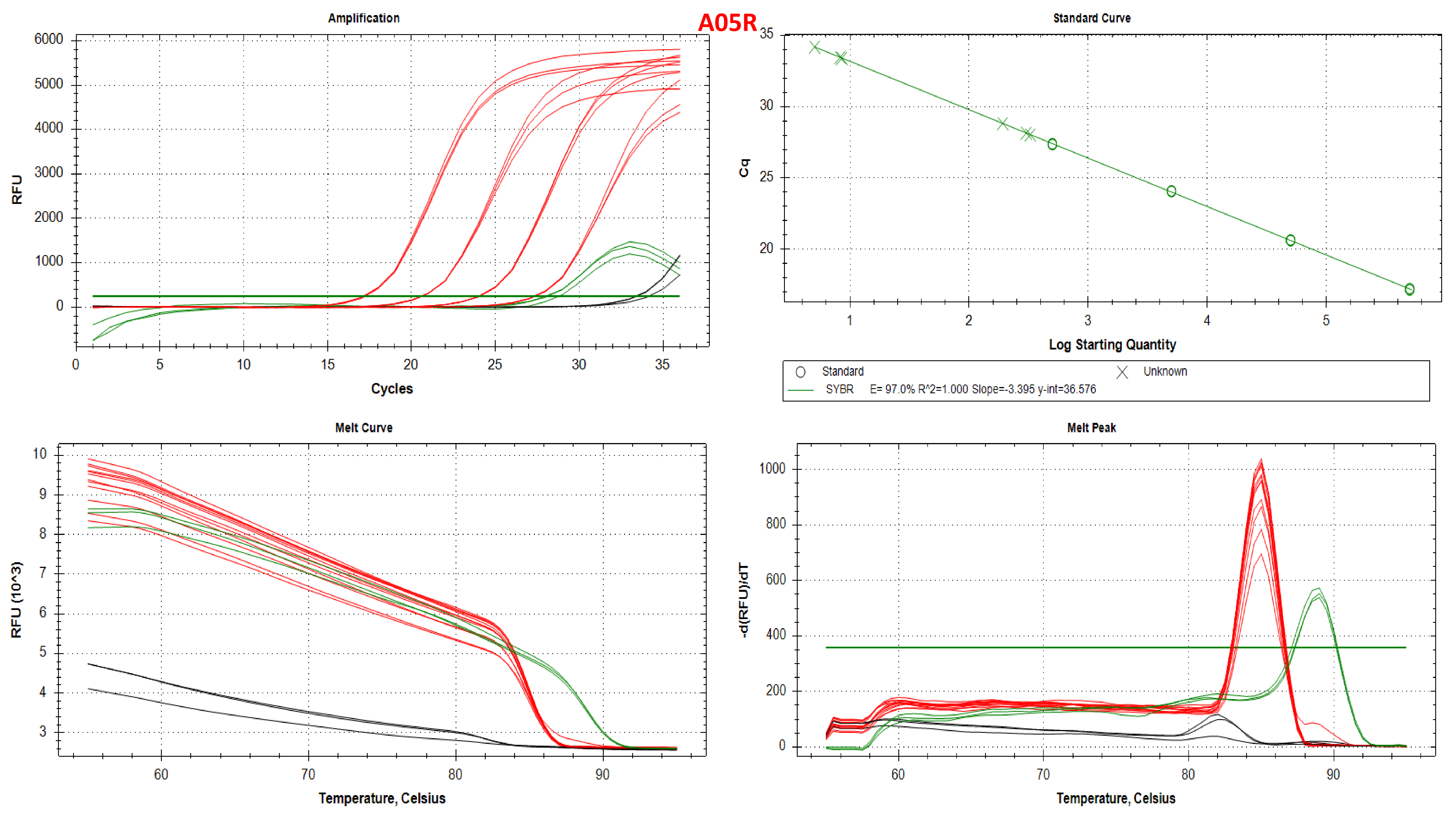
A05R
Amplification
RFU
Cycles
Standard Curve
Cq
Log Starting Quantity
Standard
Unknown
SYBR E= 97.0% R^2=1.000 Slope=-3.395 y-int=36.576
Melt Curve
RFU (10^3)
Temperature, Celsius
Melt Peak
-d(RFU)/dT
Temperature, Celsius

A06R

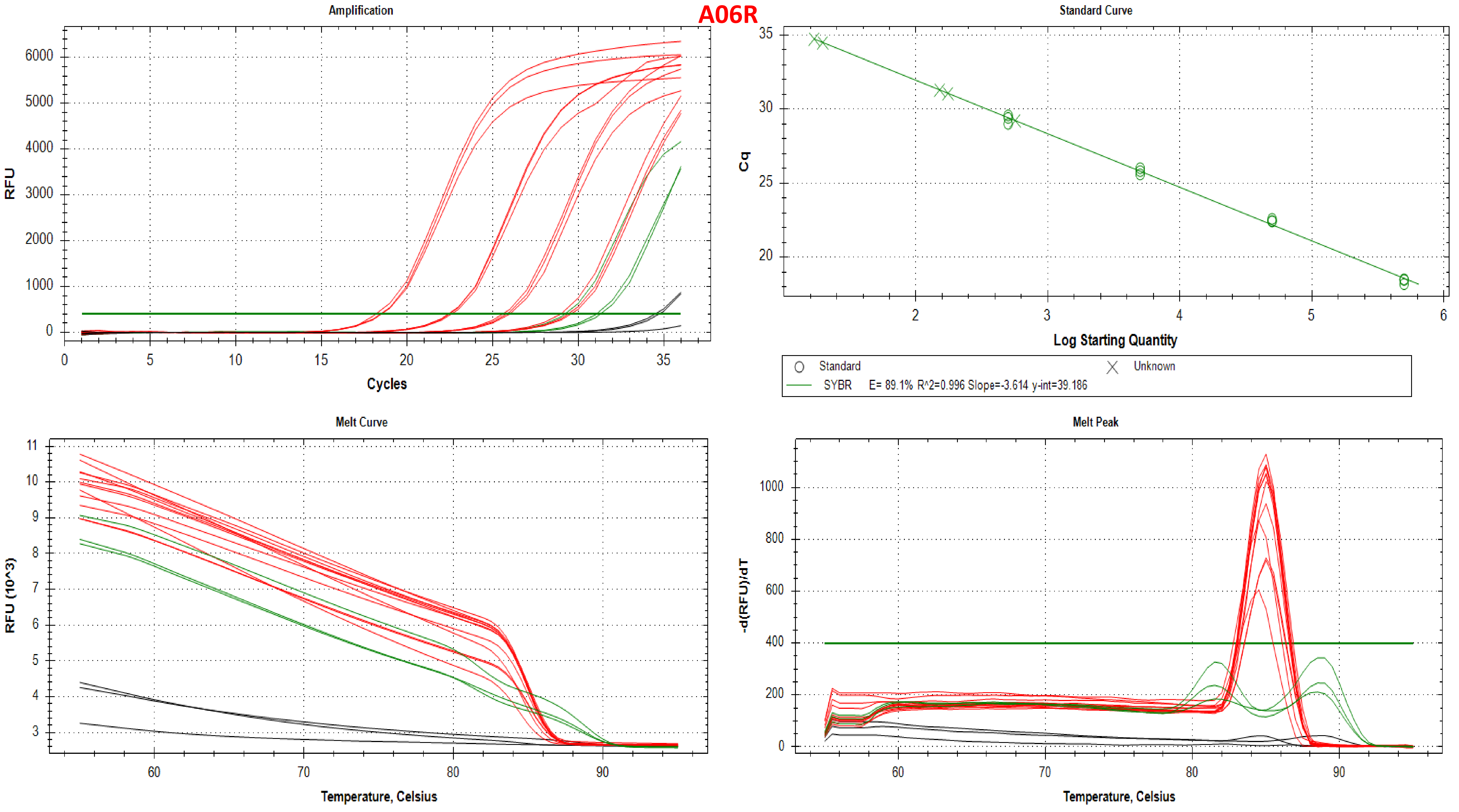

Amplification
RFU
Cycles
Standard Curve
Cq
Log Starting Quantity
Standard
Unknown
SYBR E= 89.1% R^2=0.996 Slope=-3.614 y-int=39.186
Melt Curve
RFU (10^3)
Temperature, Celsius
Melt Peak
-d(RFU)/dT
Temperature, Celsius

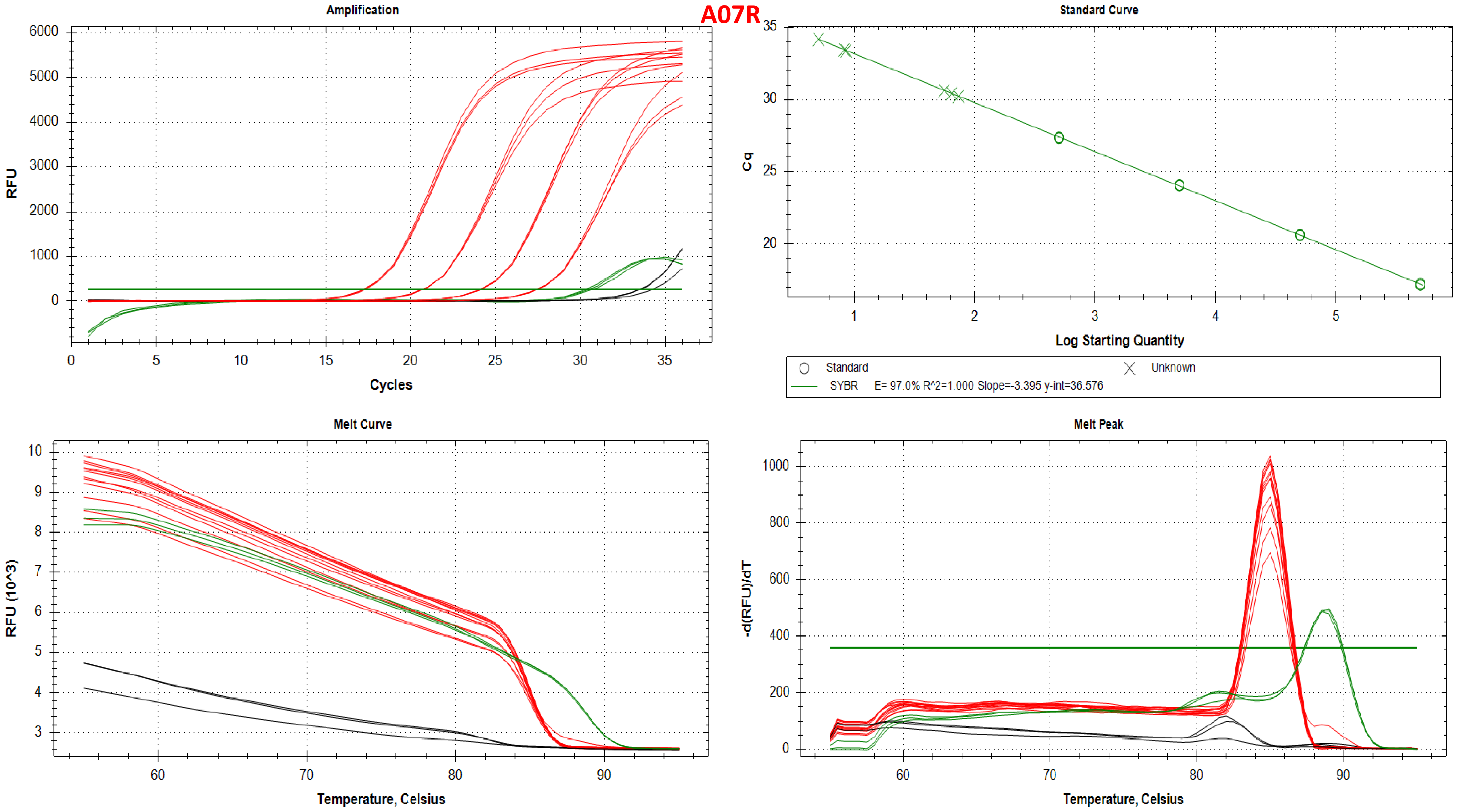
A07R
Amplification
RFU
Cycles
0
1000
2000
3000
4000
5000
6000
0
5
10
15
20
25
30
35
Standard Curve
Cq
Log Starting Quantity
20
25
30
35
1
2
3
4
5
Standard
Unknown
SYBR
E= 97.0% R^2=1.000 Slope=-3.395 y-int=36.576
Melt Curve
RFU (10^3)
Temperature, Celsius
3
4
5
6
7
8
9
10
60
70
80
90
Melt Peak
-d(RFU)/dT
Temperature, Celsius
0
200
400
600
800
1000
60
70
80
90

A08R

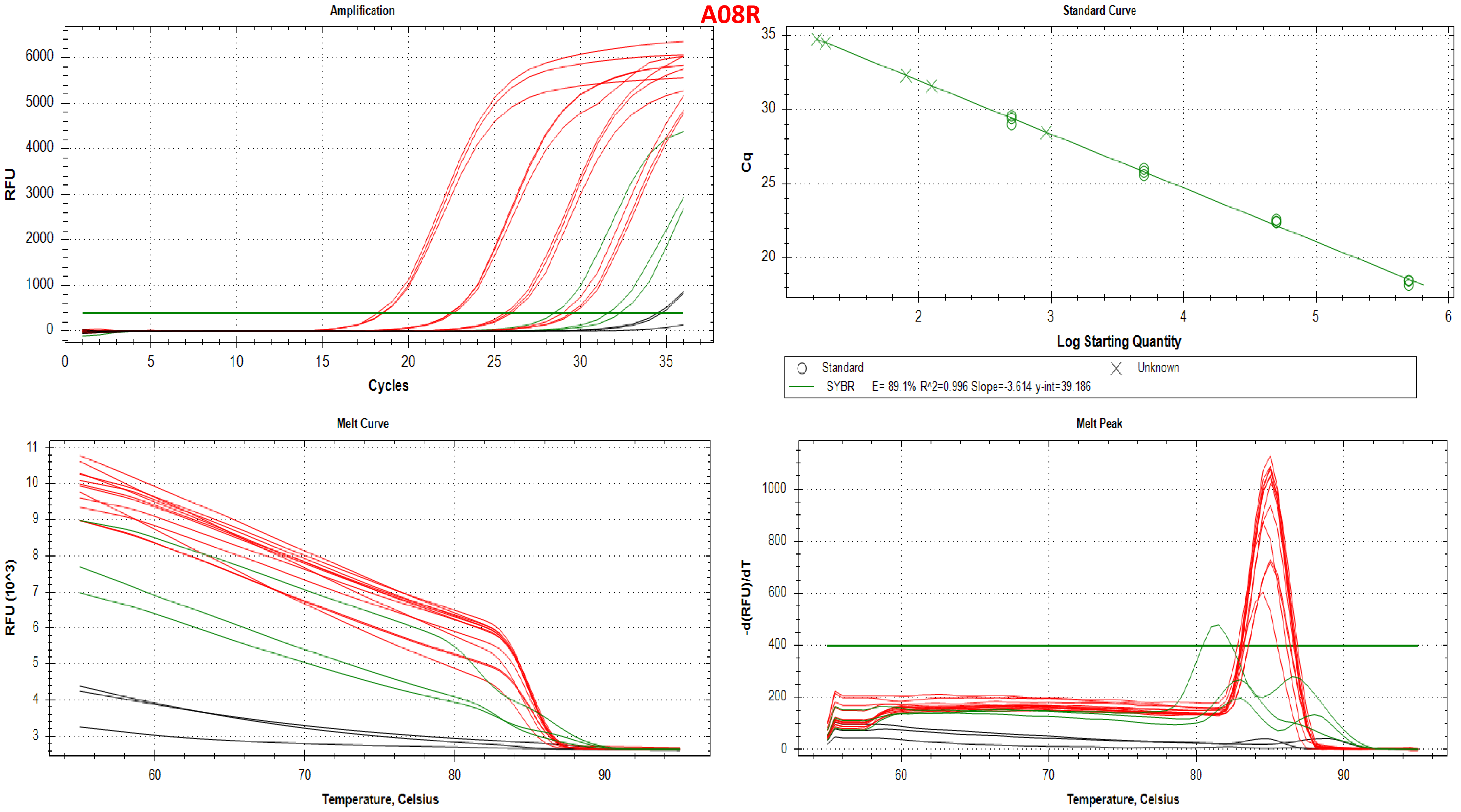
Amplification
RFU
Cycles
Standard Curve
Cq
Log Starting Quantity
Standard
Unknown
SYBR E= 89.1% R^2=0.996 Slope=-3.614 y-int=39.186
Melt Curve
RFU (10^3)
Temperature, Celsius
Melt Peak
-d(RFU)/dT
Temperature, Celsius

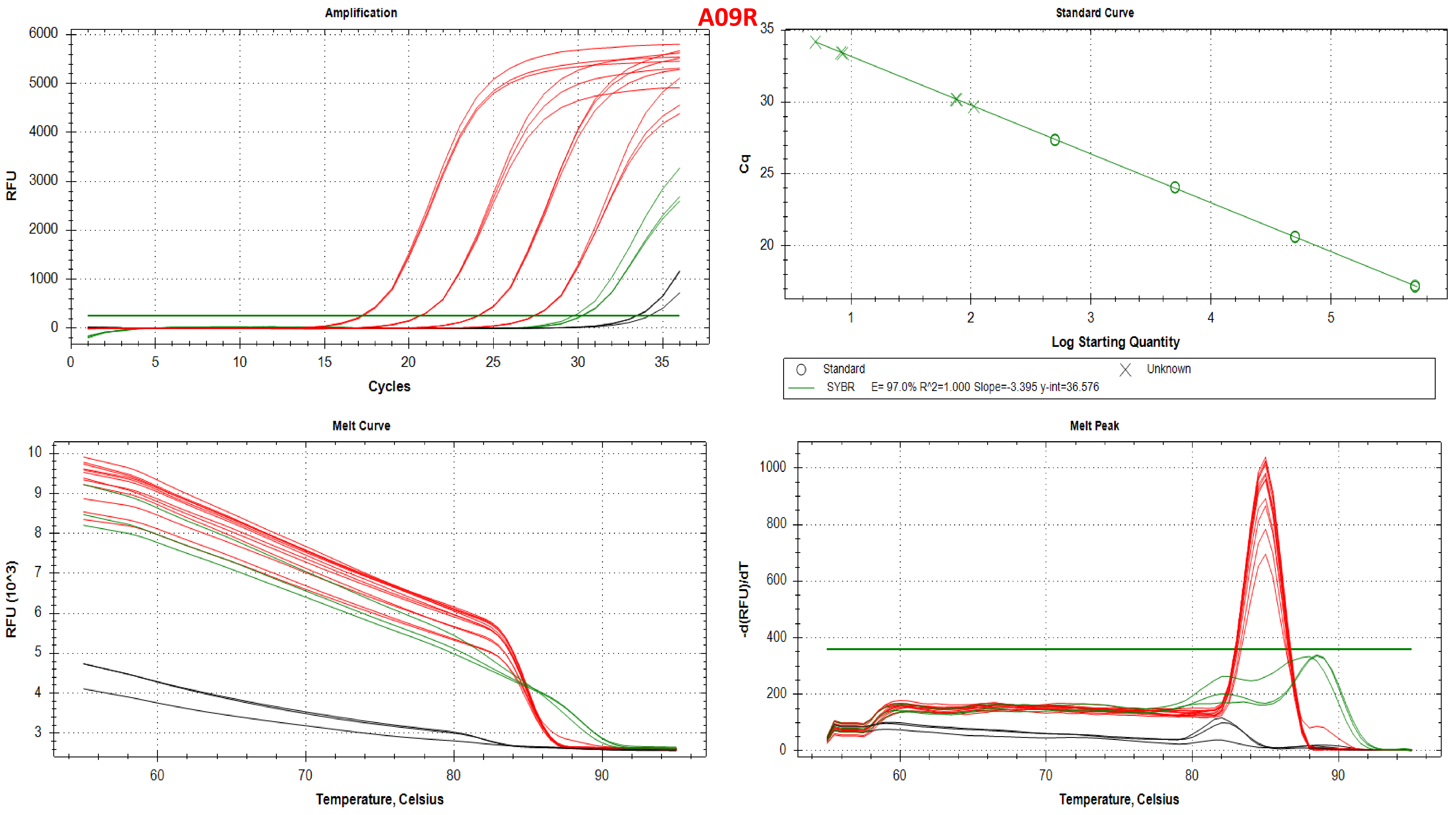
A09R
Amplification
RFU
Cycles
Standard Curve
Cq
Log Starting Quantity
Standard
Unknown
SYBR E= 97.0% R^2=1.000 Slope=-3.395 y-int=36.576
Melt Curve
RFU (10^3)
Temperature, Celsius
Melt Peak
-d(RFU)/dT
Temperature, Celsius

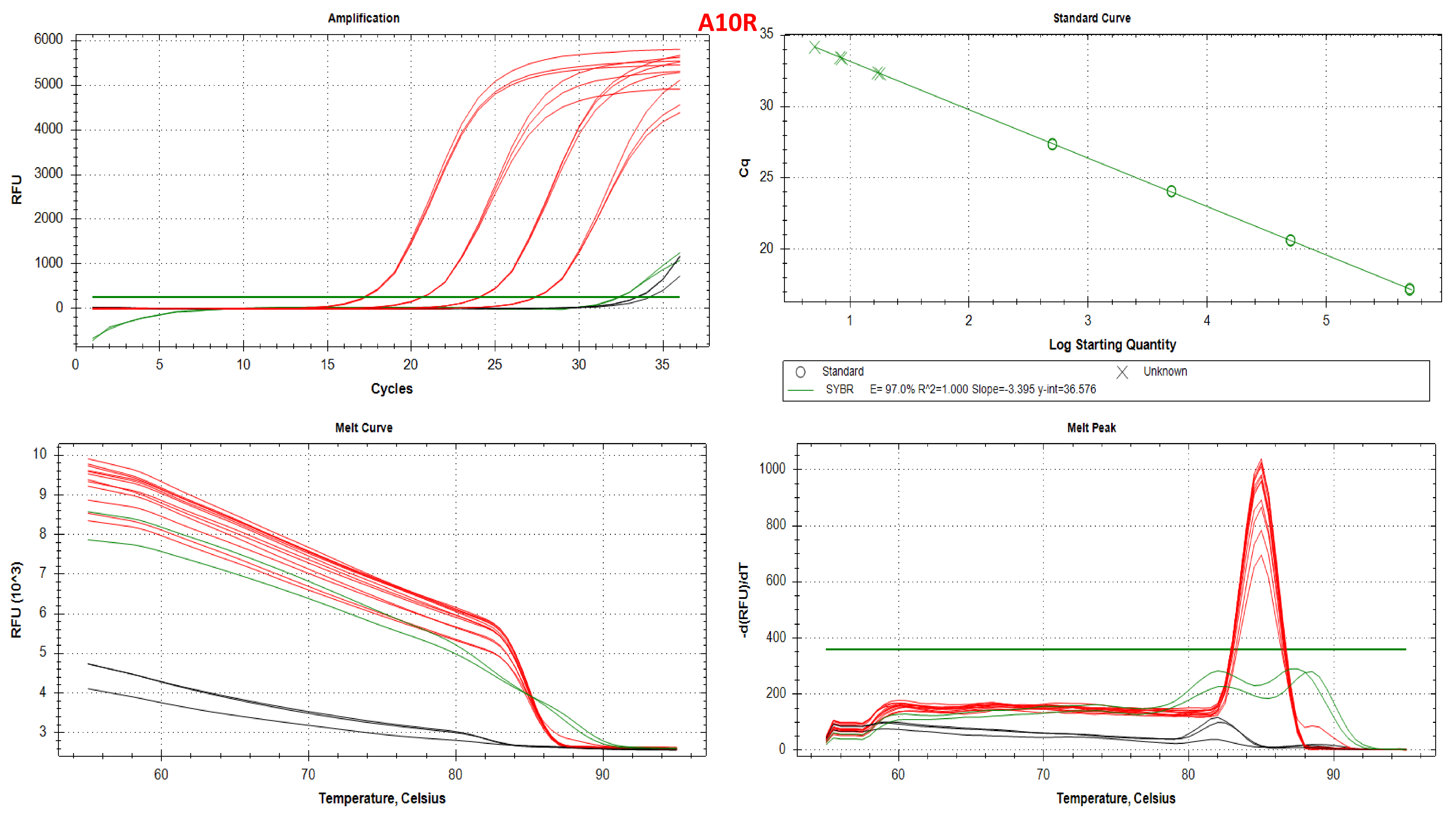
A10R
Amplification
RFU
Cycles
Standard Curve
Cq
Log Starting Quantity
Standard
Unknown
SYBR E= 97.0% R^2=1.000 Slope=-3.395 y-int=36.576
Melt Curve
RFU (10^3)
Temperature, Celsius
Melt Peak
-d(RFU)/dT
Temperature, Celsius

A11R

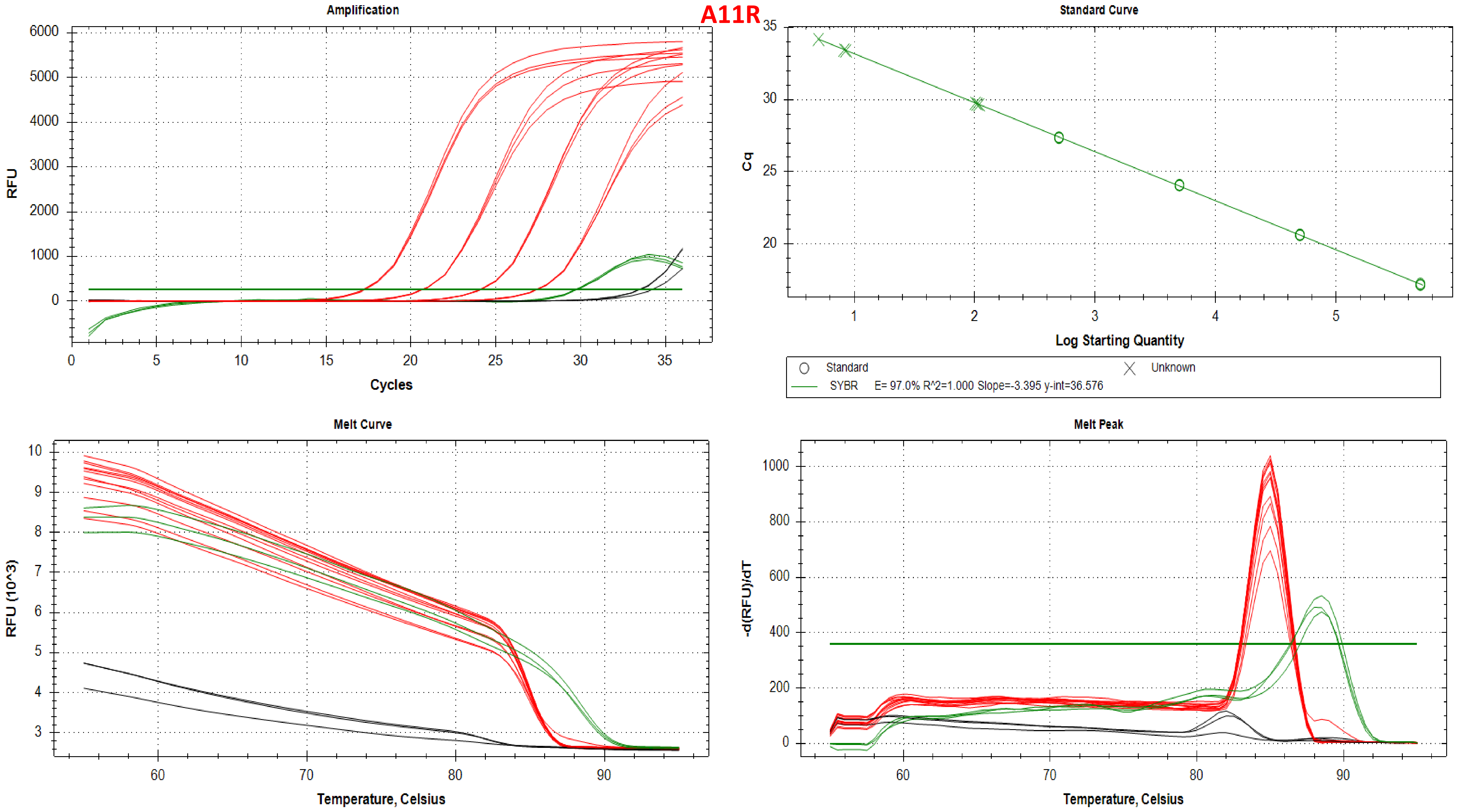
Amplification
RFU
Cycles
Standard Curve
Cq
Log Starting Quantity
Standard
Unknown
SYBR E= 97.0% R^2=1.000 Slope=-3.395 y-int=36.576
Melt Curve
RFU (10^3)
Temperature, Celsius
Melt Peak
-d(RFU)/dT
Temperature, Celsius

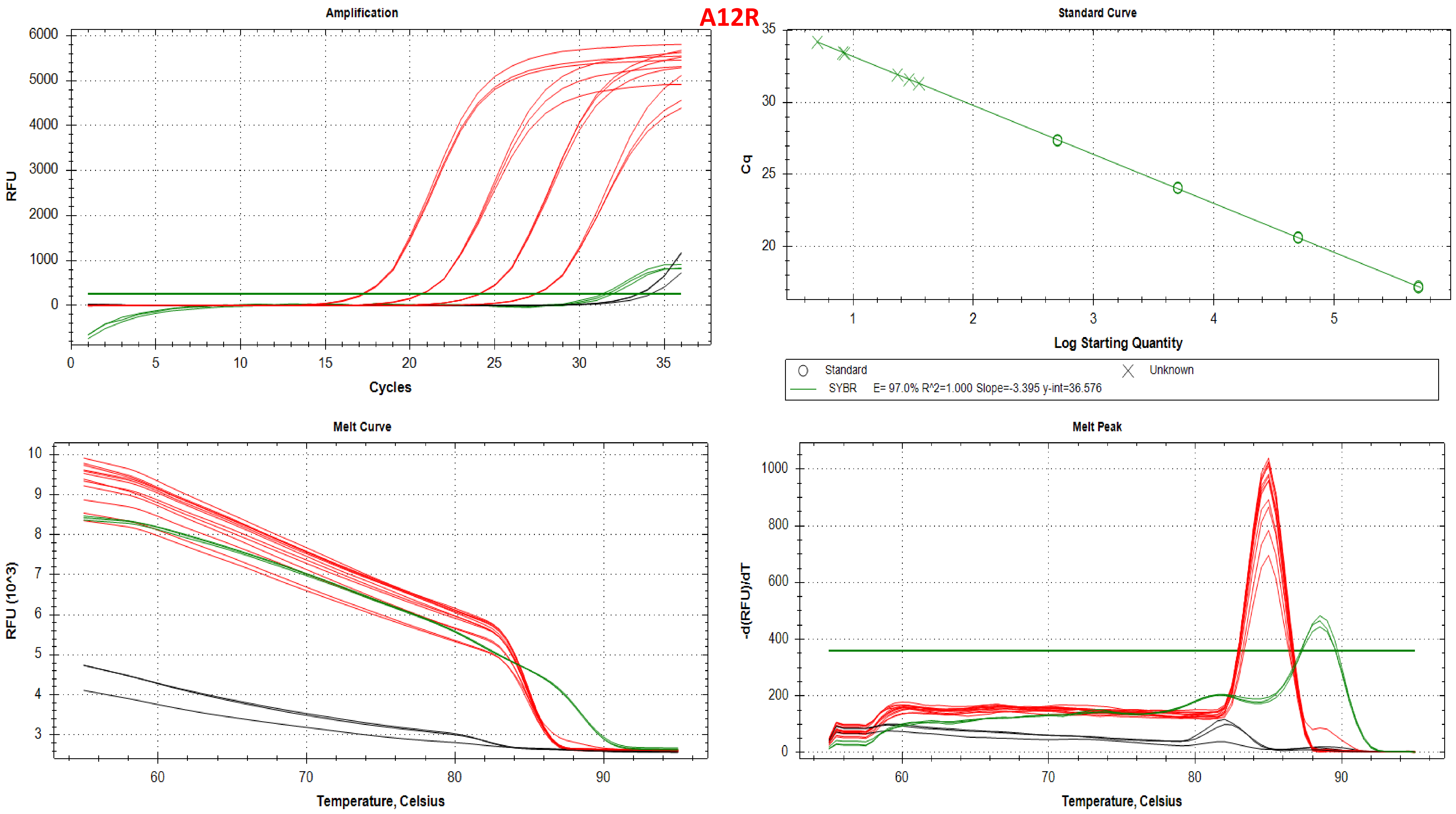
A12R
Amplification
RFU
Cycles
Standard Curve
Cq
Log Starting Quantity
Standard
Unknown
SYBR E= 97.0% R^2=1.000 Slope=-3.395 y-int=36.576
Melt Curve
RFU (10^3)
Temperature, Celsius
Melt Peak
-d(RFU)/dT
Temperature, Celsius

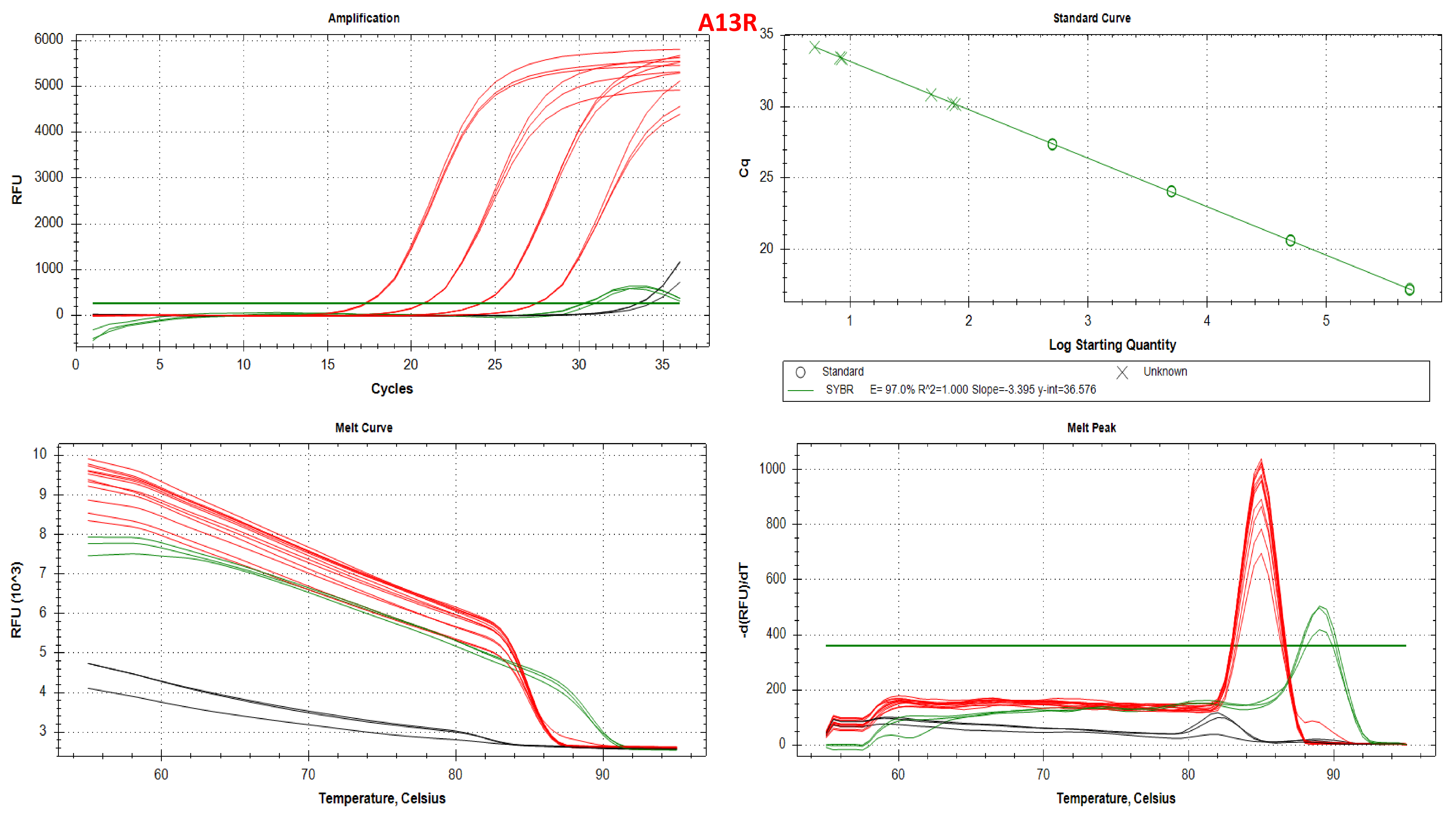
A13R
Amplification
RFU
Cycles
Standard Curve
Cq
Log Starting Quantity
Standard
Unknown
SYBR E= 97.0% R^2=1.000 Slope=-3.395 y-int=36.576
Melt Curve
RFU (10^3)
Temperature, Celsius
Melt Peak
-d(RFU)/dT
Temperature, Celsius

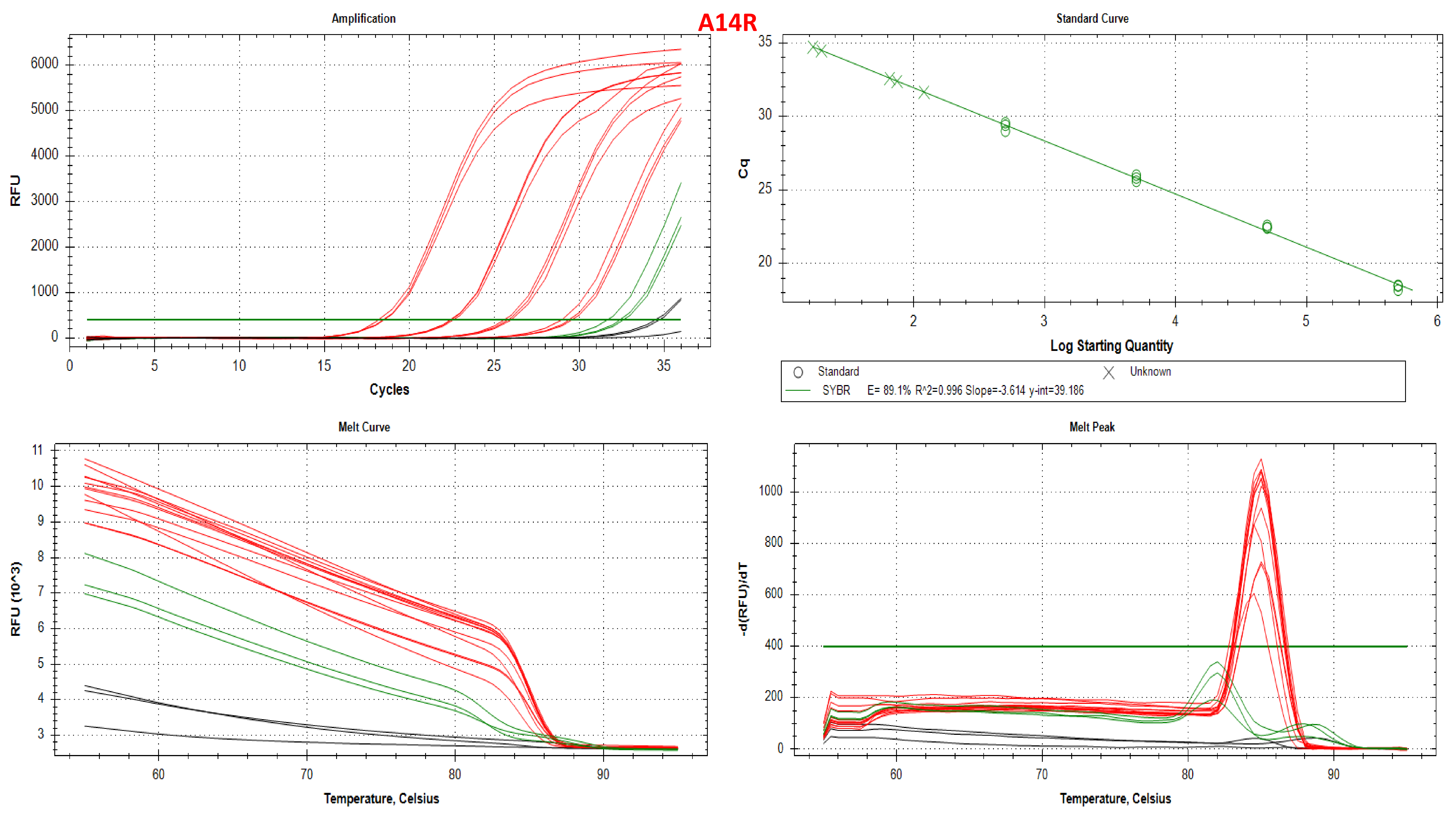

A14R
Amplification
RFU
Cycles
0
1000
2000
3000
4000
5000
6000
0
5
10
15
20
25
30
35
Standard Curve
Cq
Log Starting Quantity
20
25
30
35
2
3
4
5
6
Standard
Unknown
SYBR E= 89.1% R^2=0.996 Slope=-3.614 y-int=39.186
Melt Curve
RFU (10^3)
Temperature, Celsius
3
4
5
6
7
8
9
10
11
60
70
80
90
Melt Peak
-d(RFU)/dT
Temperature, Celsius
0
200
400
600
800
1000
60
70
80
90

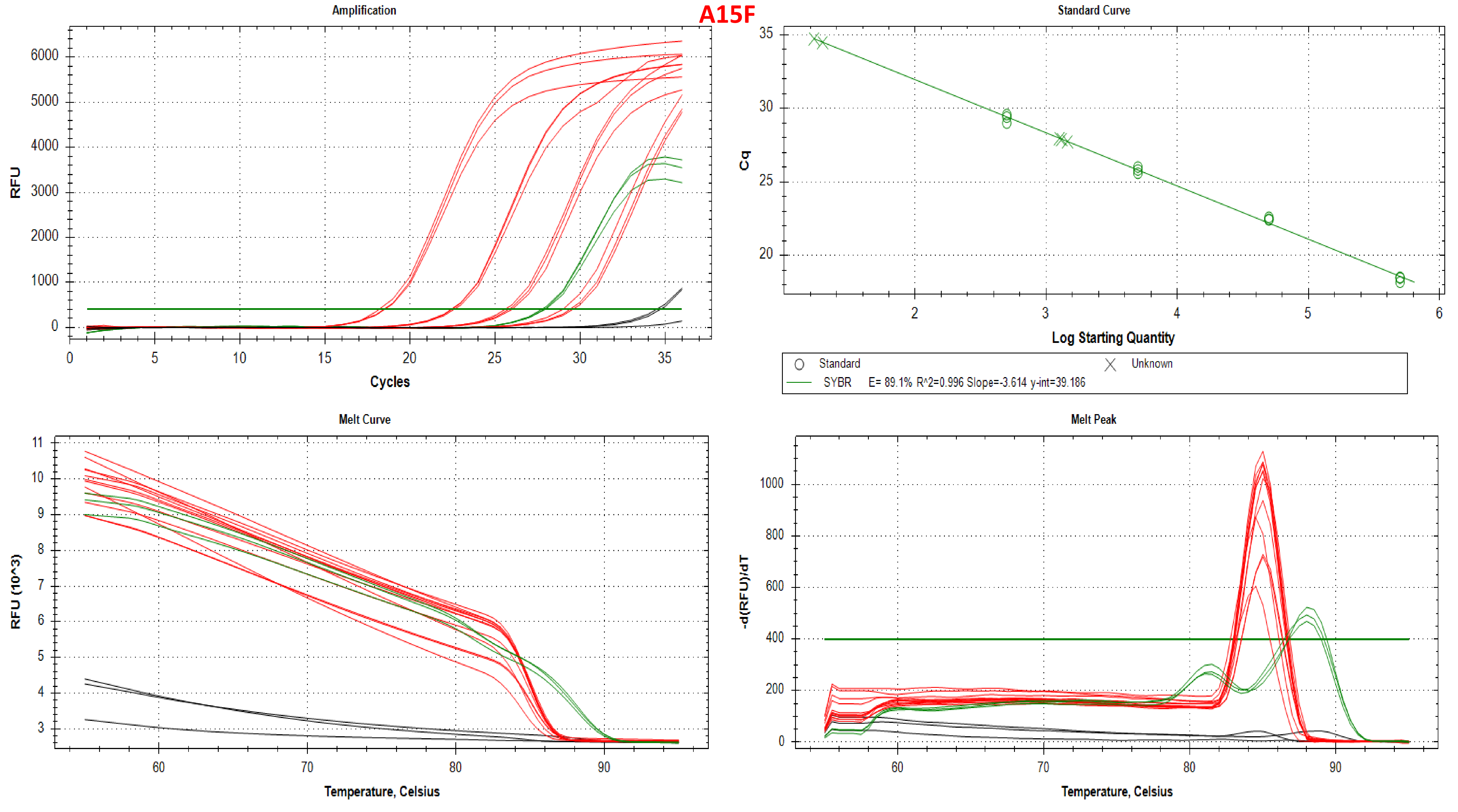

A15F
Amplification
RFU
6000
5000
4000
3000
2000
1000
0
0
5
10
15
20
25
30
35
Cycles
Standard Curve
Cq
35
30
25
20
2
3
4
5
6
Log Starting Quantity
Standard
Unknown
SYBR E= 89.1% R^2=0.996 Slope=-3.614 y-int=39.186
Melt Curve
RFU (10^3)
11
10
9
8
7
6
5
4
3
60
70
80
90
Temperature, Celsius
Melt Peak
-d(RFU)/dT
1000
800
600
400
200
0
60
70
80
90
Temperature, Celsius

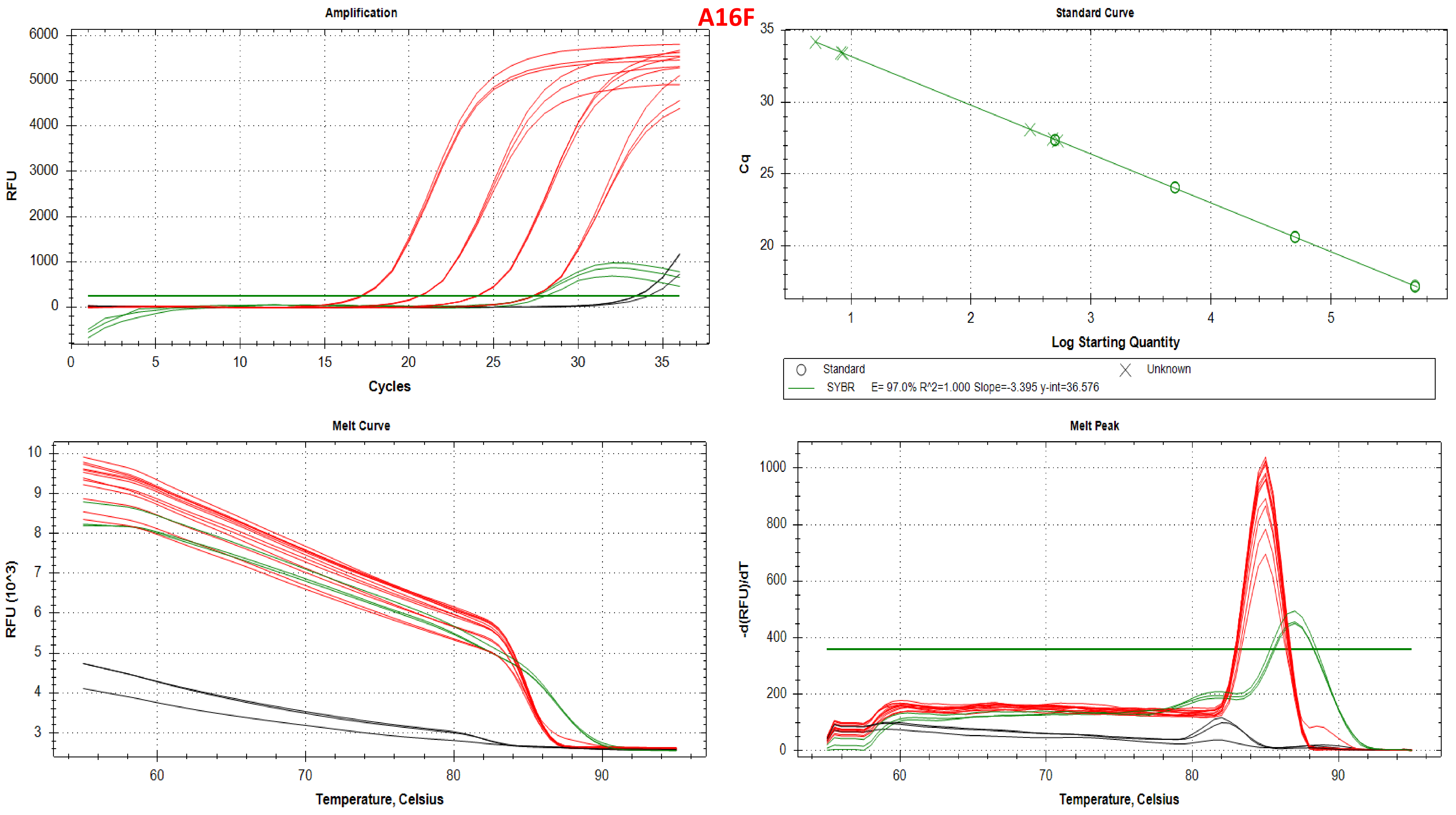
A16F
Amplification
RFU
Cycles
Standard Curve
Cq
Log Starting Quantity
Standard
Unknown
SYBR E= 97.0% R^2=1.000 Slope=-3.395 y-int=36.576
Melt Curve
RFU (10^3)
Temperature, Celsius
Melt Peak
-d(RFU)/dT
Temperature, Celsius

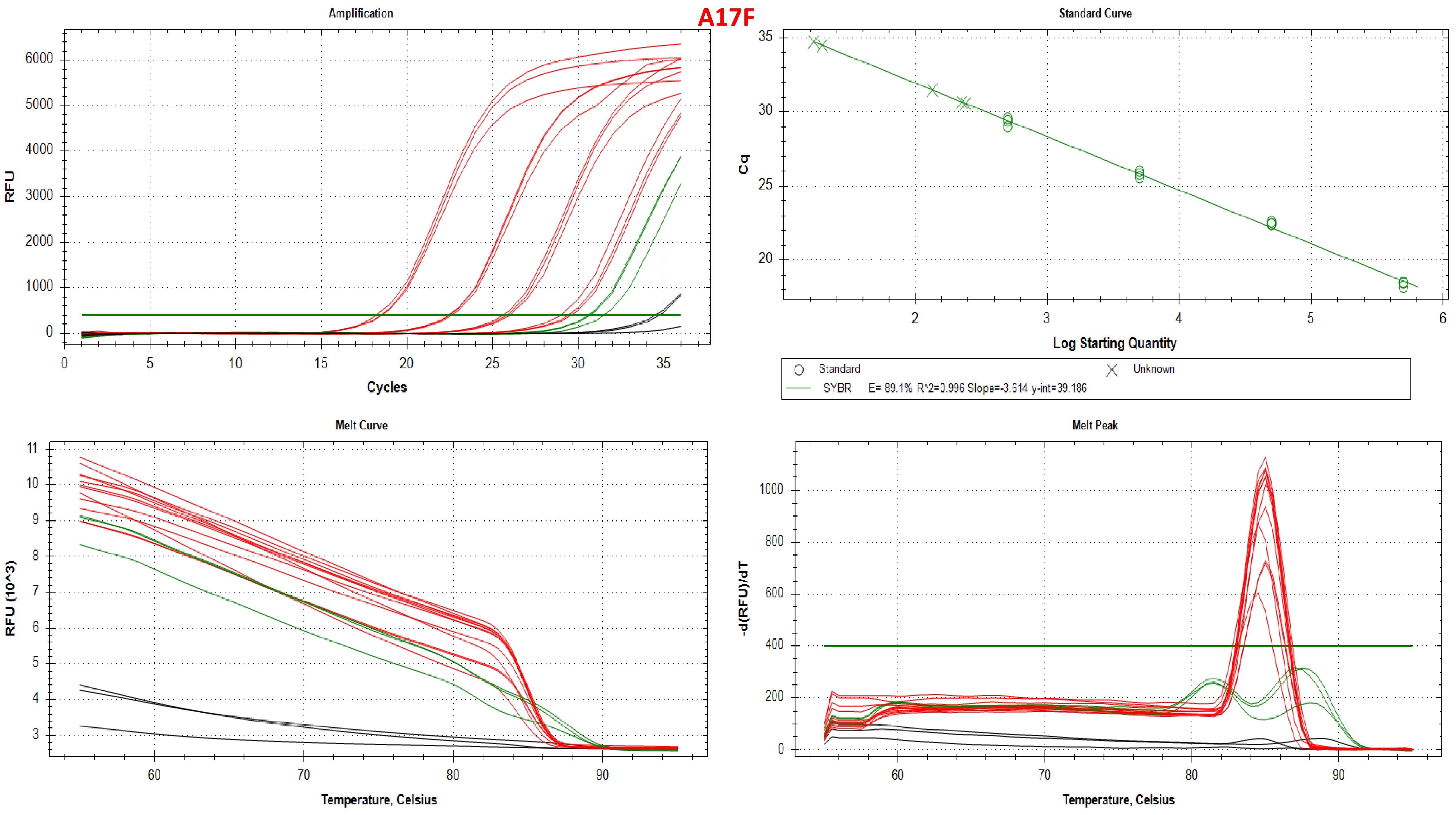
A17F
Amplification
RFU
Cycles
Standard Curve
Cq
Log Starting Quantity
Standard
Unknown
SYBR E= 89.1% R^2=0.996 Slope=-3.614 y-int=39.186
Melt Curve
RFU (10^3)
Temperature, Celsius
Melt Peak
-d(RFU)/dT
Temperature, Celsius

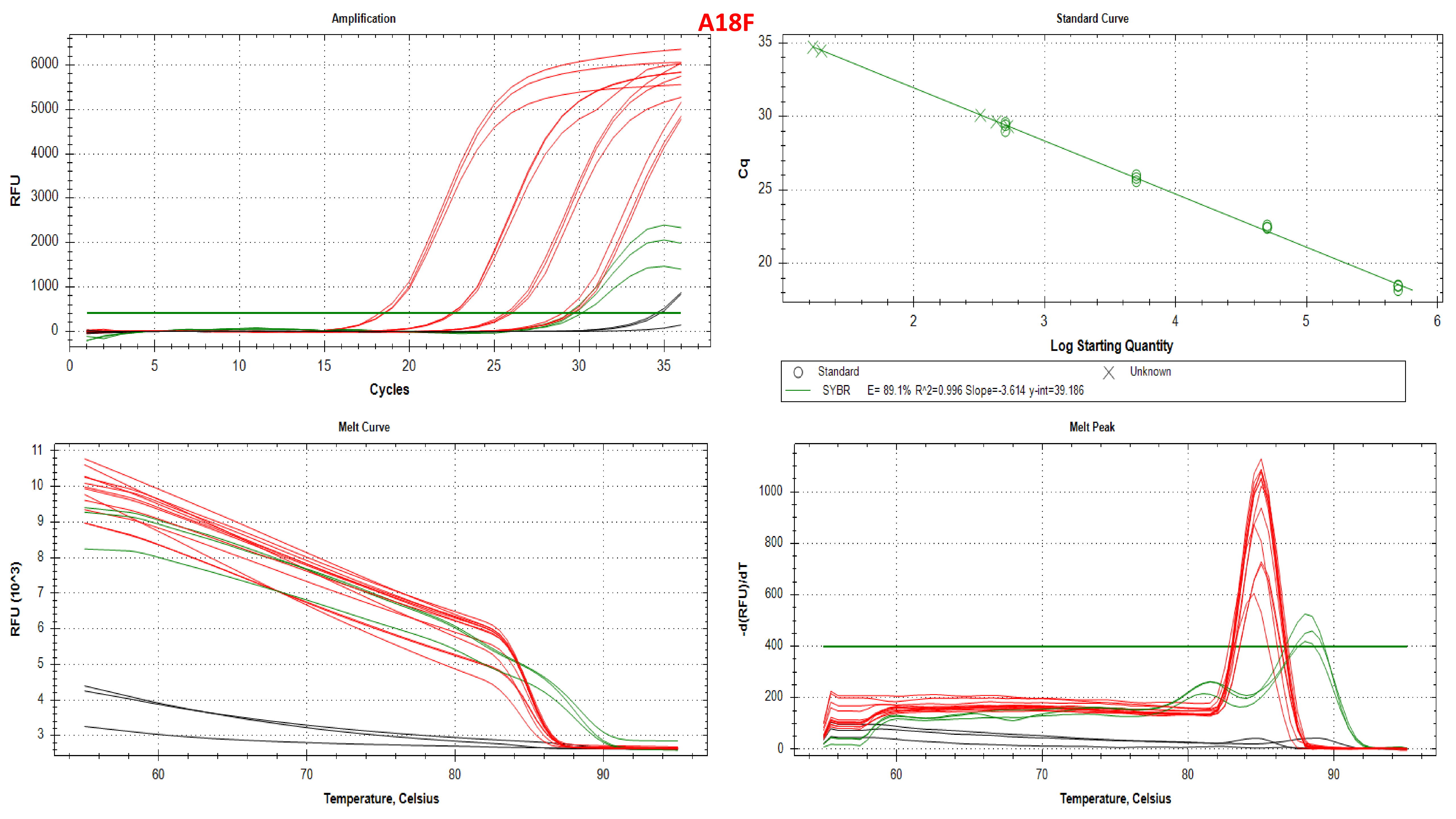

A18F
Amplification
RFU
Cycles
Standard Curve
Cq
Log Starting Quantity
Standard
Unknown
SYBR E= 89.1% R^2=0.996 Slope=-3.614 y-int=39.186
Melt Curve
RFU (10^3)
Temperature, Celsius
Melt Peak
-d(RFU)/dT
Temperature, Celsius

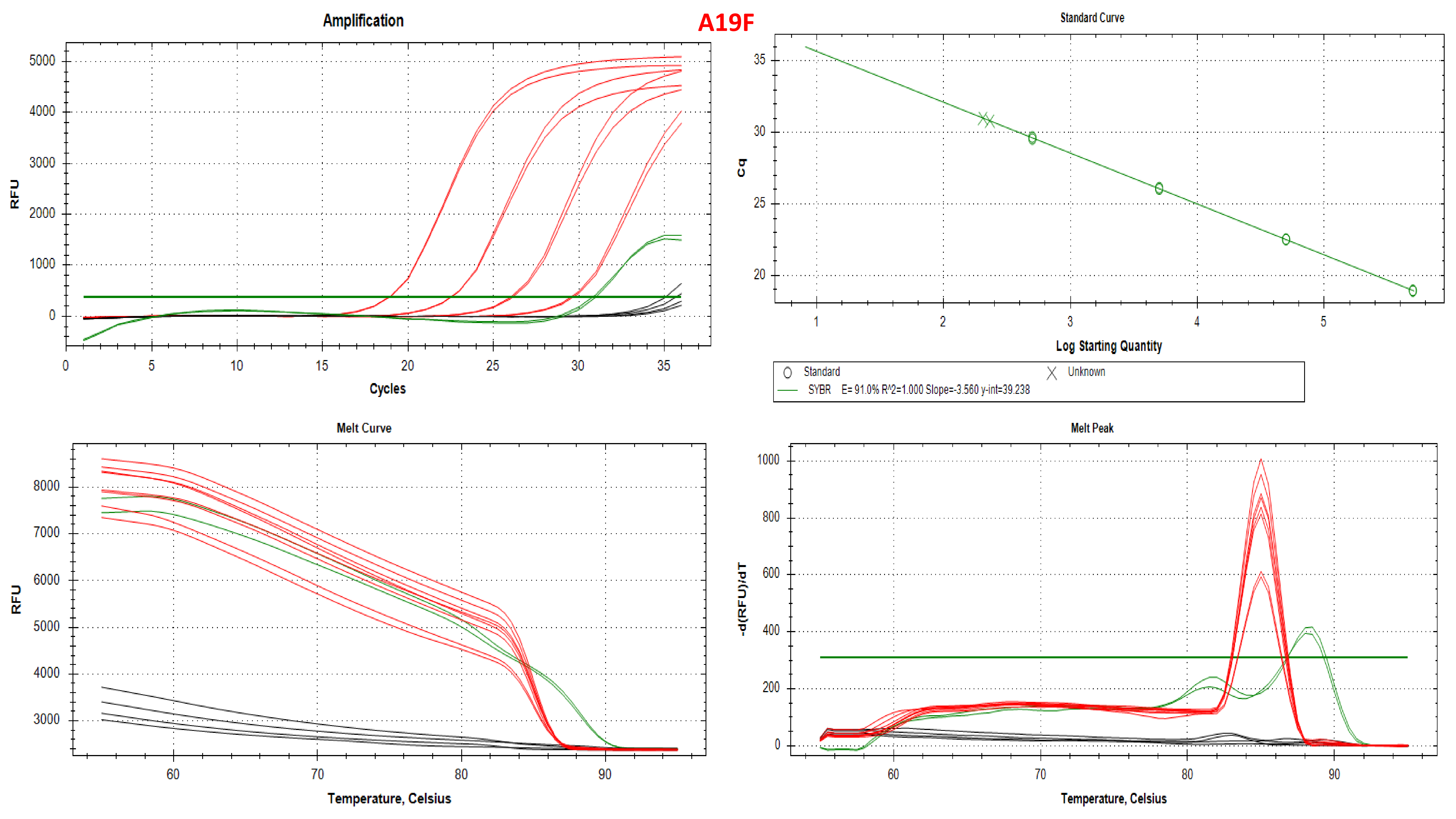
A19F
Amplification
RFU
Cycles
Standard Curve
Cq
Log Starting Quantity
Standard
Unknown
SYBR E= 91.0% R^2=1.000 Slope=-3.560 y-int=39.238
Melt Curve
RFU
Temperature, Celsius
Melt Peak
-d(RFU)/dT
Temperature, Celsius

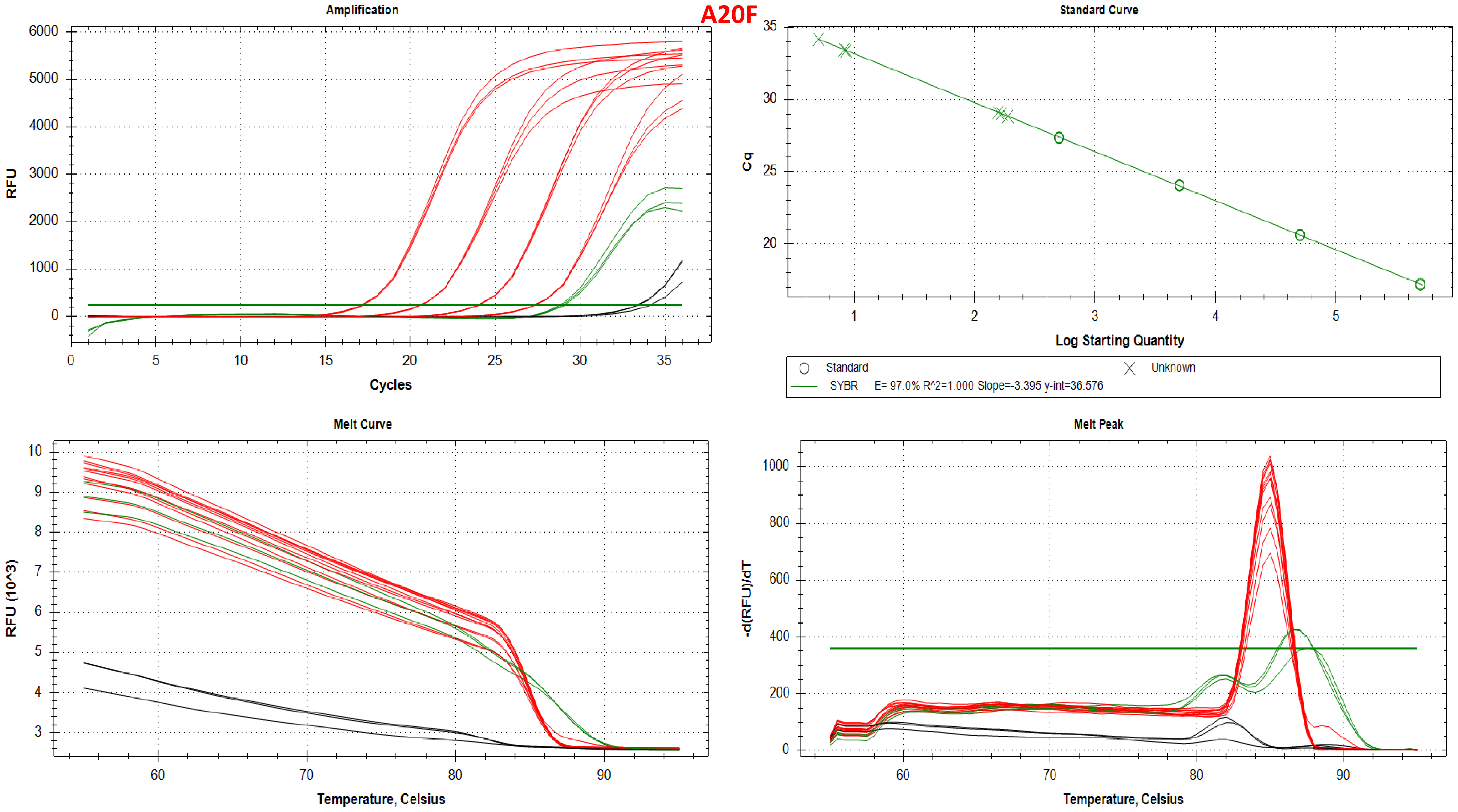
A20F
Amplification
RFU
Cycles
Standard Curve
Cq
Log Starting Quantity
Standard
Unknown
SYBR E= 97.0% R^2=1.000 Slope=-3.395 y-int=36.576
Melt Curve
RFU (10^3)
Temperature, Celsius
Melt Peak
-d(RFU)/dT
Temperature, Celsius

A21F

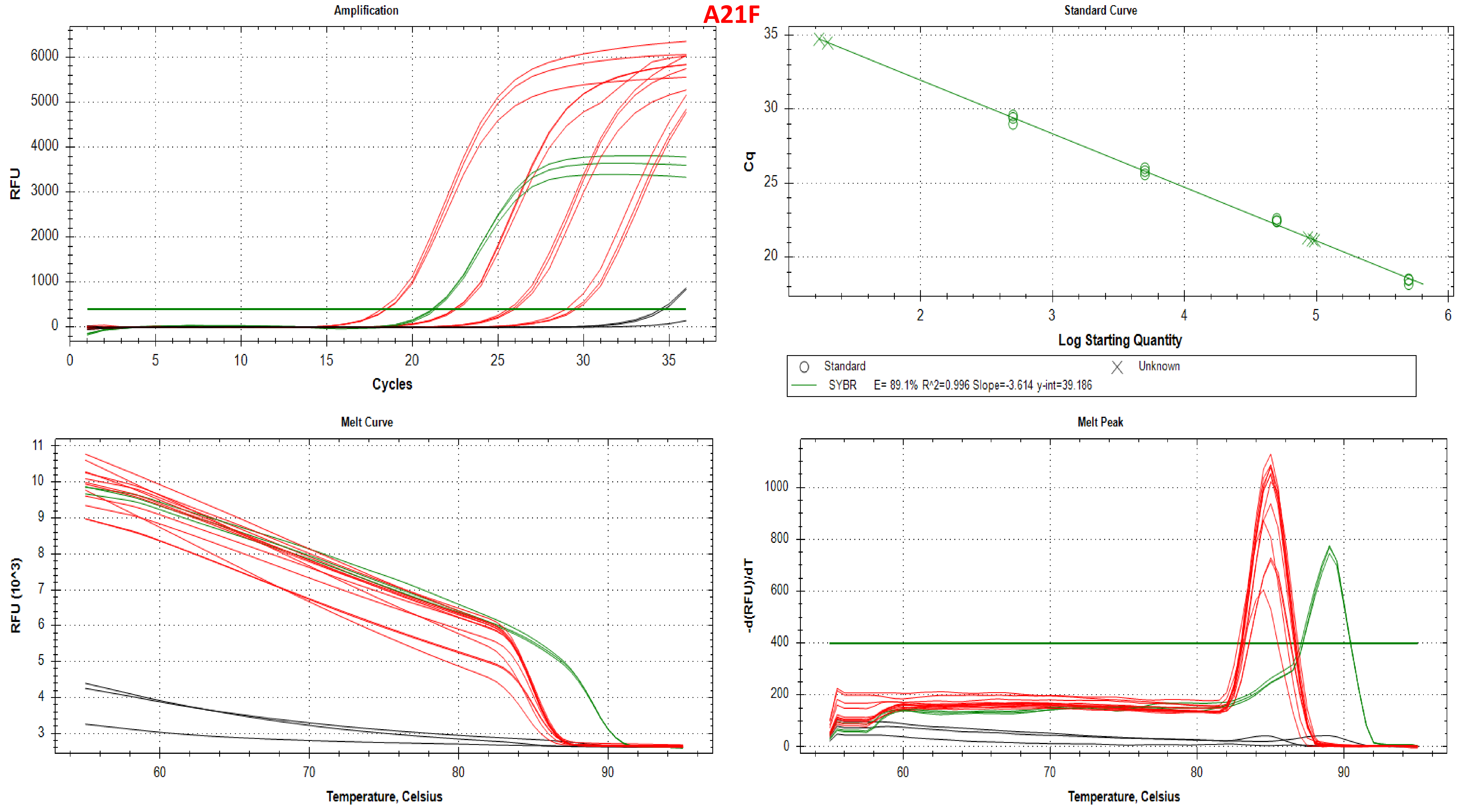
Amplification
RFU
Cycles
Standard Curve
Cq
Log Starting Quantity
Standard
Unknown
SYBR E= 89.1% R^2=0.996 Slope=-3.614 y-int=39.186
Melt Curve
RFU (10^3)
Temperature, Celsius
Melt Peak
-d(RFU)/dT
Temperature, Celsius

A22F

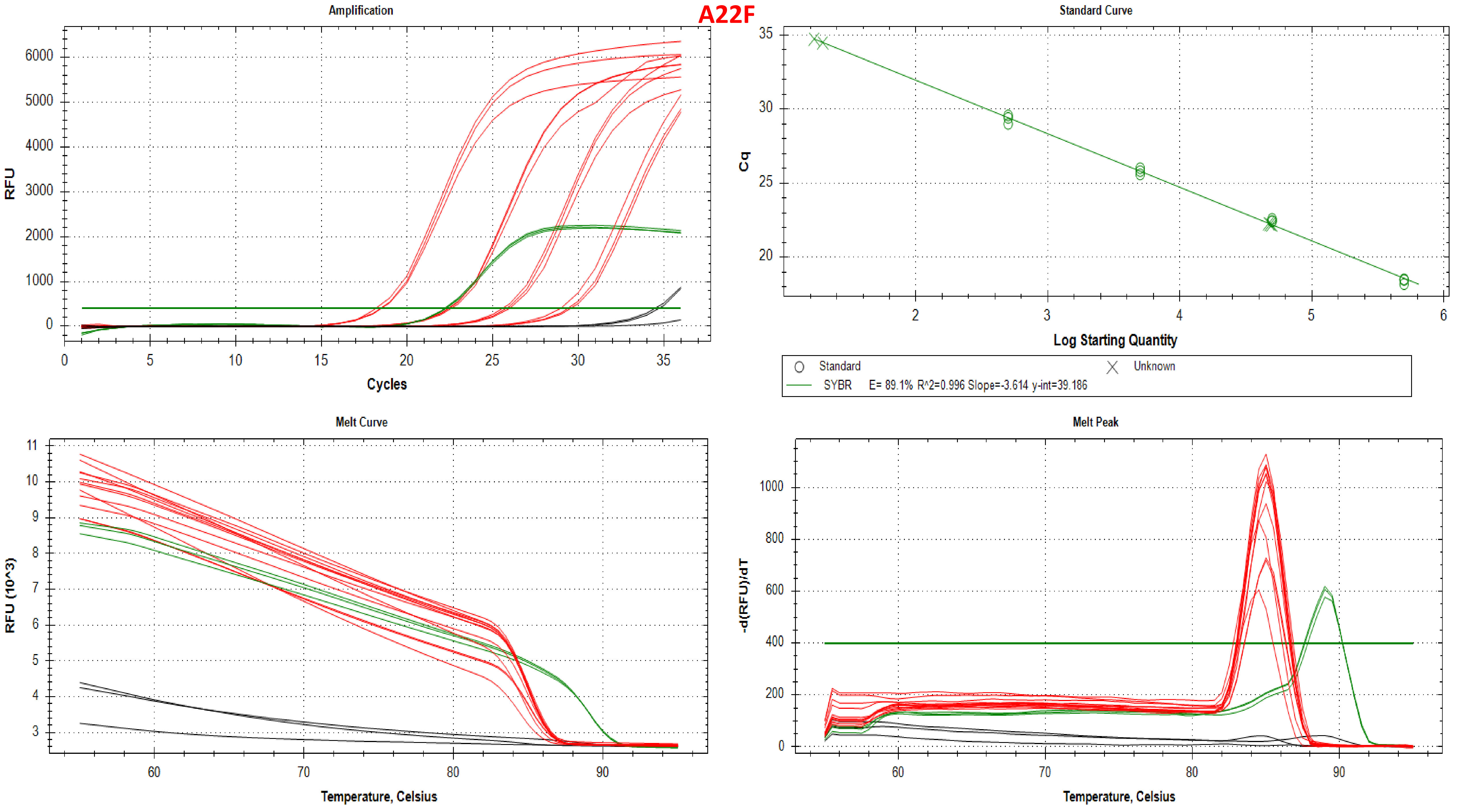
Amplification
RFU
Cycles
Standard Curve
Cq
Log Starting Quantity
Standard
Unknown
SYBR E= 89.1% R^2=0.996 Slope=-3.614 y-int=39.186
Melt Curve
RFU (10^3)
Temperature, Celsius
Melt Peak
-d(RFU)/dT
Temperature, Celsius

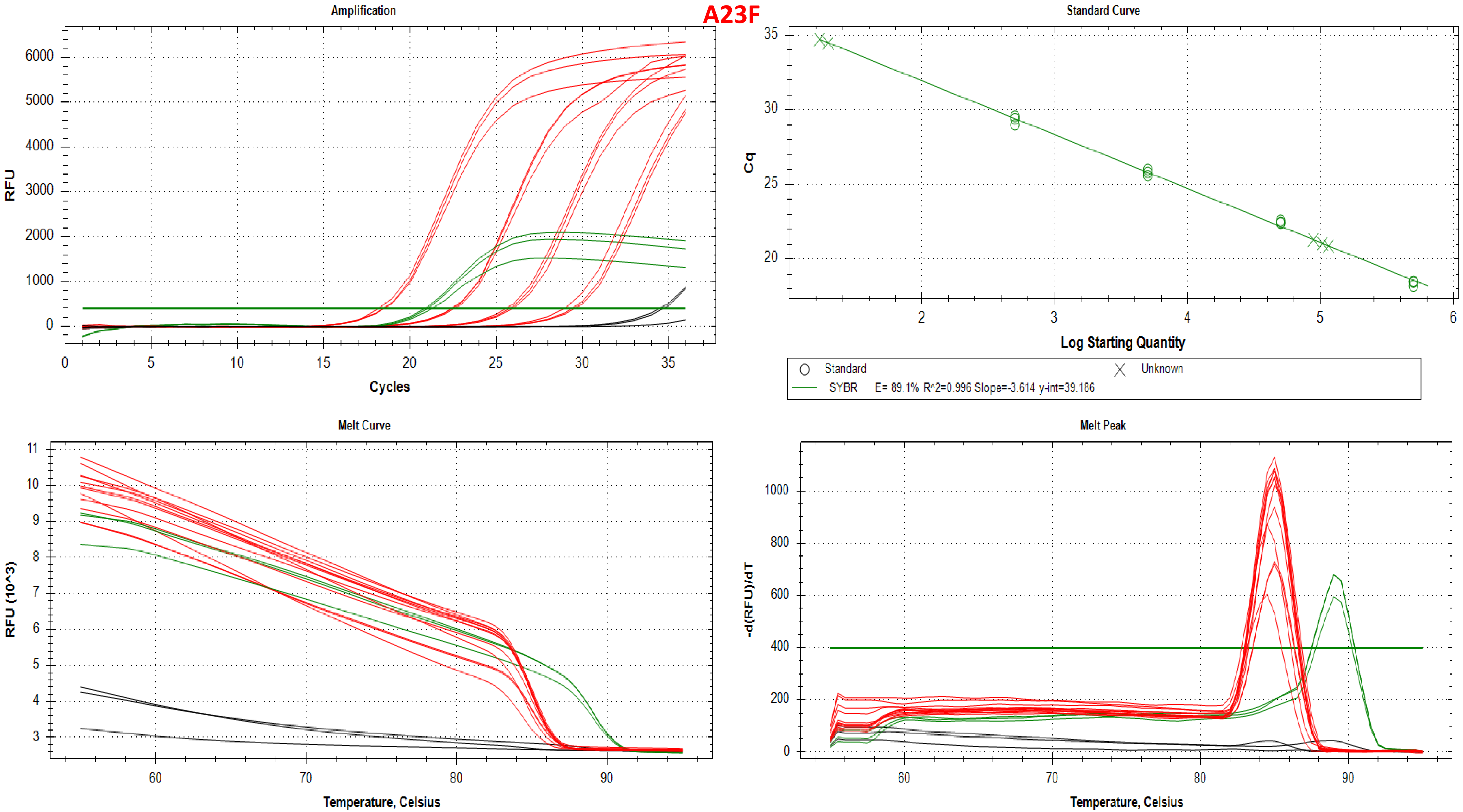

A23F
Amplification
RFU
Cycles
Standard Curve
Cq
Log Starting Quantity
Standard
Unknown
SYBR E= 89.1% R^2=0.996 Slope=-3.614 y-int=39.186
Melt Curve
RFU (10^3)
Temperature, Celsius
Melt Peak
-d(RFU)/dT
Temperature, Celsius

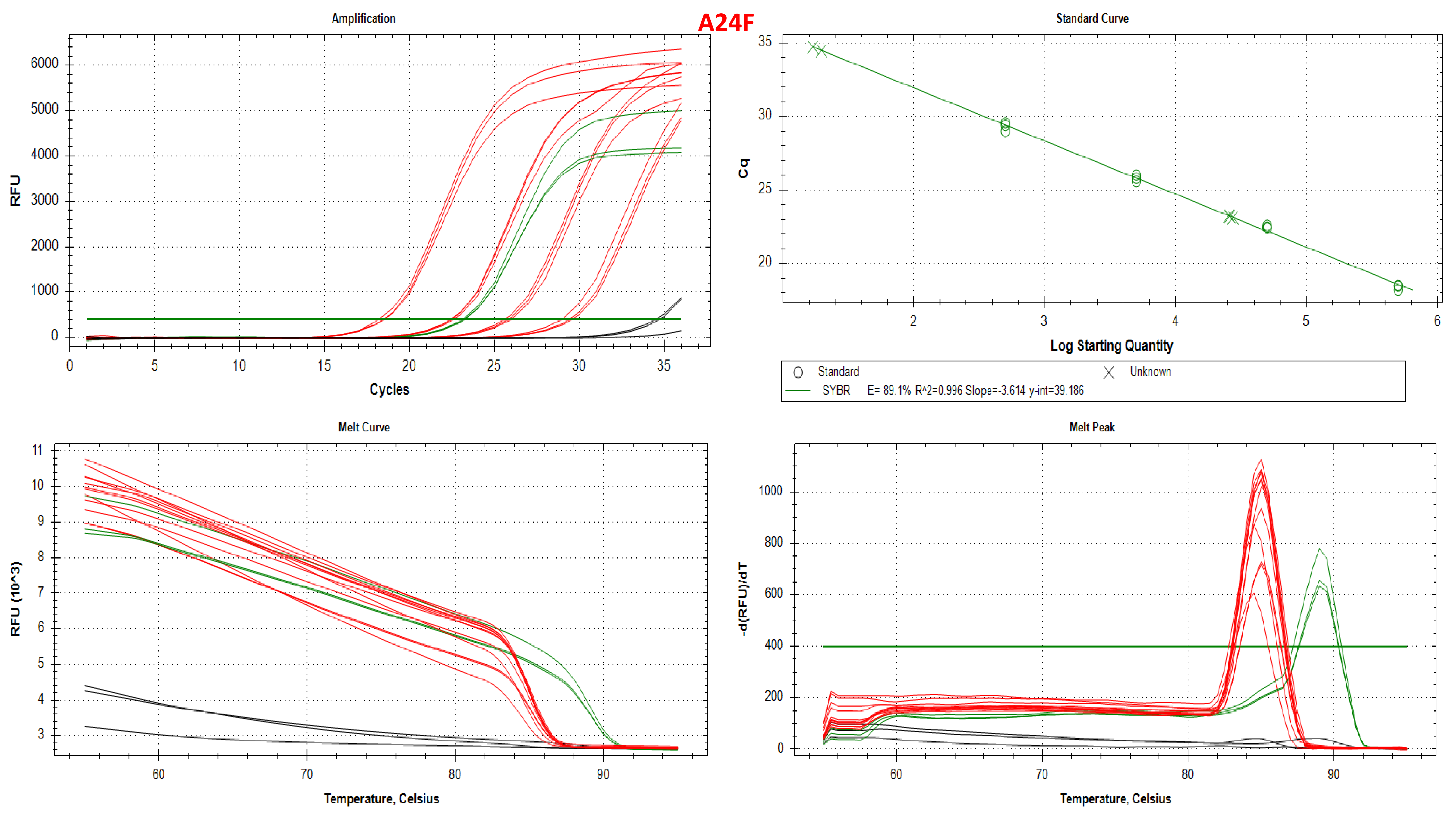
A24F
Amplification
RFU
Cycles
Standard Curve
Cq
Log Starting Quantity
Standard
Unknown
SYBR E= 89.1% R^2=0.996 Slope=-3.614 y-int=39.186
Melt Curve
RFU (10^3)
Temperature, Celsius
Melt Peak
-d(RFU)/dT
Temperature, Celsius

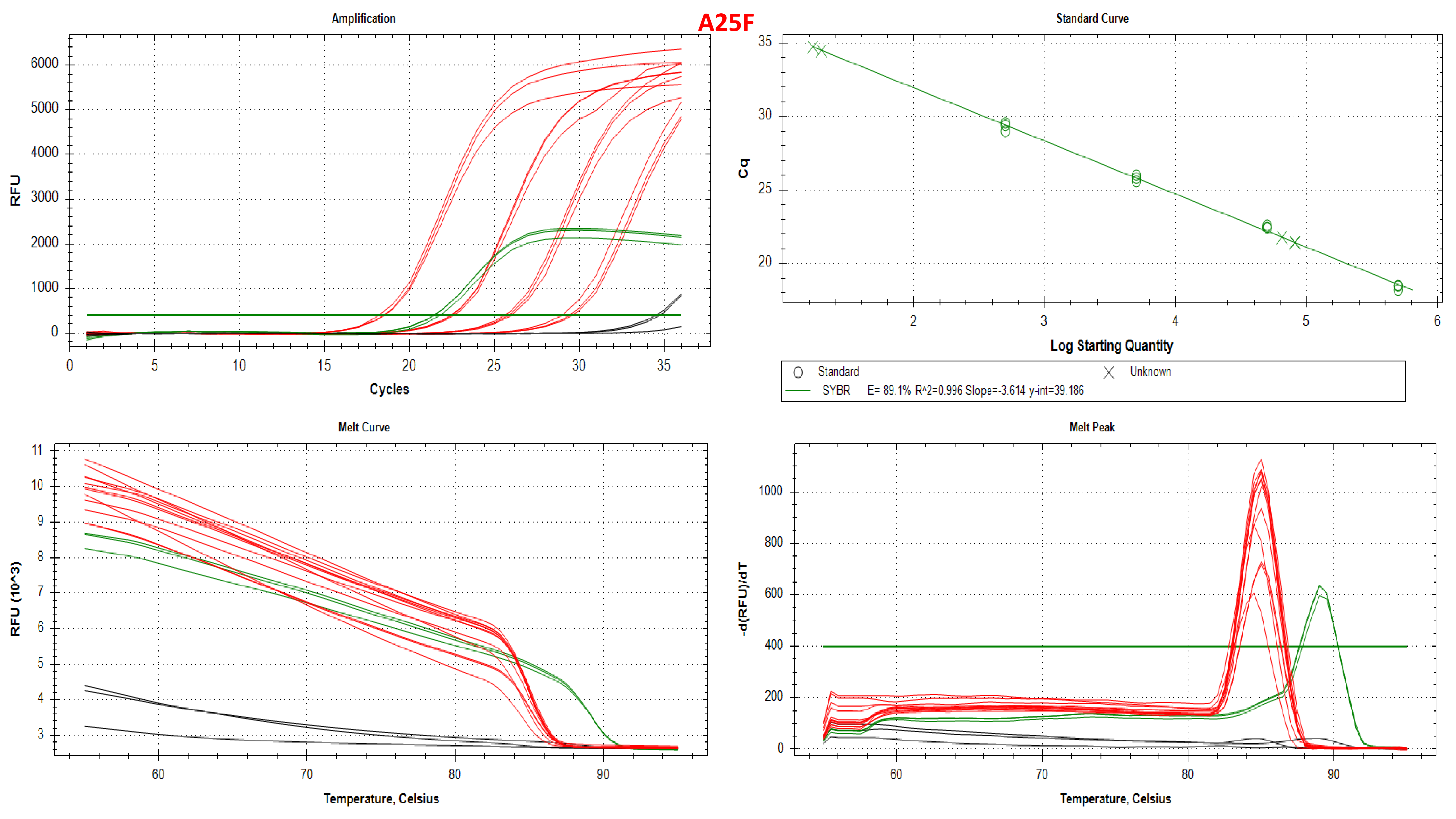

A25F
Amplification
RFU
Cycles
Standard Curve
Cq
Log Starting Quantity
Standard
Unknown
SYBR E= 89.1% R^2=0.996 Slope=-3.614 y-int=39.186
Melt Curve
RFU (10^3)
Temperature, Celsius
Melt Peak
-d(RFU)/dT
Temperature, Celsius

A26F

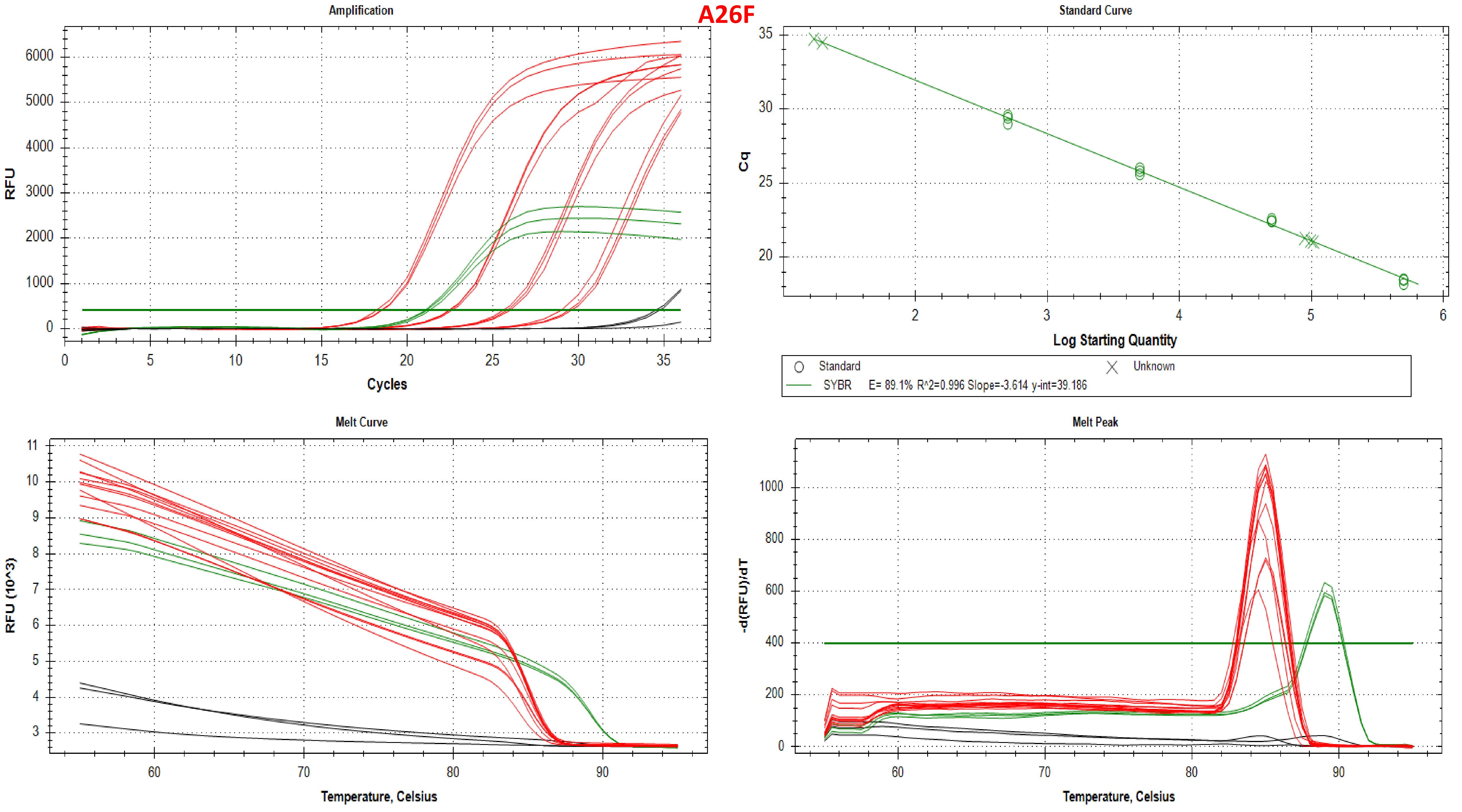

Amplification
RFU
Cycles
Standard Curve
Cq
Log Starting Quantity
Standard
Unknown
SYBR E= 89.1% R^2=0.996 Slope=-3.614 y-int=39.186
Melt Curve
RFU (10^3)
Temperature, Celsius
Melt Peak
-d(RFU)/dT
Temperature, Celsius

A27F

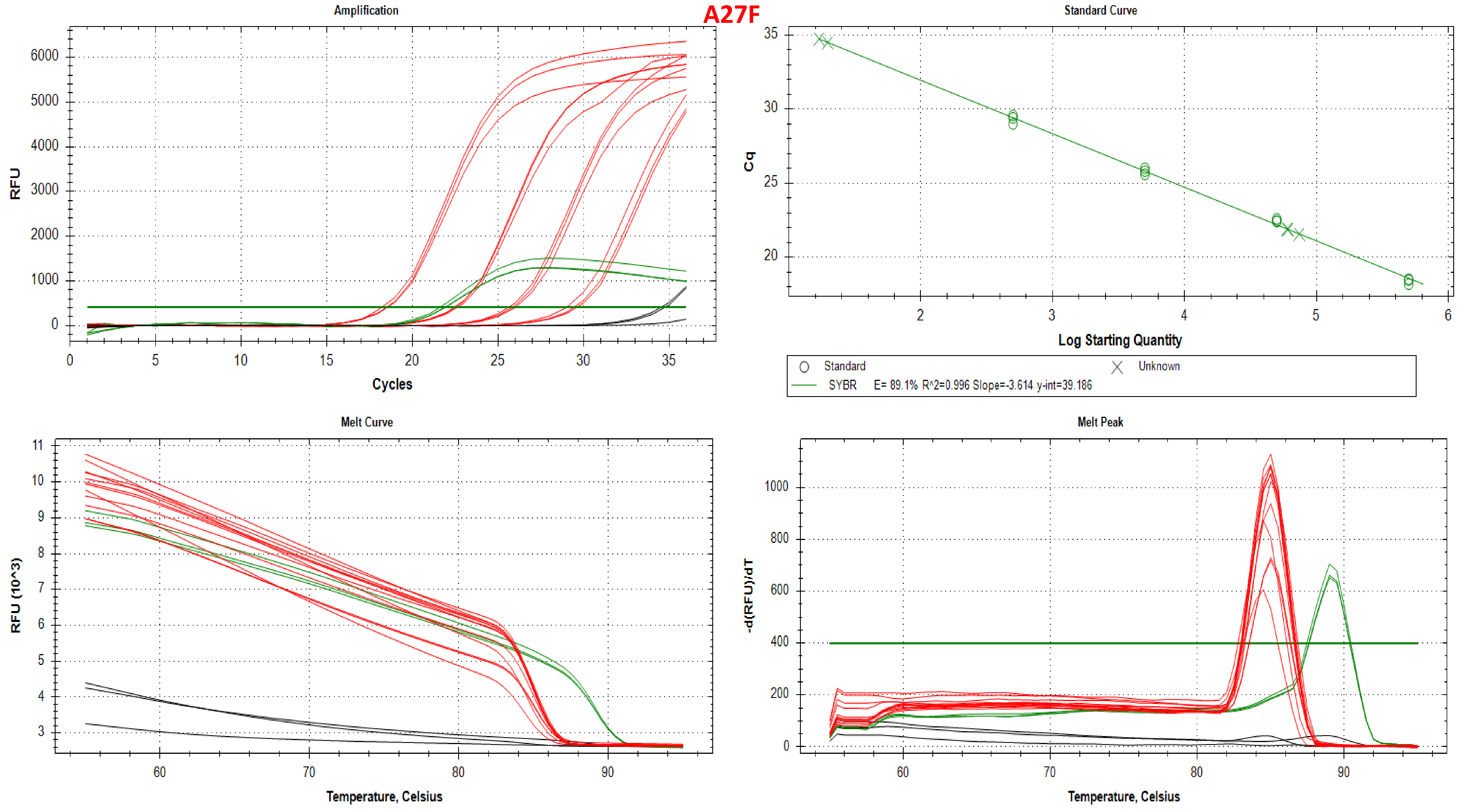

Amplification
RFU
Cycles
Standard Curve
Cq
Log Starting Quantity
Standard
Unknown
SYBR E= 89.1% R^2=0.996 Slope=-3.614 y-int=39.186
Melt Curve
RFU (10^3)
Temperature, Celsius
Melt Peak
-d(RFU)/dT
Temperature, Celsius

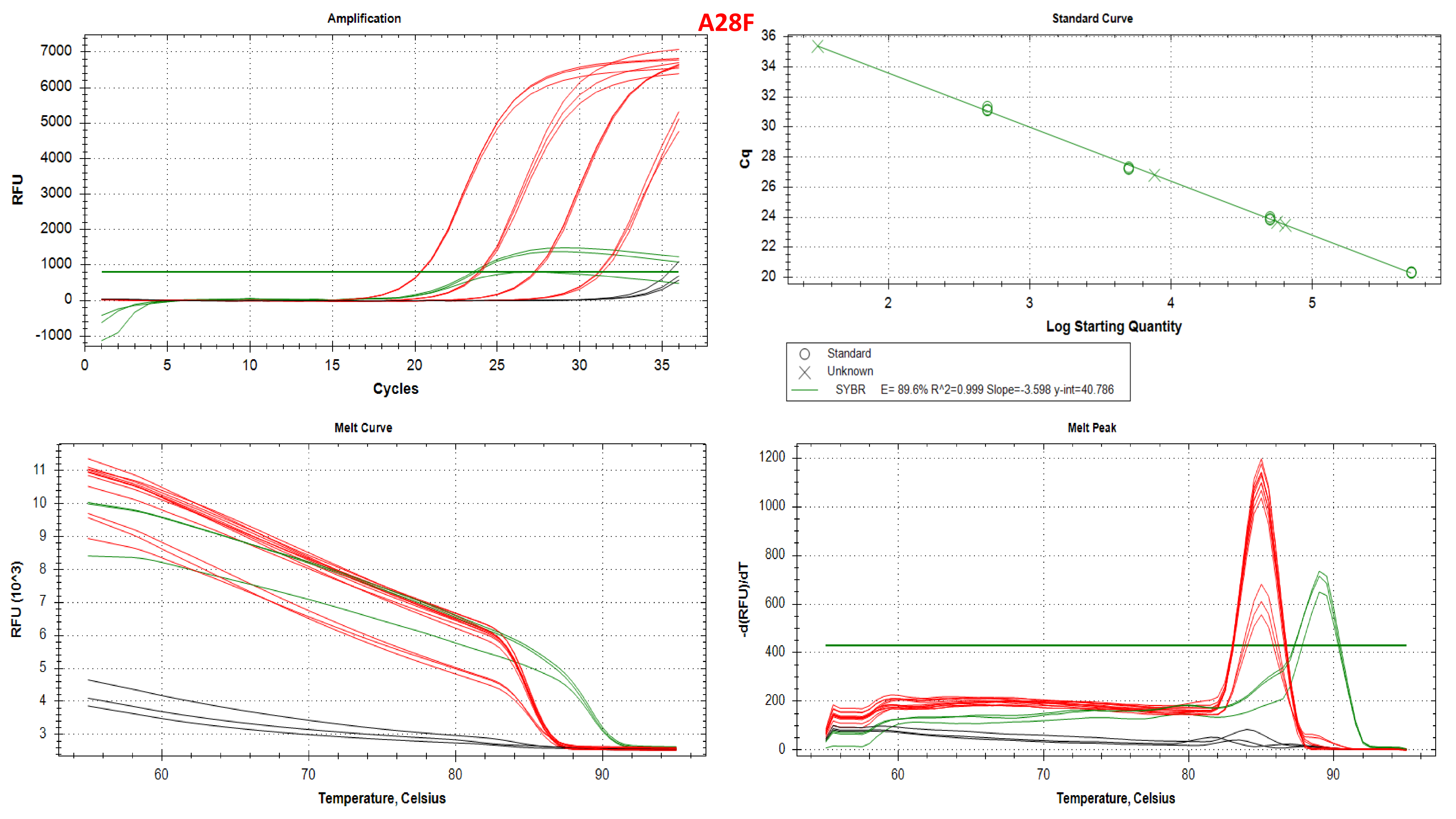

A28F
Amplification
RFU
Cycles
Standard Curve
Cq
Log Starting Quantity
Standard
Unknown
SYBR E= 89.6% R^2=0.999 Slope=-3.598 y-int=40.786
Melt Curve
RFU (10^3)
Temperature, Celsius
Melt Peak
-d(RFU)/dT
Temperature, Celsius

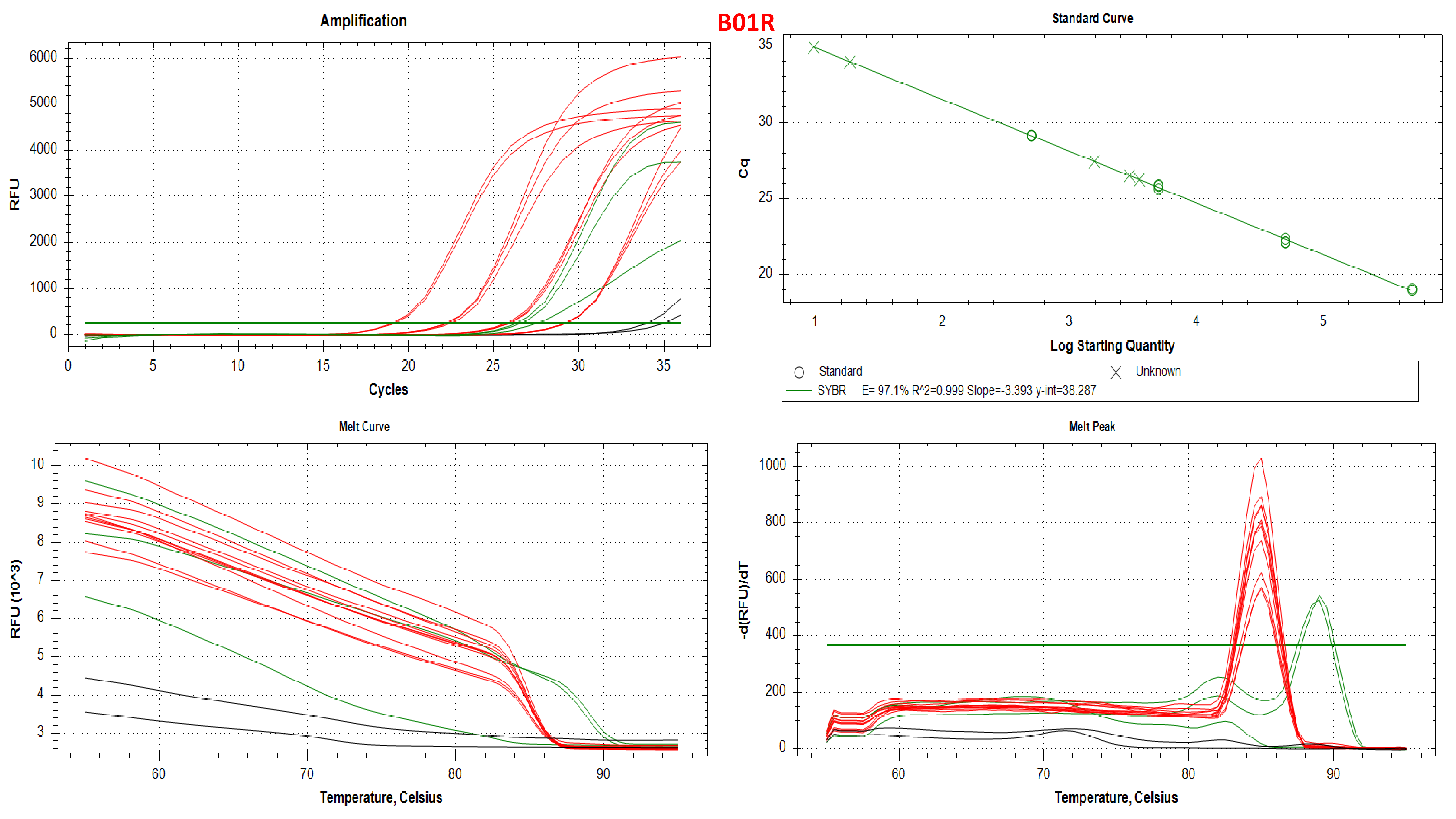
B01R
Amplification
RFU
Cycles
Standard Curve
Cq
Log Starting Quantity
Standard
Unknown
SYBR E= 97.1% R^2=0.999 Slope=-3.393 y-int=38.287
Melt Curve
RFU (10^3)
Temperature, Celsius
Melt Peak
-d(RFU)/dT
Temperature, Celsius

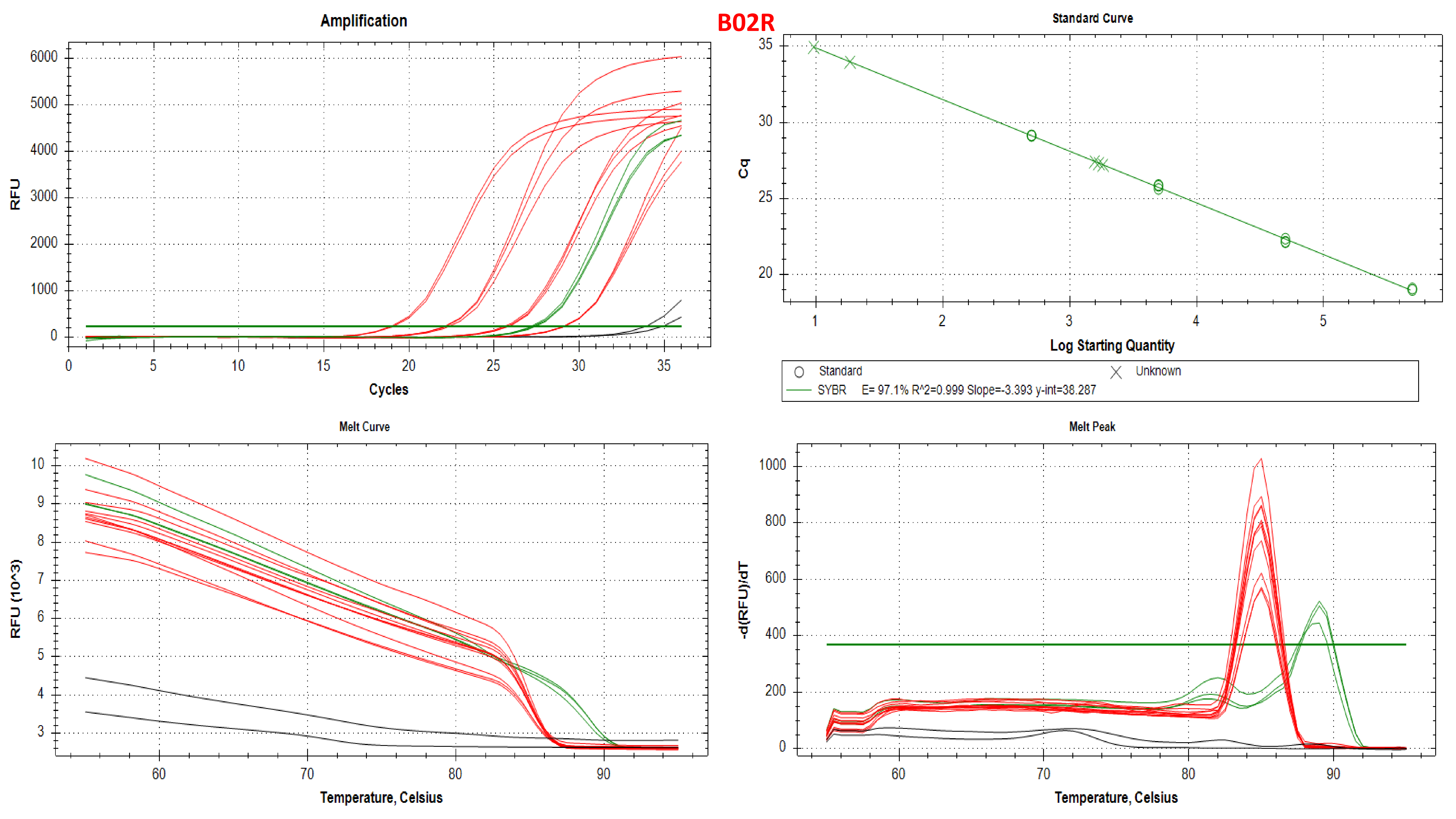
B02R
Amplification
RFU
Cycles
Standard Curve
Cq
Log Starting Quantity
Standard
Unknown
SYBR E= 97.1% R^2=0.999 Slope=-3.393 y-int=38.287
Melt Curve
RFU (10^3)
Temperature, Celsius
Melt Peak
-d(RFU)/dT
Temperature, Celsius

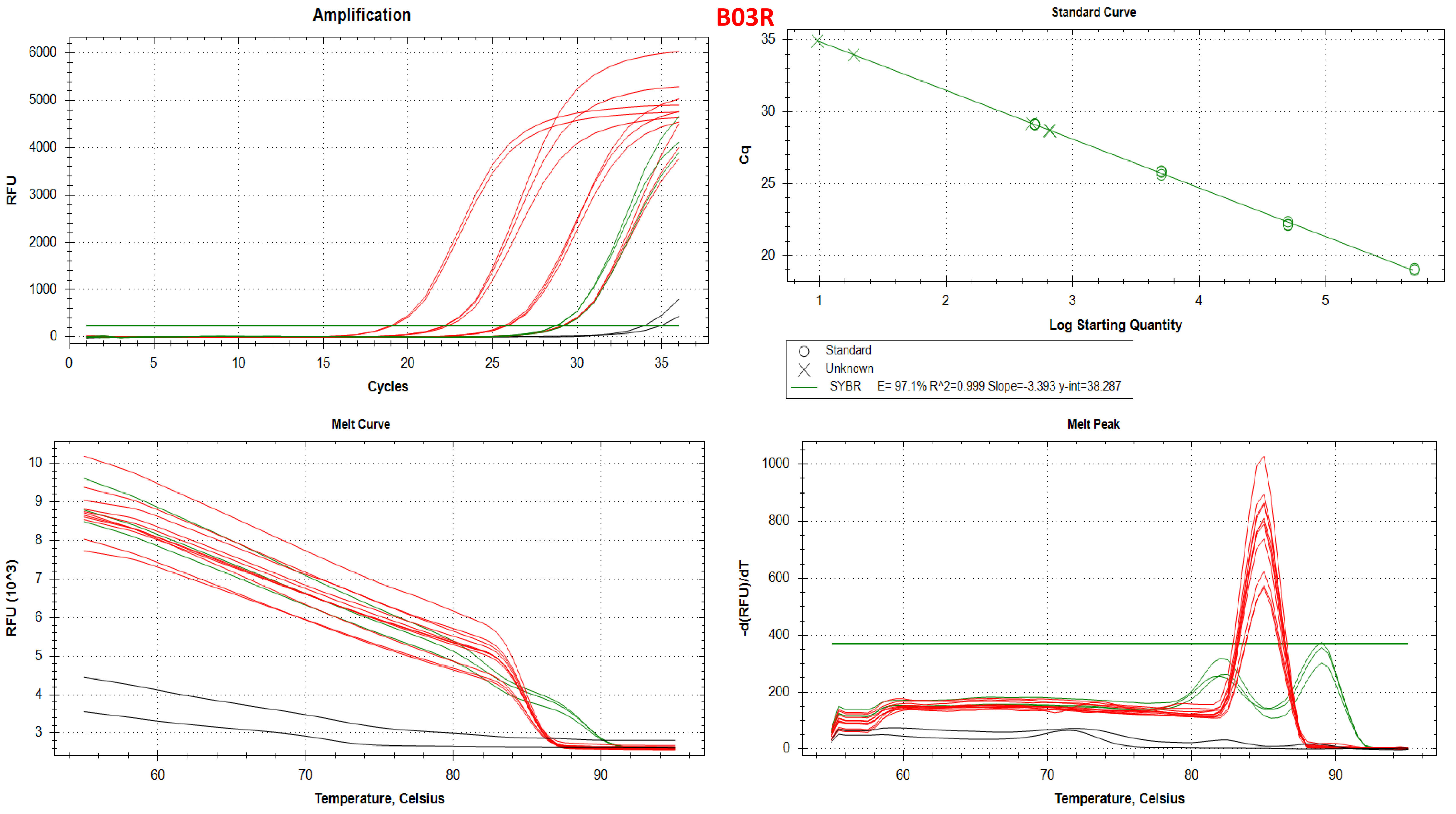
B03R
Amplification
RFU
Cycles
Standard Curve
Cq
Log Starting Quantity
Standard
Unknown
SYBR E= 97.1% R^2=0.999 Slope=-3.393 y-int=38.287
Melt Curve
RFU (10^3)
Temperature, Celsius
Melt Peak
-d(RFU)/dT
Temperature, Celsius

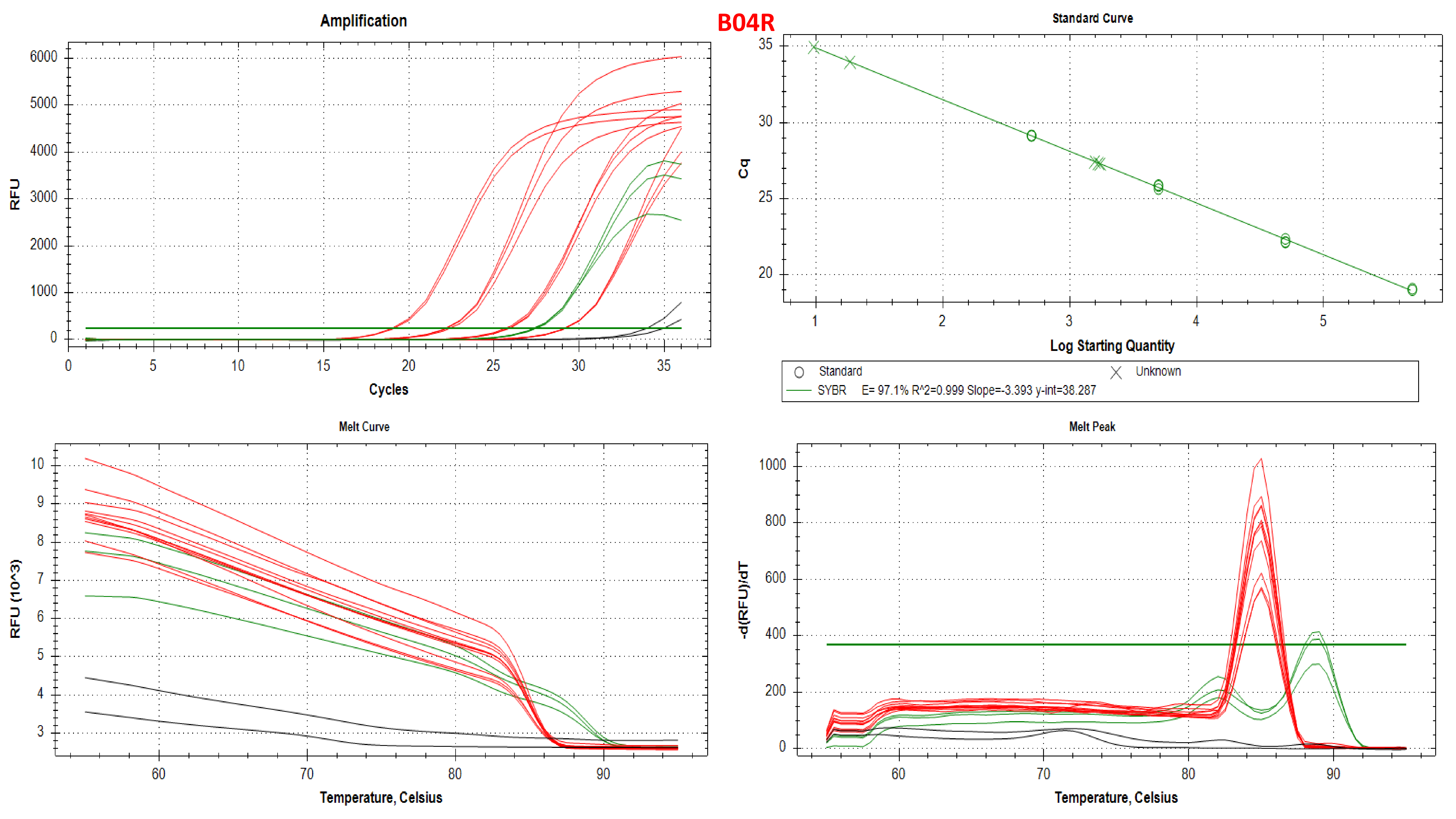
B04R
Amplification
RFU
Cycles
Standard Curve
Cq
Log Starting Quantity
Standard
Unknown
SYBR E= 97.1% R^2=0.999 Slope=-3.393 y-int=38.287
Melt Curve
RFU (10^3)
Temperature, Celsius
Melt Peak
-d(RFU)/dT
Temperature, Celsius

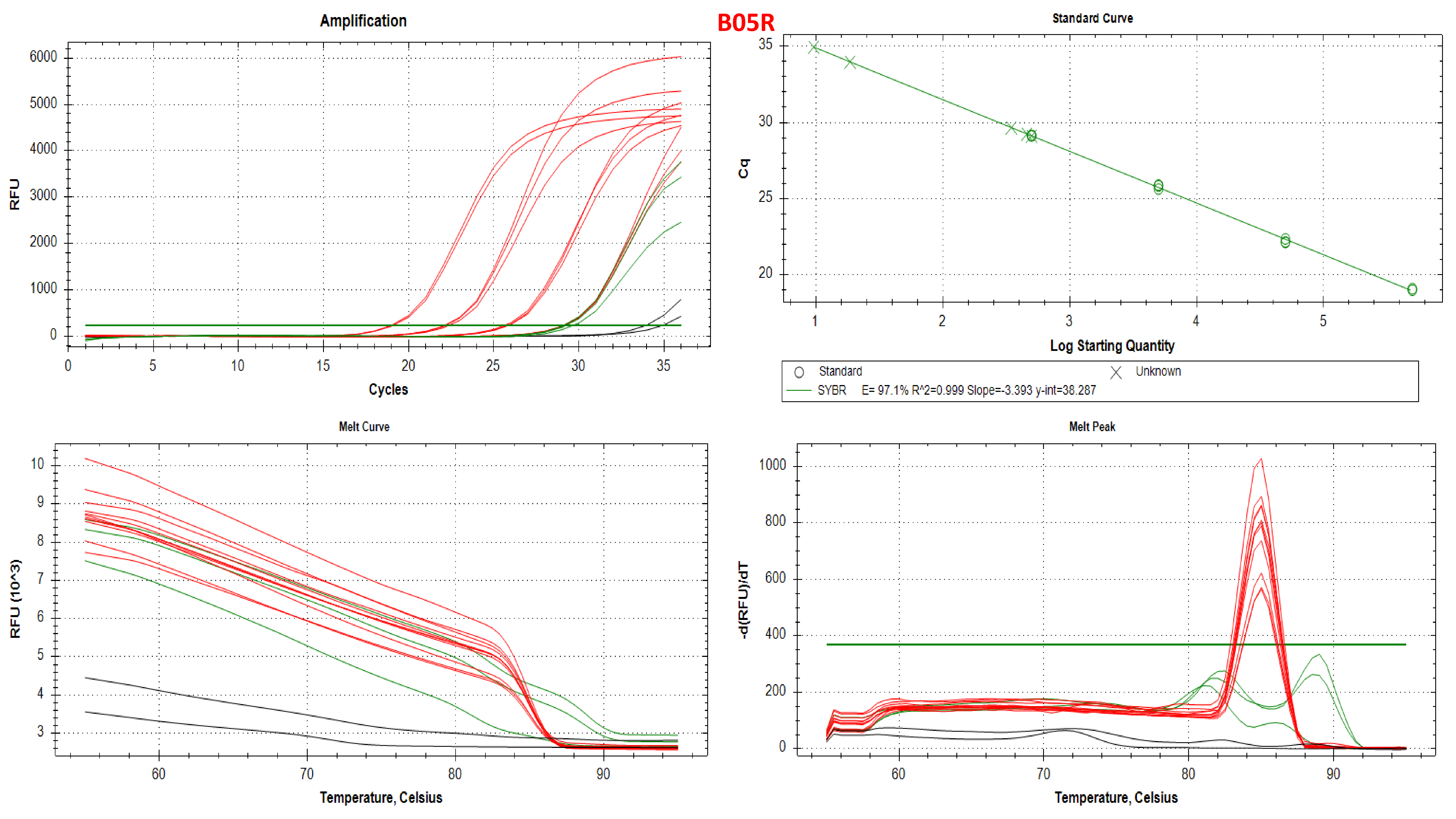
B05R
Amplification
RFU
Cycles
Standard Curve
Cq
Log Starting Quantity
Standard
Unknown
SYBR E= 97.1% R^2=0.999 Slope=-3.393 y-int=38.287
Melt Curve
RFU (10^3)
Temperature, Celsius
Melt Peak
-d(RFU)/dT
Temperature, Celsius

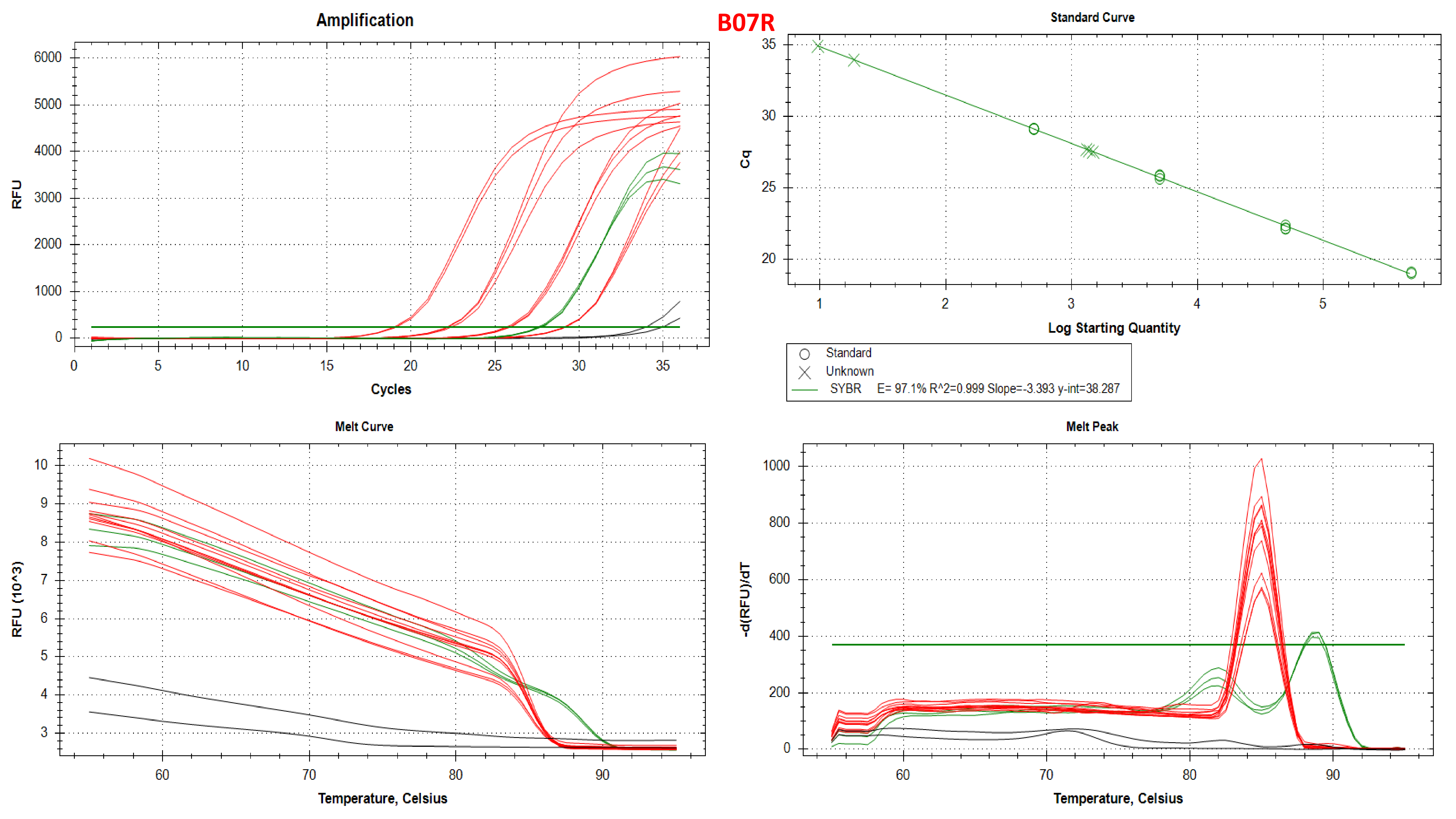
B07R
Amplification
RFU
Cycles
Standard Curve
Cq
Log Starting Quantity
Standard
Unknown
SYBR E= 97.1% R^2=0.999 Slope=-3.393 y-int=38.287
Melt Curve
RFU (10^3)
Temperature, Celsius
Melt Peak
-d(RFU)/dT
Temperature, Celsius

B08R

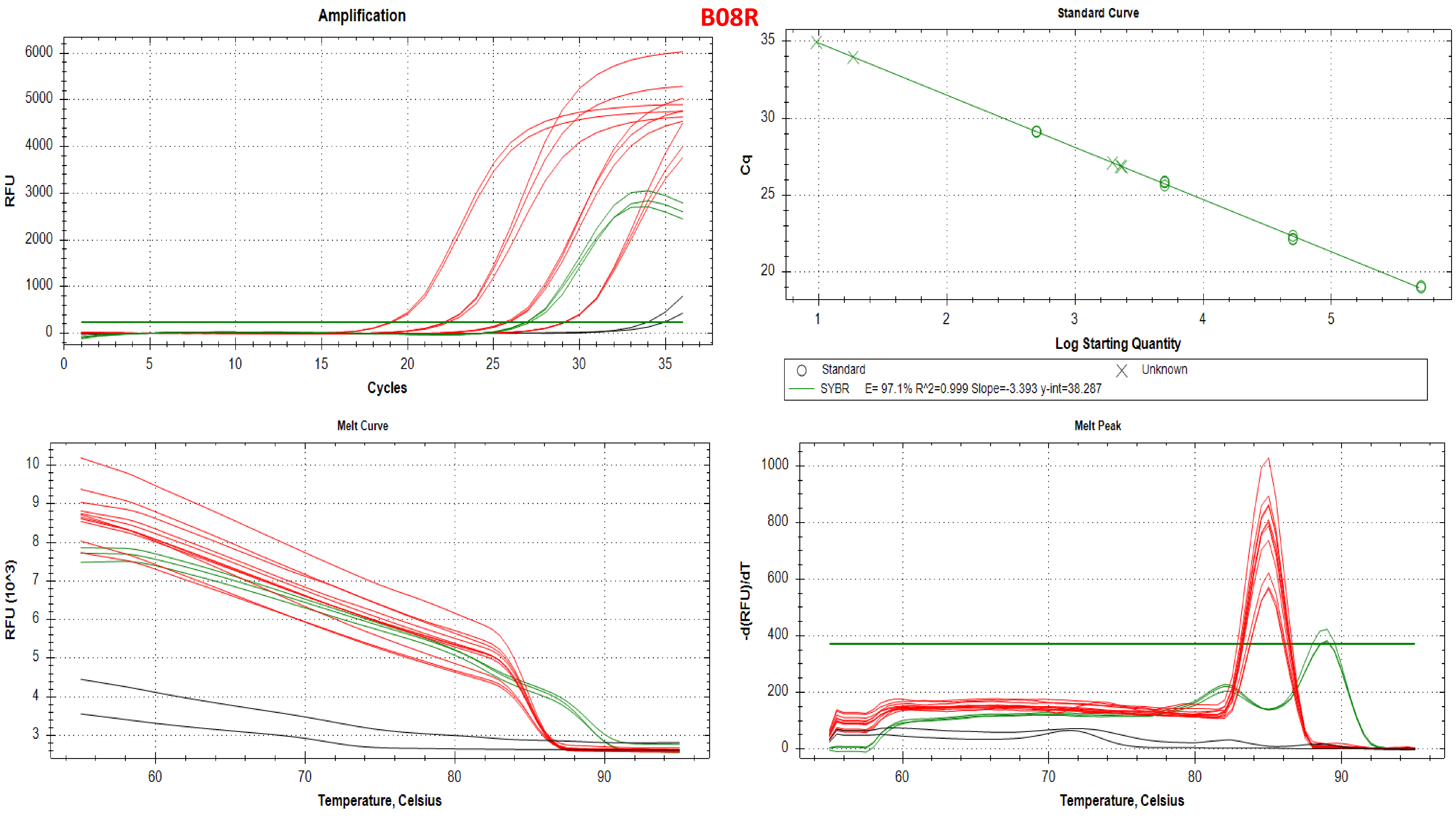

Amplification
RFU
6000
5000
4000
3000
2000
1000
0
0
5
10
15
20
25
30
35
Cycles
Standard Curve
Cq
35
30
25
20
1
2
3
4
5
Log Starting Quantity
Standard
Unknown
SYBR E= 97.1% R^2=0.999 Slope=-3.393 y-int=38.287
Melt Curve
RFU (10^3)
10
9
8
7
6
5
4
3
60
70
80
90
Temperature, Celsius
Melt Peak
-d(RFU)/dT
1000
800
600
400
200
0
60
70
80
90
Temperature, Celsius

B09R

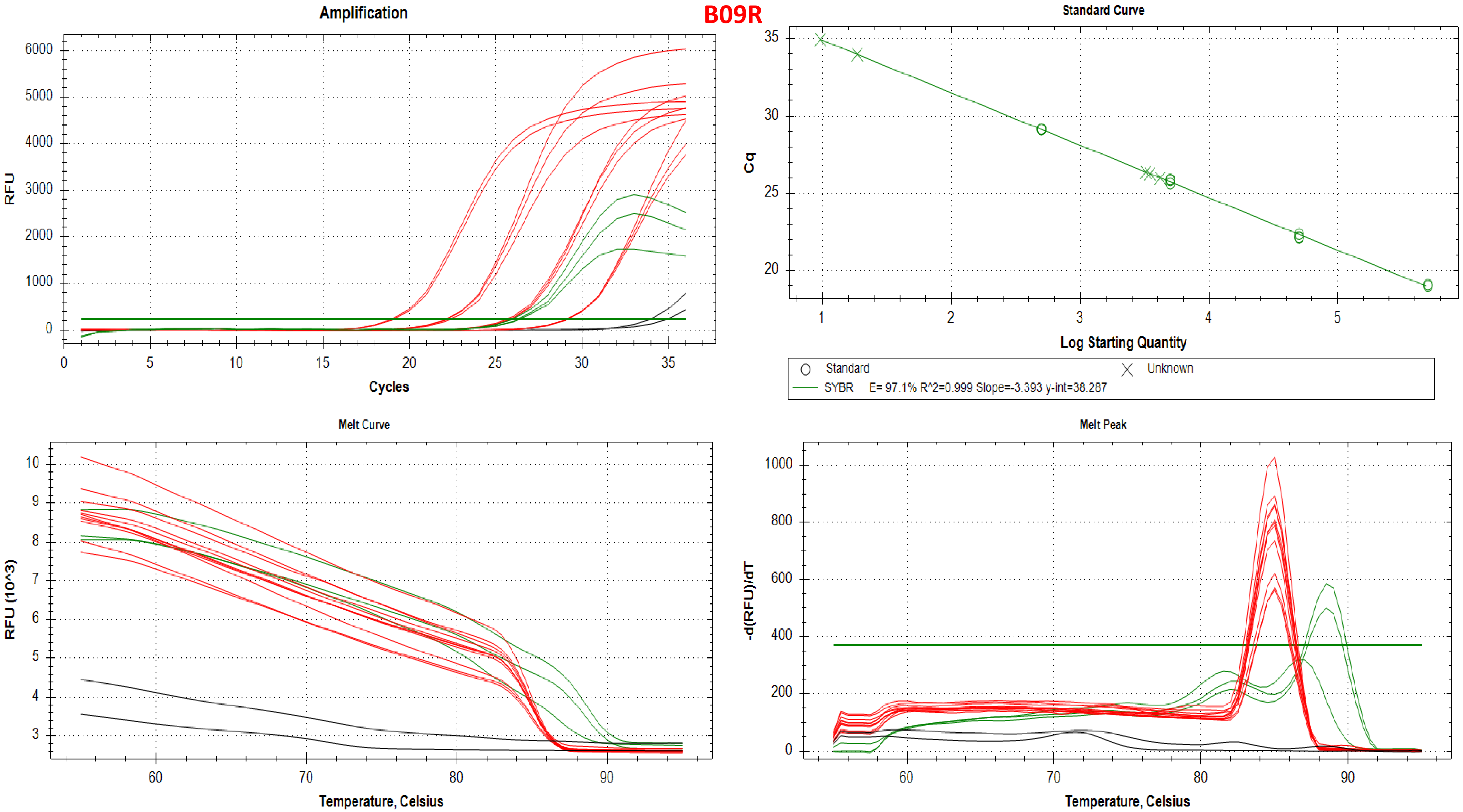
Amplification
RFU
Cycles
Standard Curve
Cq
Log Starting Quantity
Standard
Unknown
SYBR E= 97.1% R^2=0.999 Slope=-3.393 y-int=38.287
Melt Curve
RFU (10^3)
Temperature, Celsius
Melt Peak
-d(RFU)/dT
Temperature, Celsius

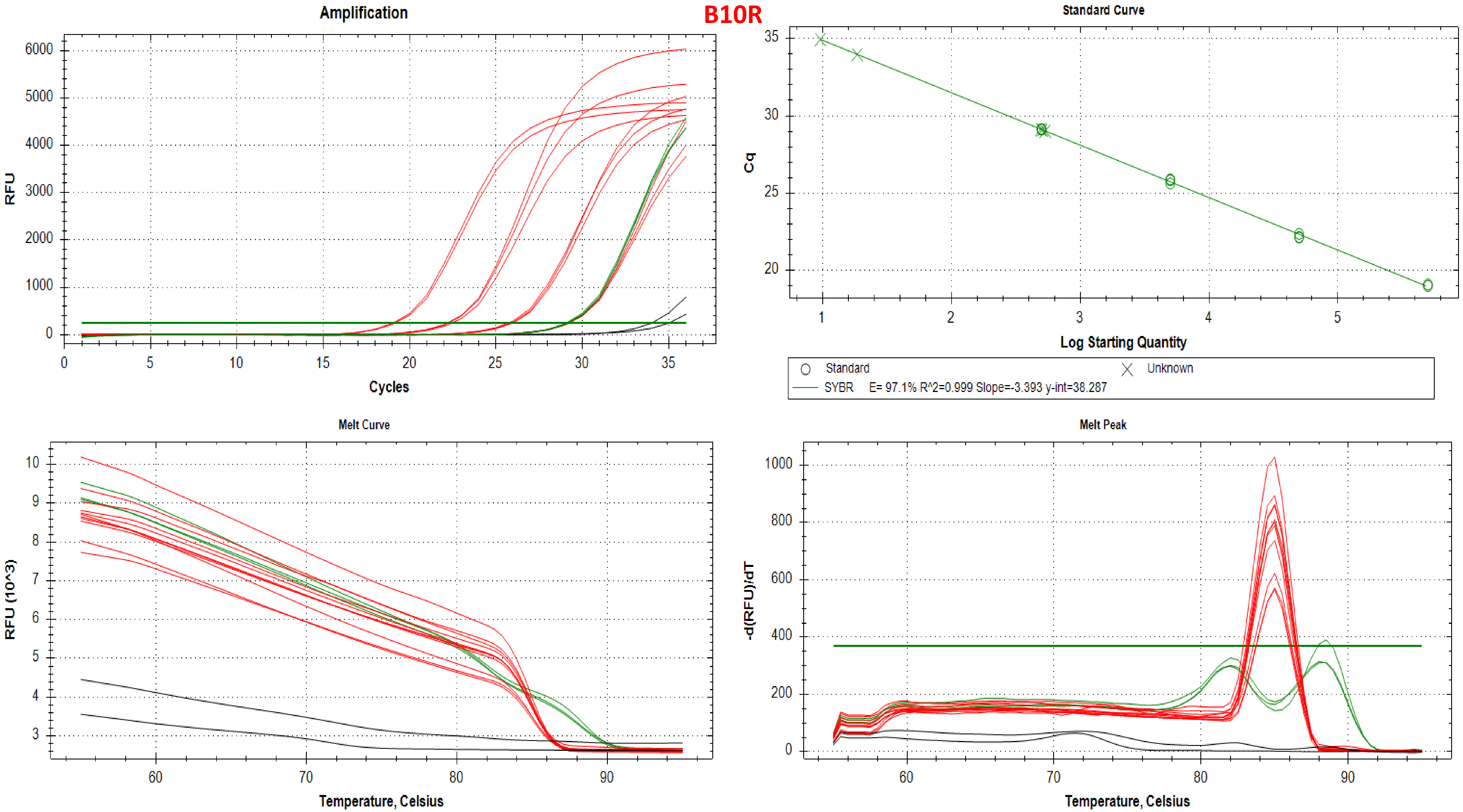
B10R
Amplification
RFU
6000
5000
4000
3000
2000
1000
0
0
5
10
15
20
25
30
35
Cycles
Standard Curve
Cq
35
30
25
20
1
2
3
4
5
Log Starting Quantity
Standard
Unknown
SYBR E= 97.1% R^2=0.999 Slope=-3.393 y-int=38.287
Melt Curve
RFU (10^3)
10
9
8
7
6
5
4
3
60
70
80
90
Temperature, Celsius
Melt Peak
-d(RFU)/dT
1000
800
600
400
200
0
60
70
80
90
Temperature, Celsius

B11R

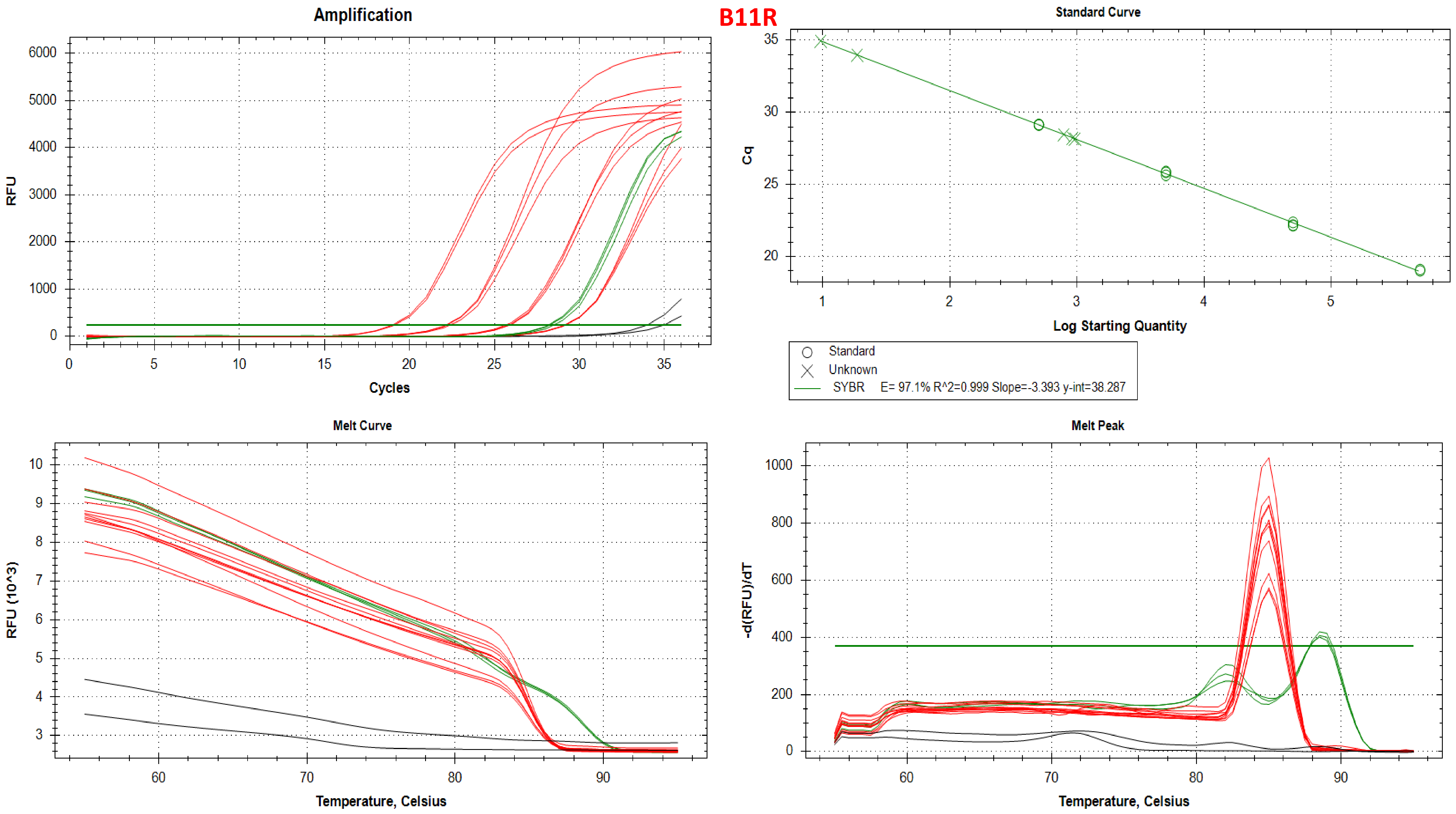

Amplification
RFU
Cycles
0
1000
2000
3000
4000
5000
6000
0
5
10
15
20
25
30
35
Standard Curve
Cq
Log Starting Quantity
20
25
30
35
1
2
3
4
5
Standard
Unknown
SYBR E= 97.1% R^2=0.999 Slope=-3.393 y-int=38.287
Melt Curve
RFU (10^3)
Temperature, Celsius
3
4
5
6
7
8
9
10
60
70
80
90
Melt Peak
-d(RFU)/dT
Temperature, Celsius
0
200
400
600
800
1000
60
70
80
90

B13R

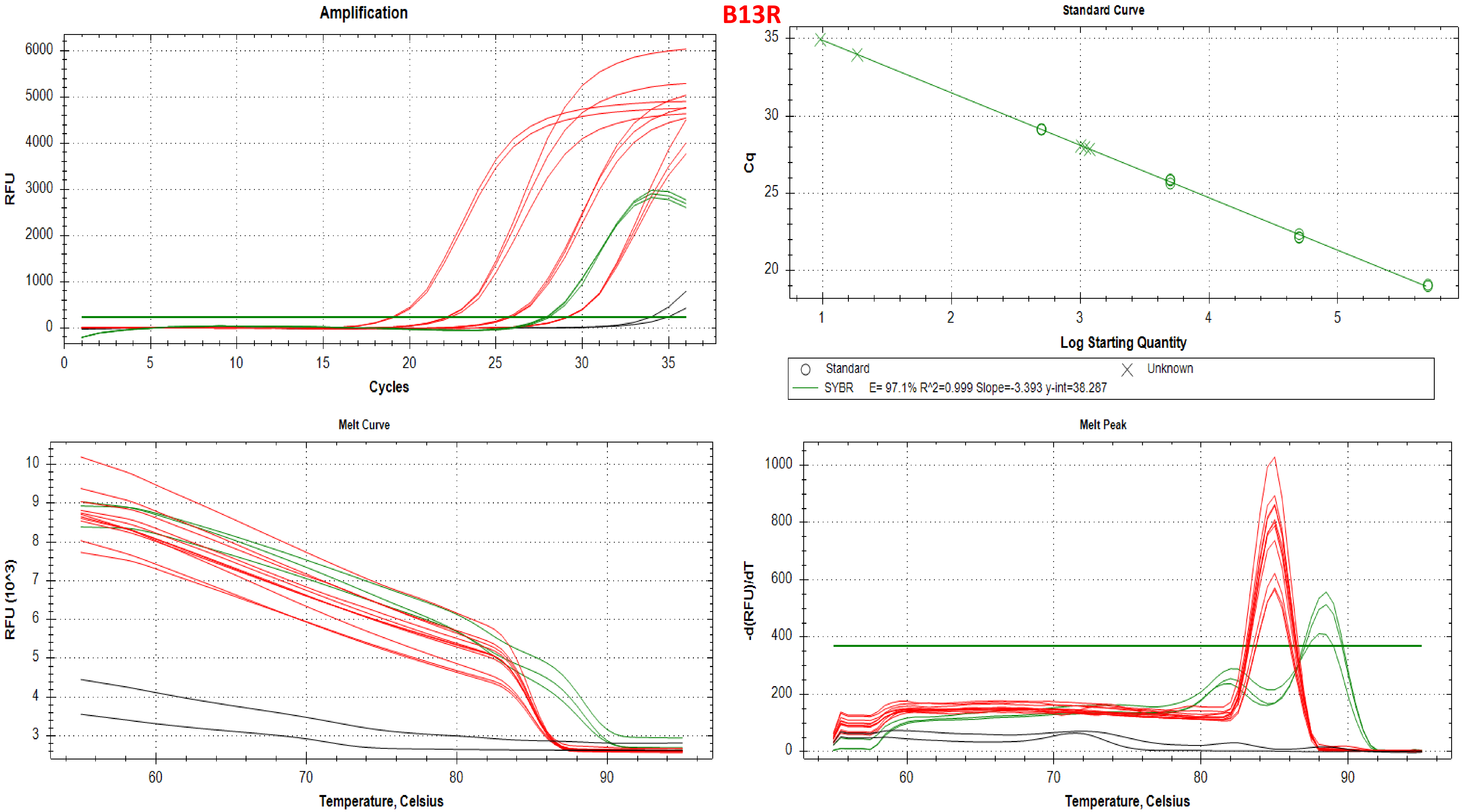

Amplification
RFU
Cycles
Standard Curve
Cq
Log Starting Quantity
Standard
Unknown
SYBR E= 97.1% R^2=0.999 Slope=-3.393 y-int=38.287
Melt Curve
RFU (10^3)
Temperature, Celsius
Melt Peak
-d(RFU)/dT
Temperature, Celsius

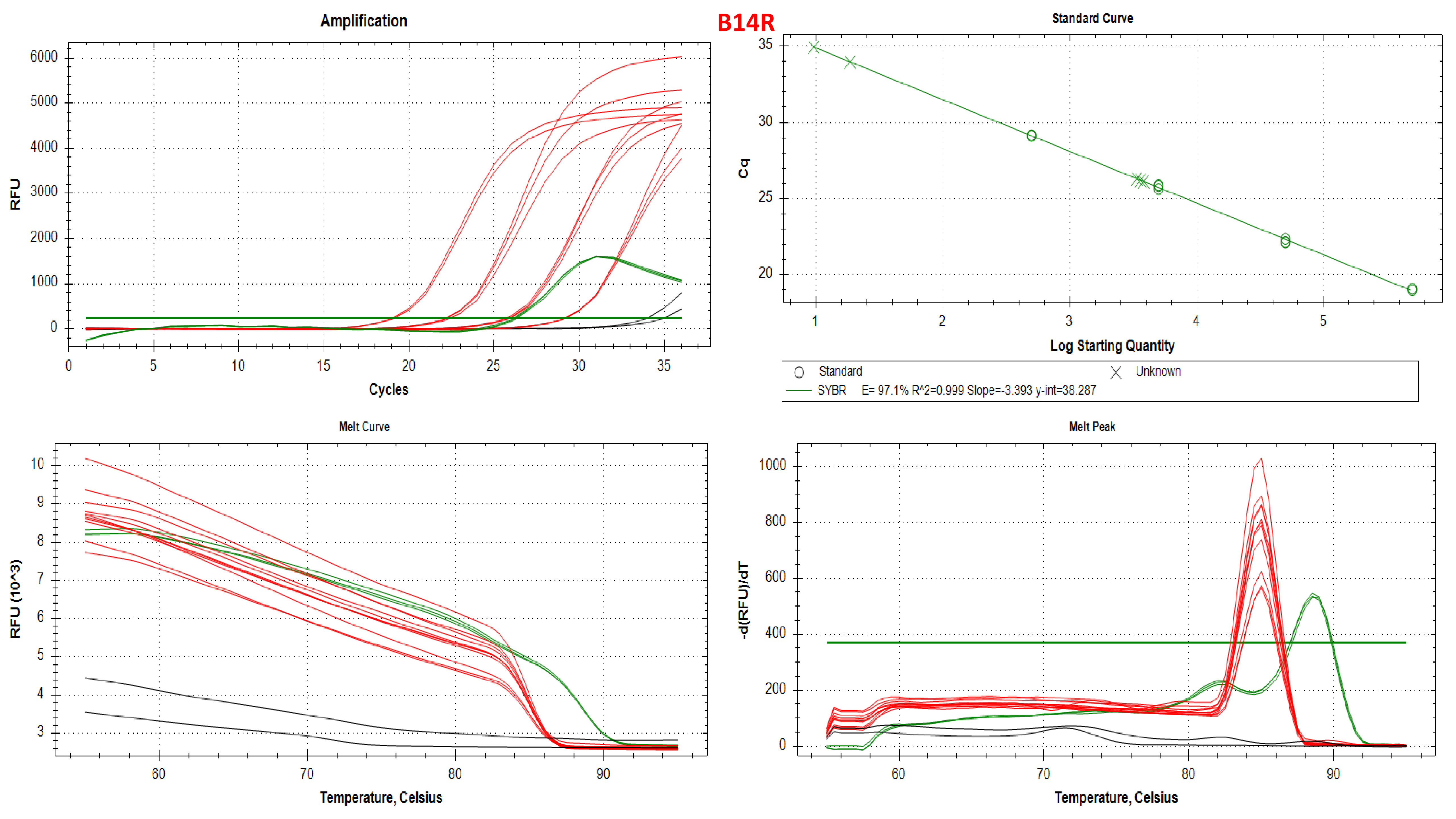
B14R
Amplification
RFU
Cycles
Standard Curve
Cq
Log Starting Quantity
Standard
Unknown
SYBR E= 97.1% R^2=0.999 Slope=-3.393 y-int=38.287
Melt Curve
RFU (10^3)
Temperature, Celsius
Melt Peak
-d(RFU)/dT
Temperature, Celsius

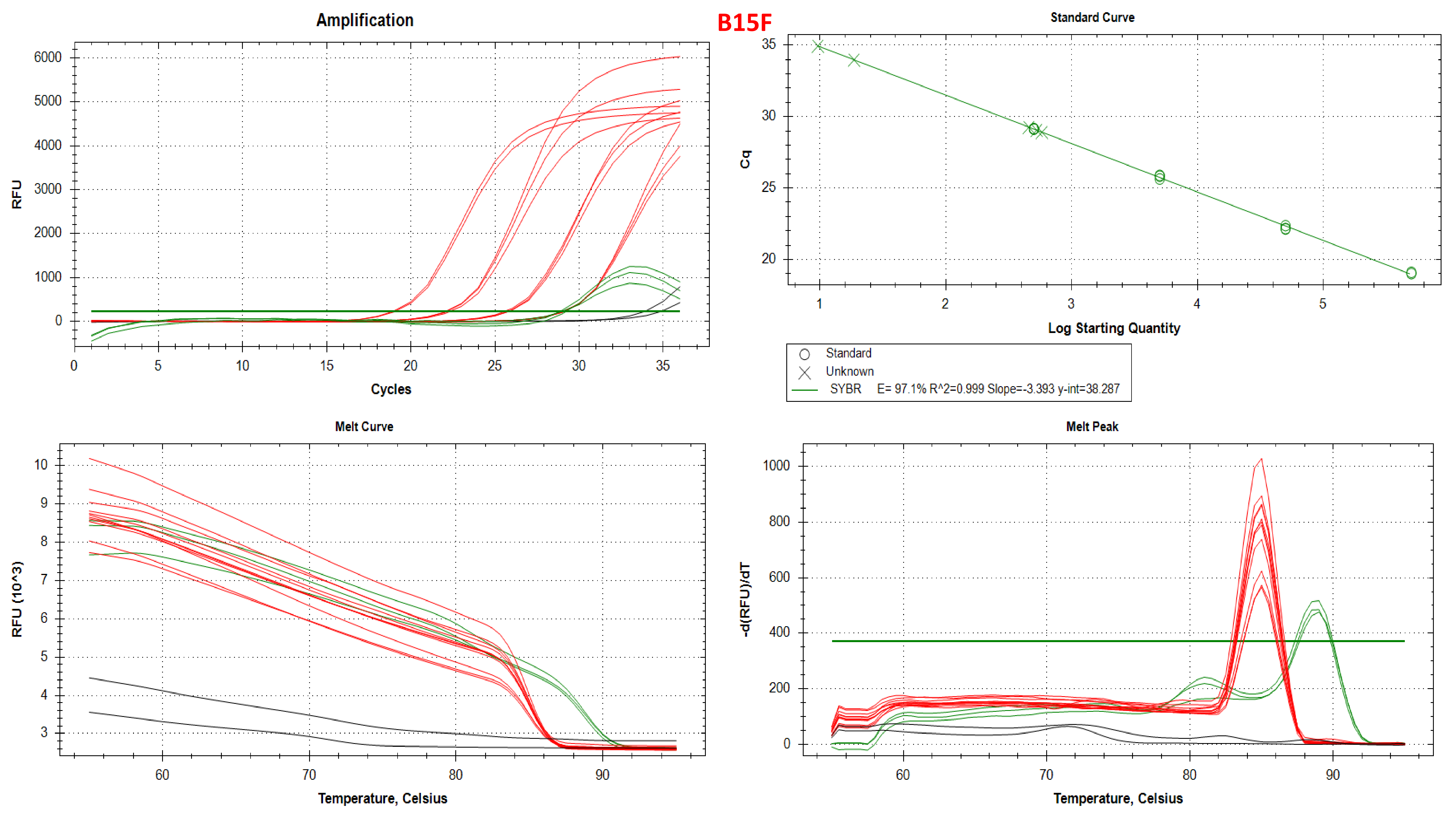
B15F
Amplification
RFU
Cycles
Standard Curve
Cq
Log Starting Quantity
Standard
Unknown
SYBR E= 97.1% R^2=0.999 Slope=-3.393 y-int=38.287
Melt Curve
RFU (10^3)
Temperature, Celsius
Melt Peak
-d(RFU)/dT
Temperature, Celsius

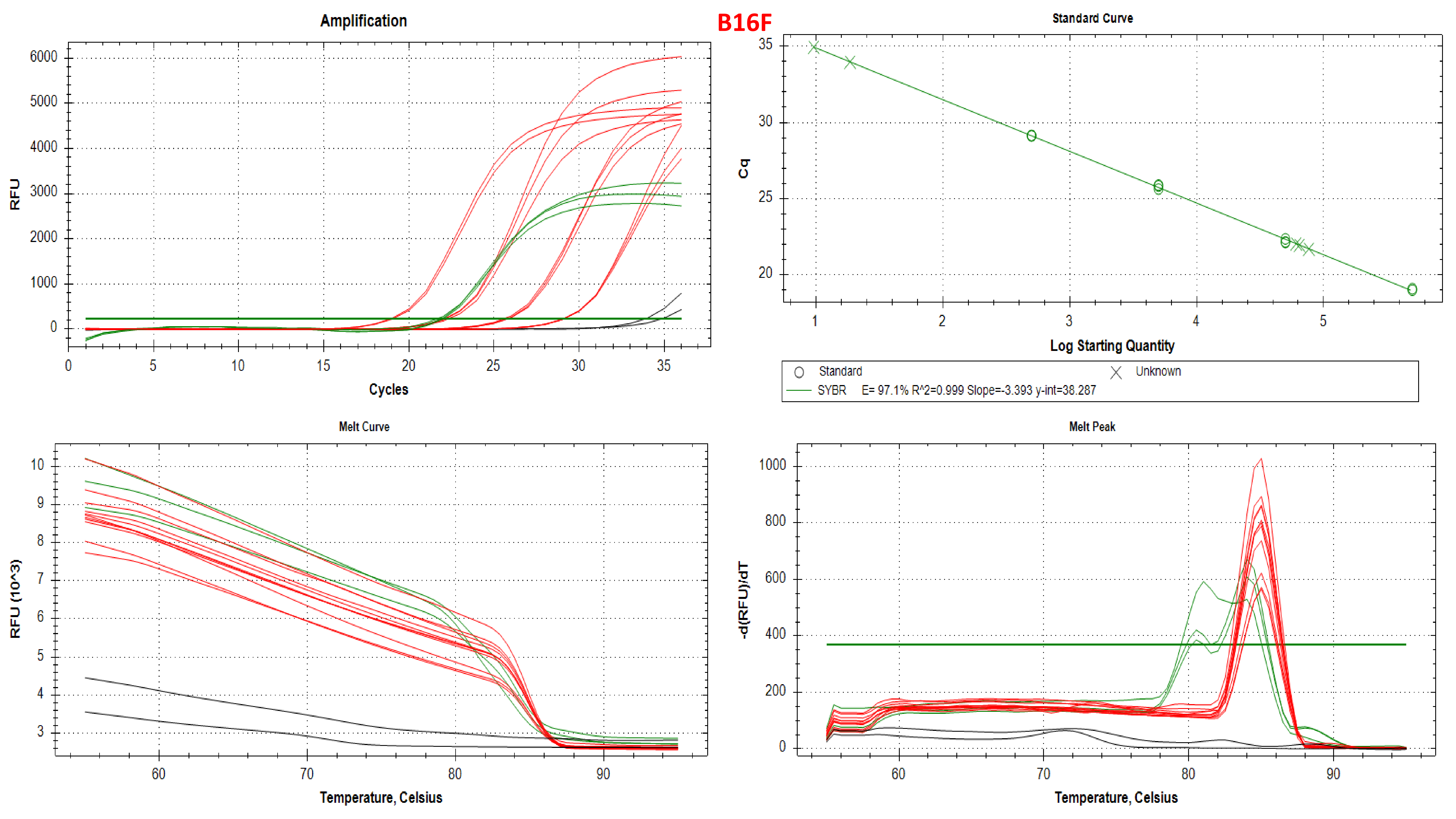
B16F
Amplification
RFU
Cycles
0
1000
2000
3000
4000
5000
6000
0
5
10
15
20
25
30
35
Standard Curve
Cq
Log Starting Quantity
20
25
30
35
1
2
3
4
5
Standard
Unknown
SYBR E= 97.1% R^2=0.999 Slope=-3.393 y-int=38.287
Melt Curve
RFU (10^3)
Temperature, Celsius
3
4
5
6
7
8
9
10
60
70
80
90
Melt Peak
-d(RFU)/dT
Temperature, Celsius
0
200
400
600
800
1000
60
70
80
90

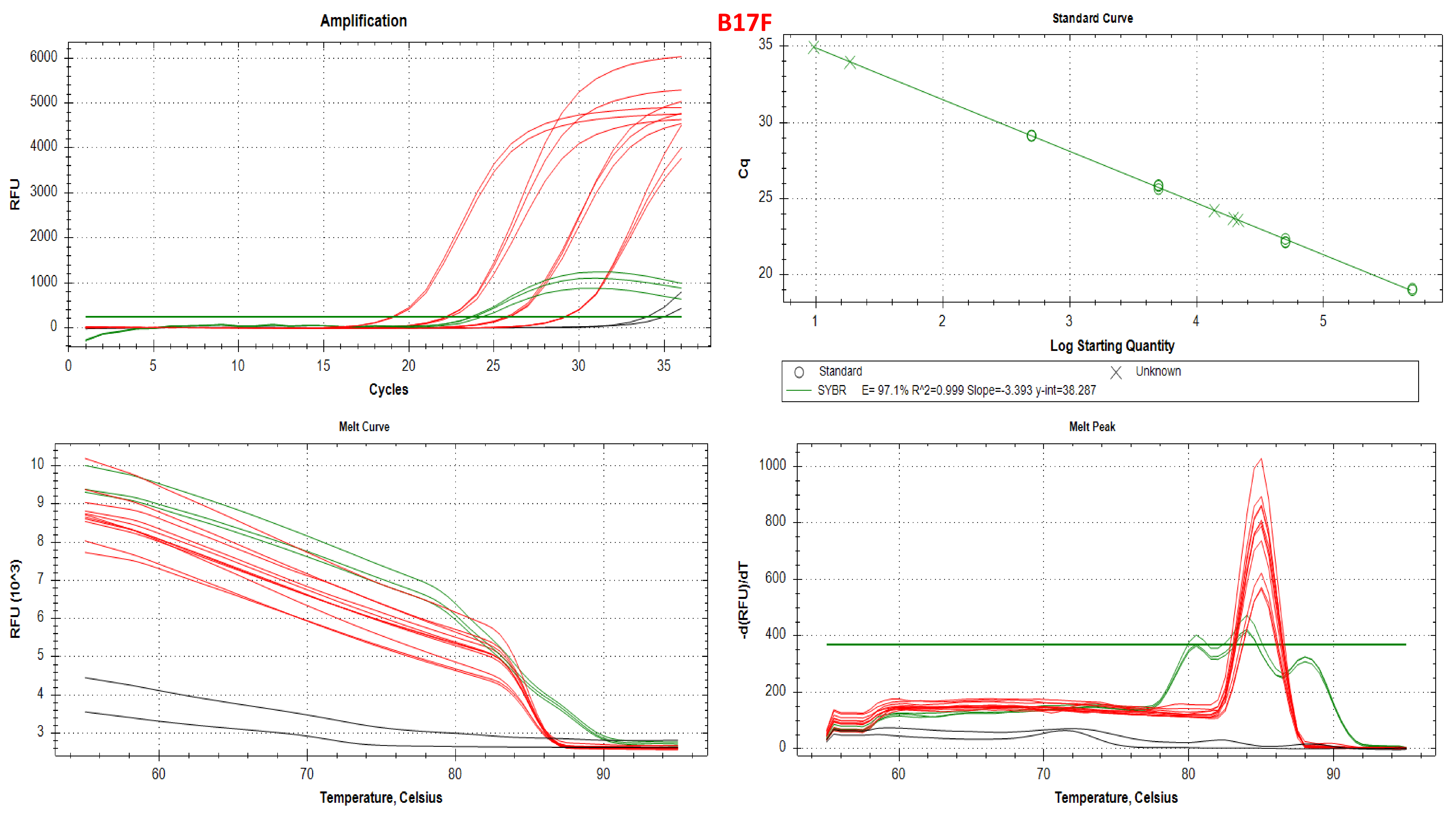
B17F
Amplification
RFU
Cycles
Standard Curve
Cq
Log Starting Quantity
Standard
Unknown
SYBR E= 97.1% R^2=0.999 Slope=-3.393 y-int=38.287
Melt Curve
RFU (10^3)
Temperature, Celsius
Melt Peak
-d(RFU)/dT
Temperature, Celsius

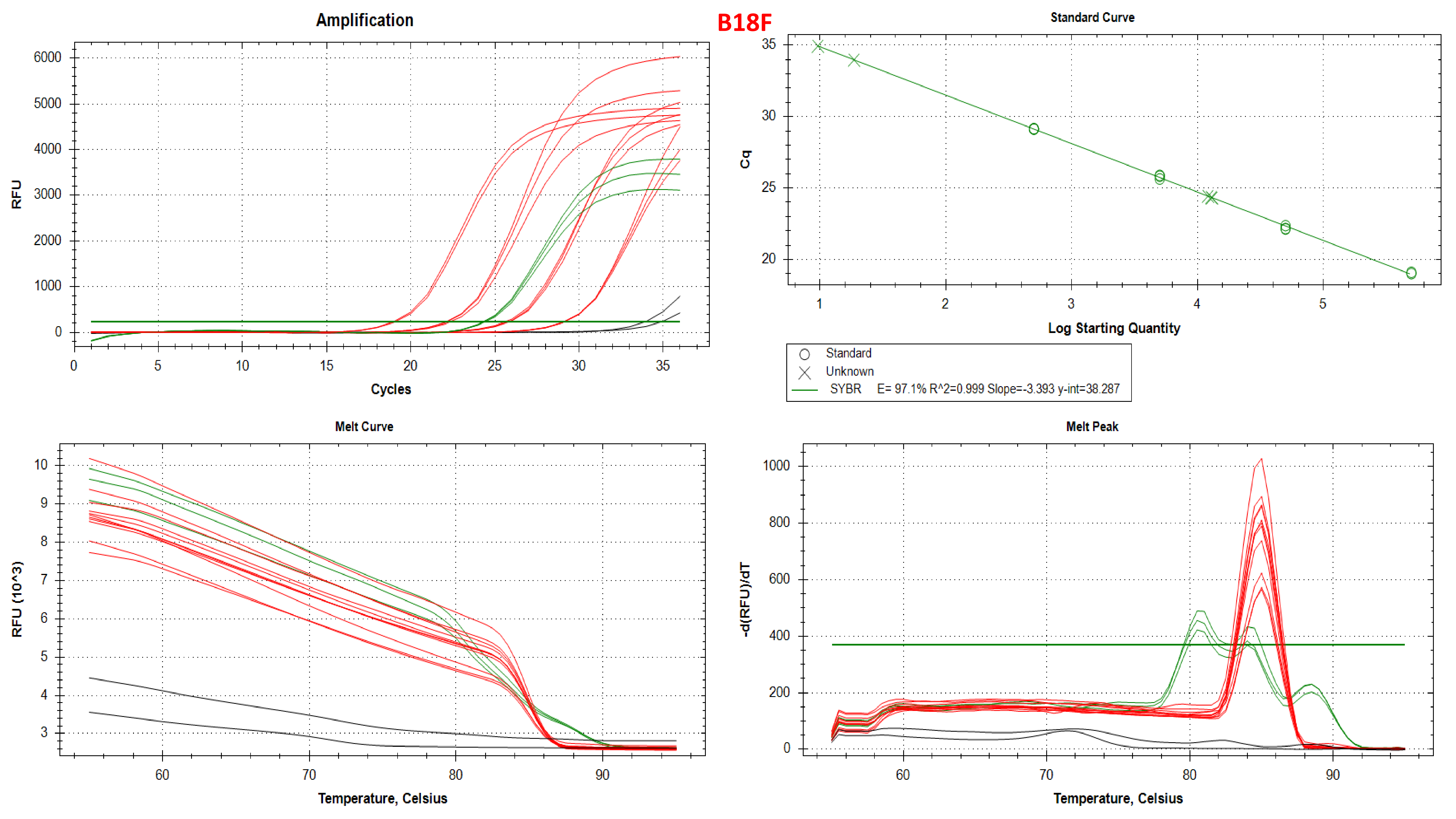

B18F
Amplification
RFU
Cycles
Standard Curve
Cq
Log Starting Quantity
Standard
Unknown
SYBR E= 97.1% R^2=0.999 Slope=-3.393 y-int=38.287
Melt Curve
RFU (10^3)
Temperature, Celsius
Melt Peak
-d(RFU)/dT
Temperature, Celsius

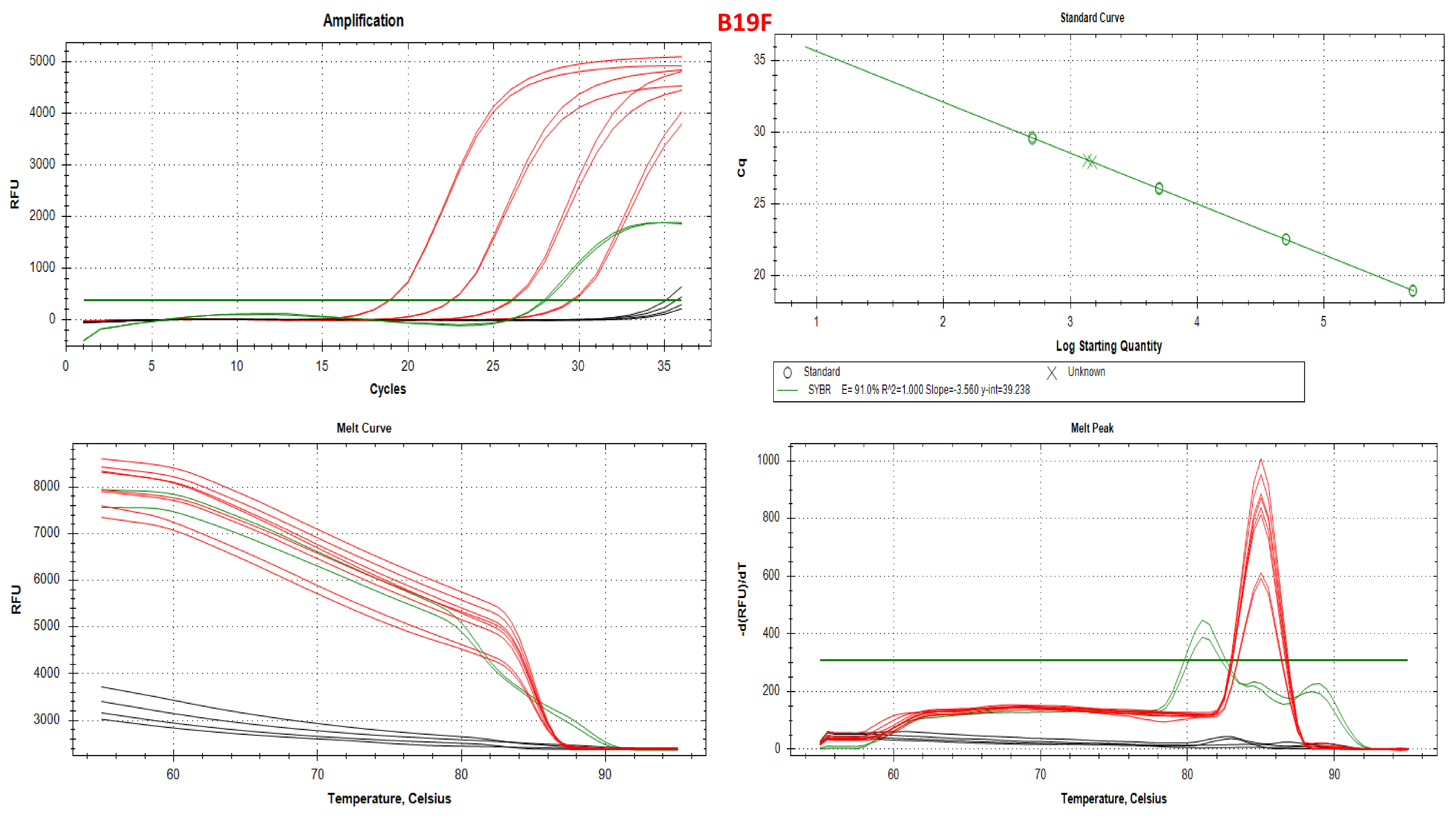
B19F
Amplification
RFU
Cycles
Standard Curve
Cq
Log Starting Quantity
Standard
Unknown
SYBR E= 91.0% R^2=1.000 Slope=-3.560 y-int=39.238
Melt Curve
RFU
Temperature, Celsius
Melt Peak
-d(RFU)/dT
Temperature, Celsius

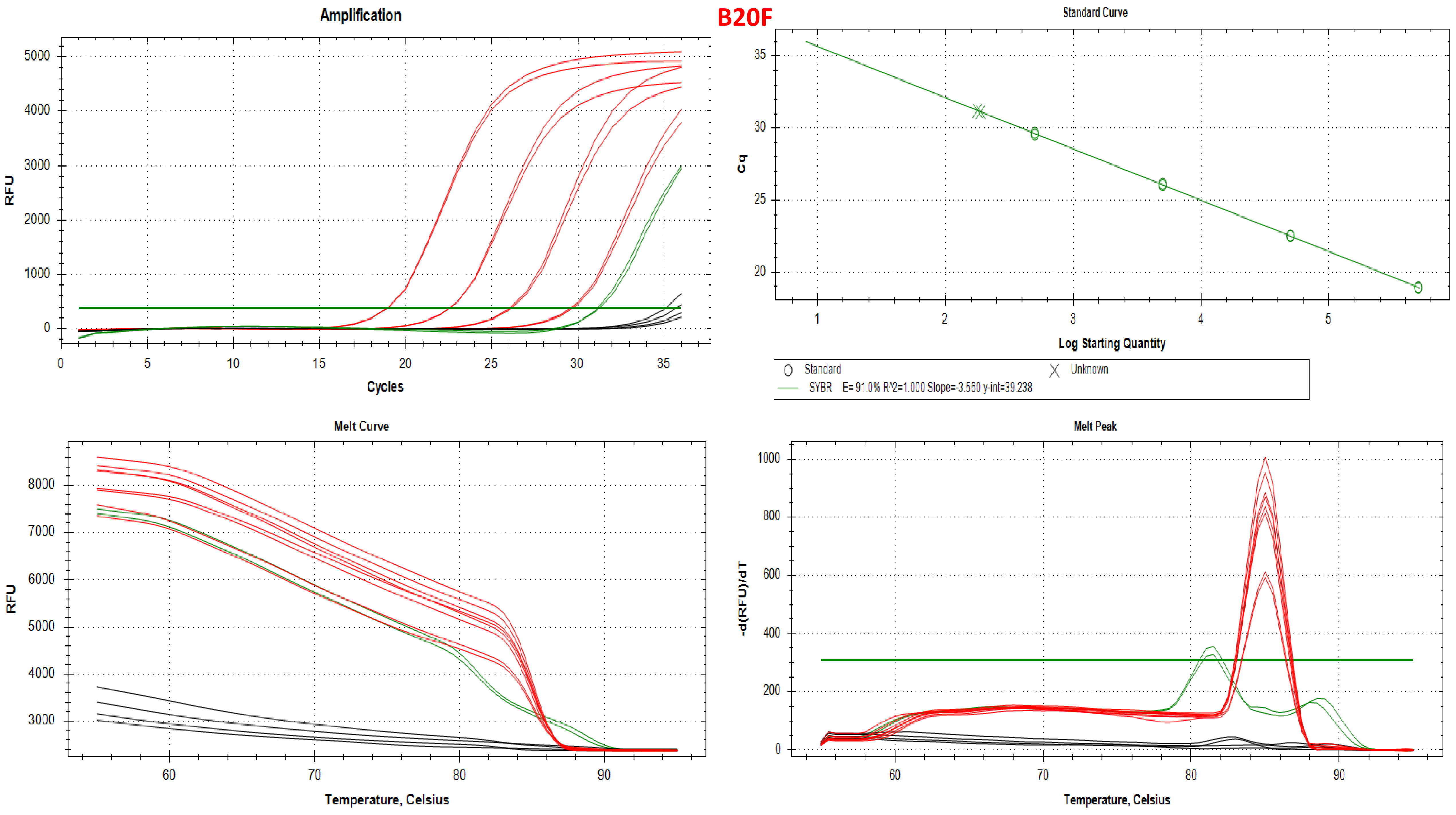
B20F
Amplification
RFU
Cycles
Standard Curve
Cq
Log Starting Quantity
Standard
Unknown
SYBR E= 91.0% R^2=1.000 Slope=-3.560 y-int=39.238
Melt Curve
RFU
Temperature, Celsius
Melt Peak
-d(RFU)/dT
Temperature, Celsius

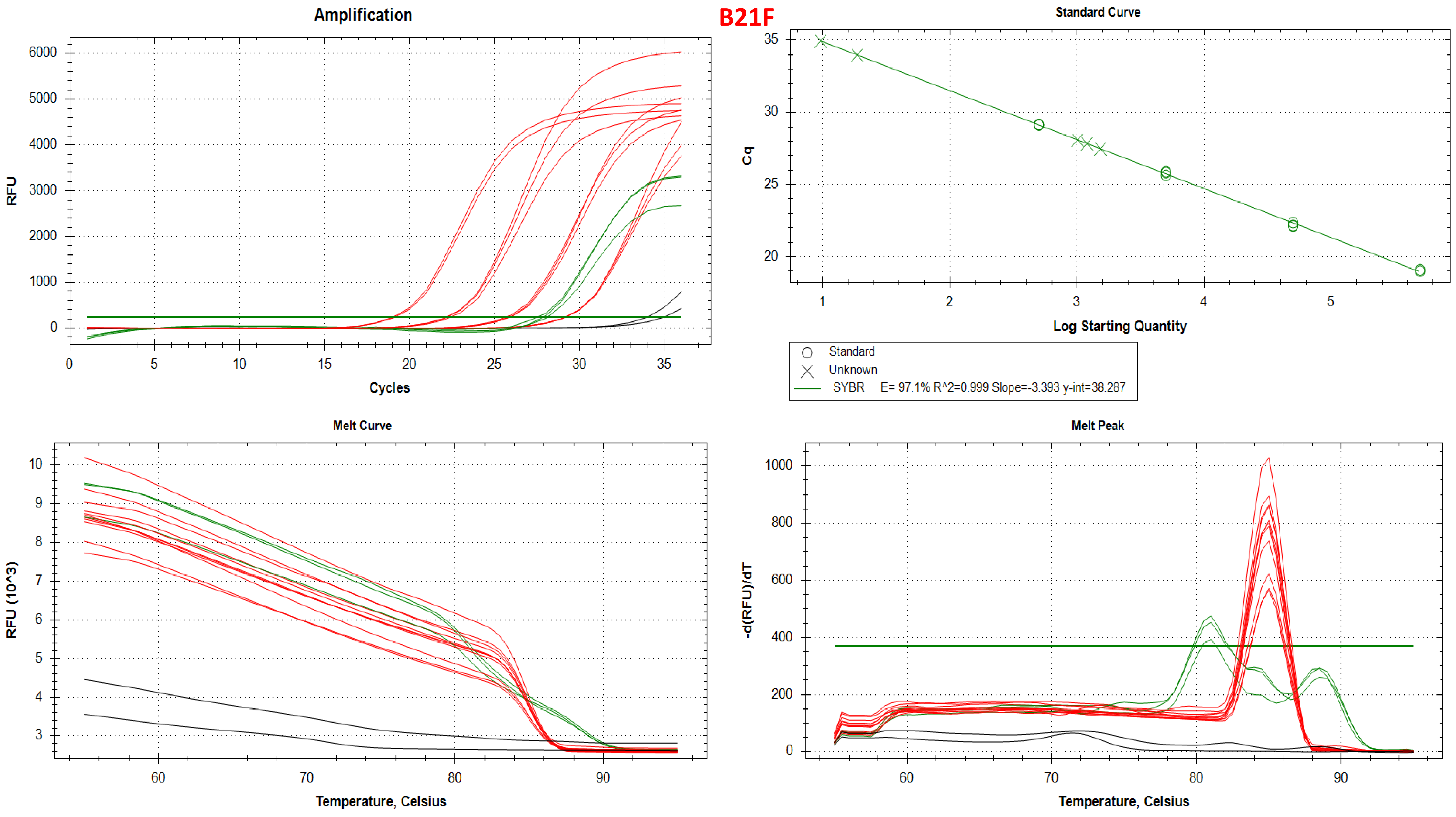
B21F
Amplification
RFU
6000
5000
4000
3000
2000
1000
0
0
5
10
15
20
25
30
35
Cycles
Standard Curve
Cq
35
30
25
20
1
2
3
4
5
Log Starting Quantity
Standard
Unknown
SYBR E= 97.1% R^2=0.999 Slope=-3.393 y-int=38.287
Melt Curve
RFU (10^3)
10
9
8
7
6
5
4
3
60
70
80
90
Temperature, Celsius
Melt Peak
-d(RFU)/dT
1000
800
600
400
200
0
60
70
80
90
Temperature, Celsius

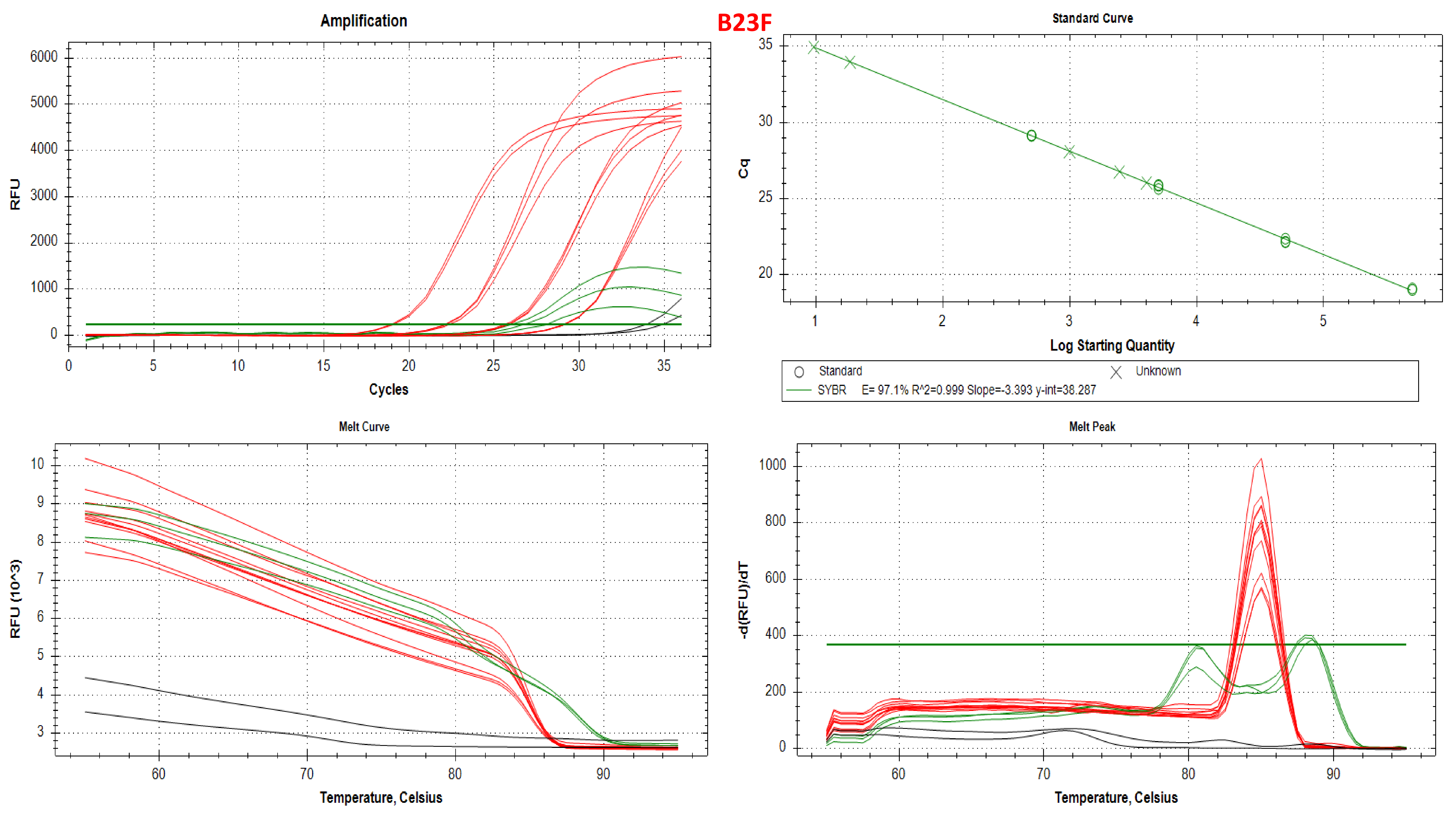

B23F
Amplification
RFU
6000
5000
4000
3000
2000
1000
0
0
5
10
15
20
25
30
35
Cycles
Standard Curve
Cq
35
30
25
20
1
2
3
4
5
Log Starting Quantity
Standard
Unknown
SYBR E= 97.1% R^2=0.999 Slope=-3.393 y-int=38.287
Melt Curve
RFU (10^3)
10
9
8
7
6
5
4
3
60
70
80
90
Temperature, Celsius
Melt Peak
-d(RFU)/dT
1000
800
600
400
200
0
60
70
80
90
Temperature, Celsius

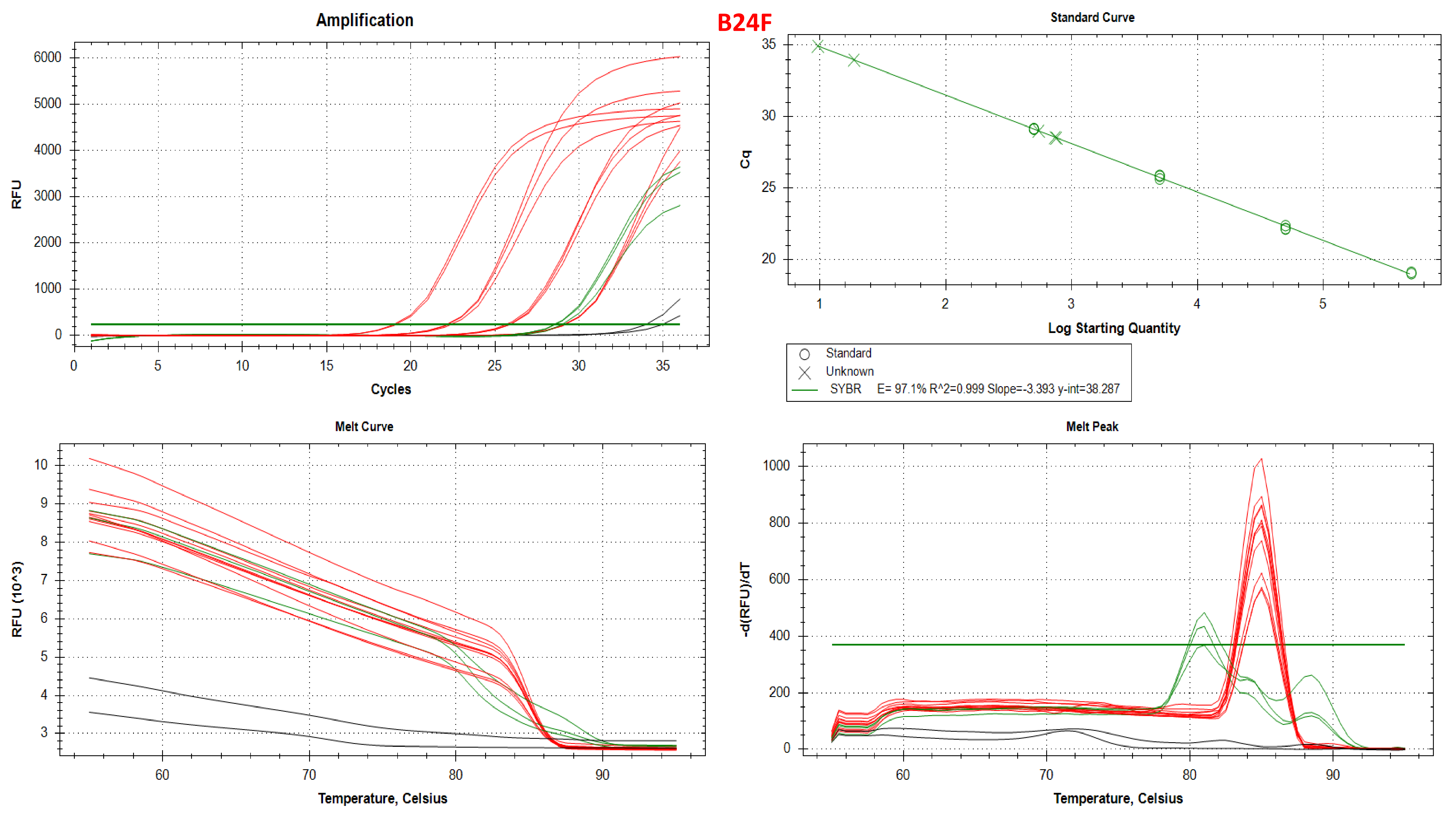

B24F
Amplification
RFU
Cycles
Standard Curve
Cq
Log Starting Quantity
Standard
Unknown
SYBR E= 97.1% R^2=0.999 Slope=-3.393 y-int=38.287
Melt Curve
RFU (10^3)
Temperature, Celsius
Melt Peak
-d(RFU)/dT
Temperature, Celsius

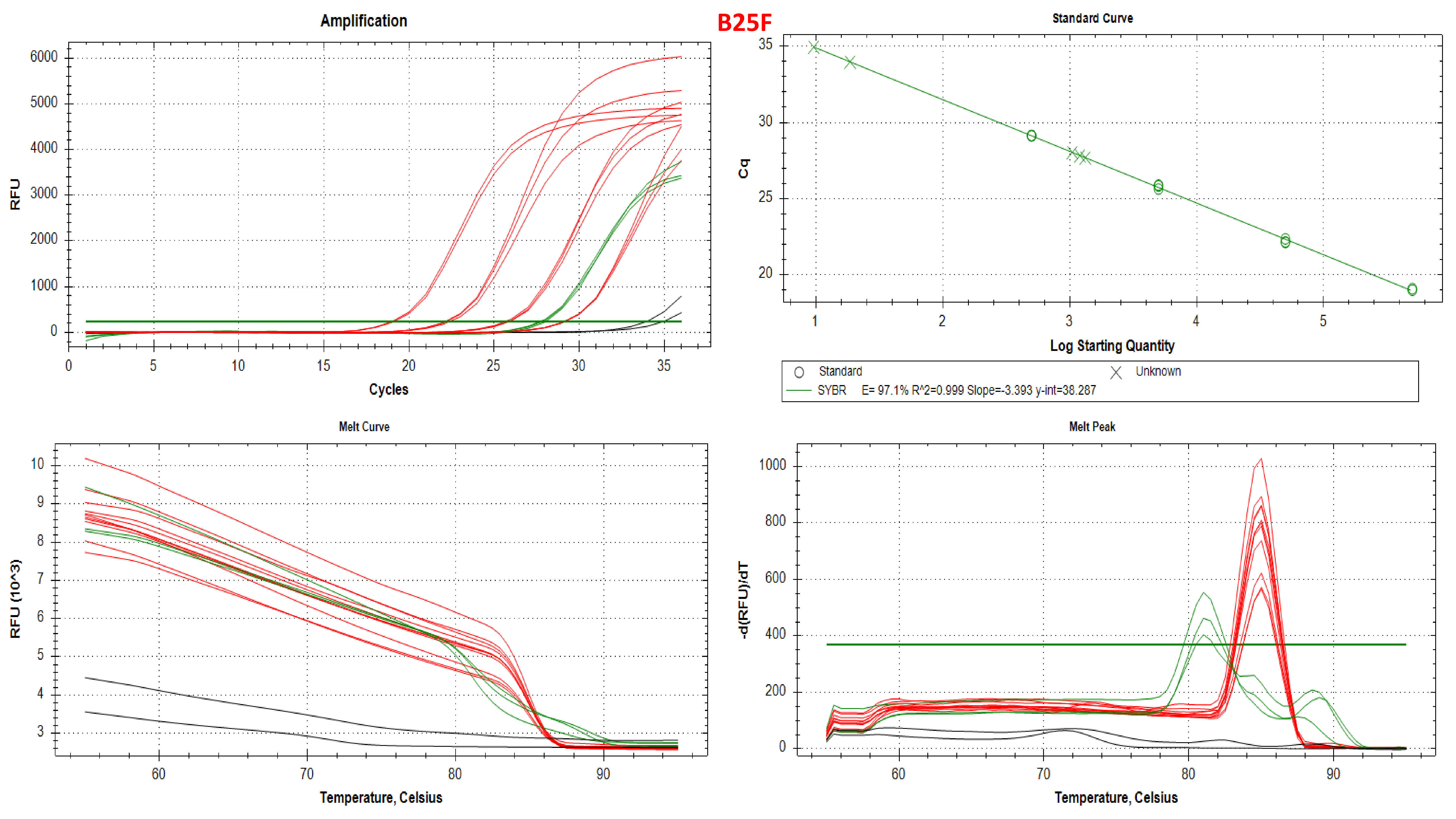
B25F
Amplification
RFU
Cycles
Standard Curve
Cq
Log Starting Quantity
Standard
Unknown
SYBR E= 97.1% R^2=0.999 Slope=-3.393 y-int=38.287
Melt Curve
RFU (10^3)
Temperature, Celsius
Melt Peak
-d(RFU)/dT
Temperature, Celsius

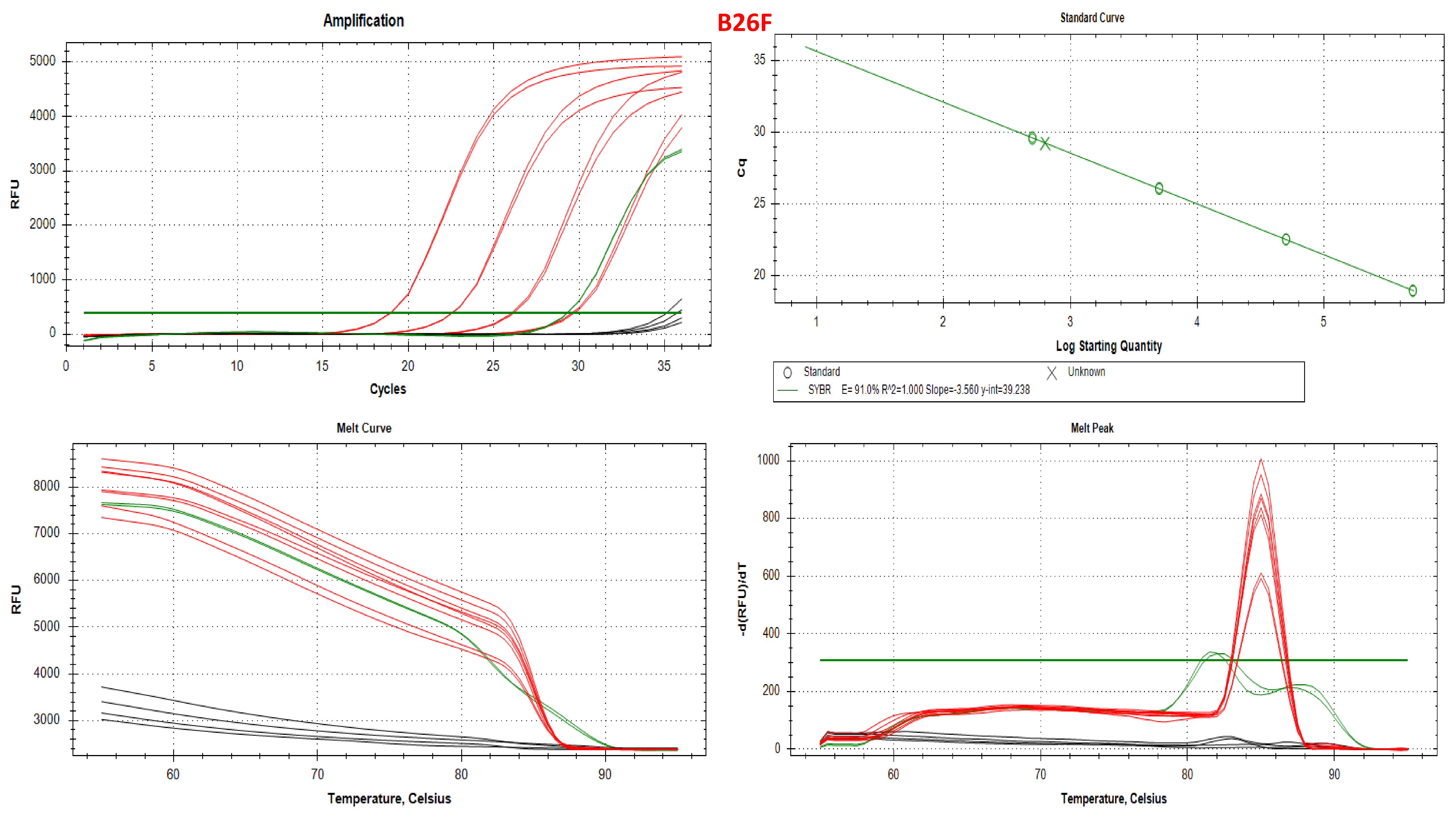
B26F
Amplification
RFU
Cycles
Standard Curve
Cq
Log Starting Quantity
Standard
Unknown
SYBR E= 91.0% R^2=1.000 Slope=-3.560 y-int=39.238
Melt Curve
RFU
Temperature, Celsius
Melt Peak
-d(RFU)/dT
Temperature, Celsius

B27F

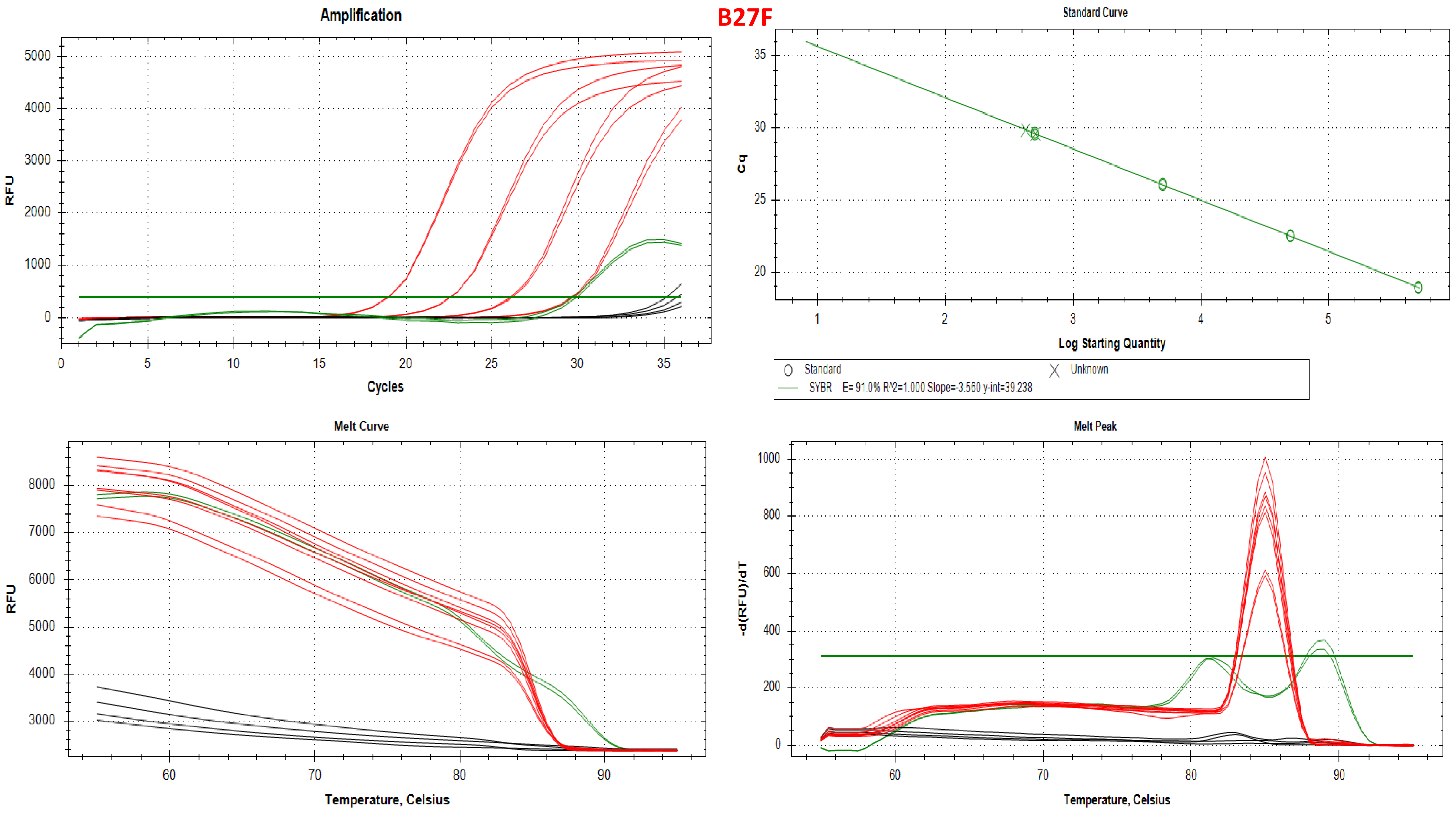
Amplification
RFU
5000
4000
3000
2000
1000
0
0
5
10
15
20
25
30
35
Cycles
Standard Curve
Cq
35
30
25
20
1
2
3
4
5
Log Starting Quantity
Standard
Unknown
SYBR E= 91.0% R^2=1.000 Slope=-3.560 y-int=39.238
Melt Curve
RFU
8000
7000
6000
5000
4000
3000
60
70
80
90
Temperature, Celsius
Melt Peak
-d(RFU)/dT
1000
800
600
400
200
0
60
70
80
90
Temperature, Celsius

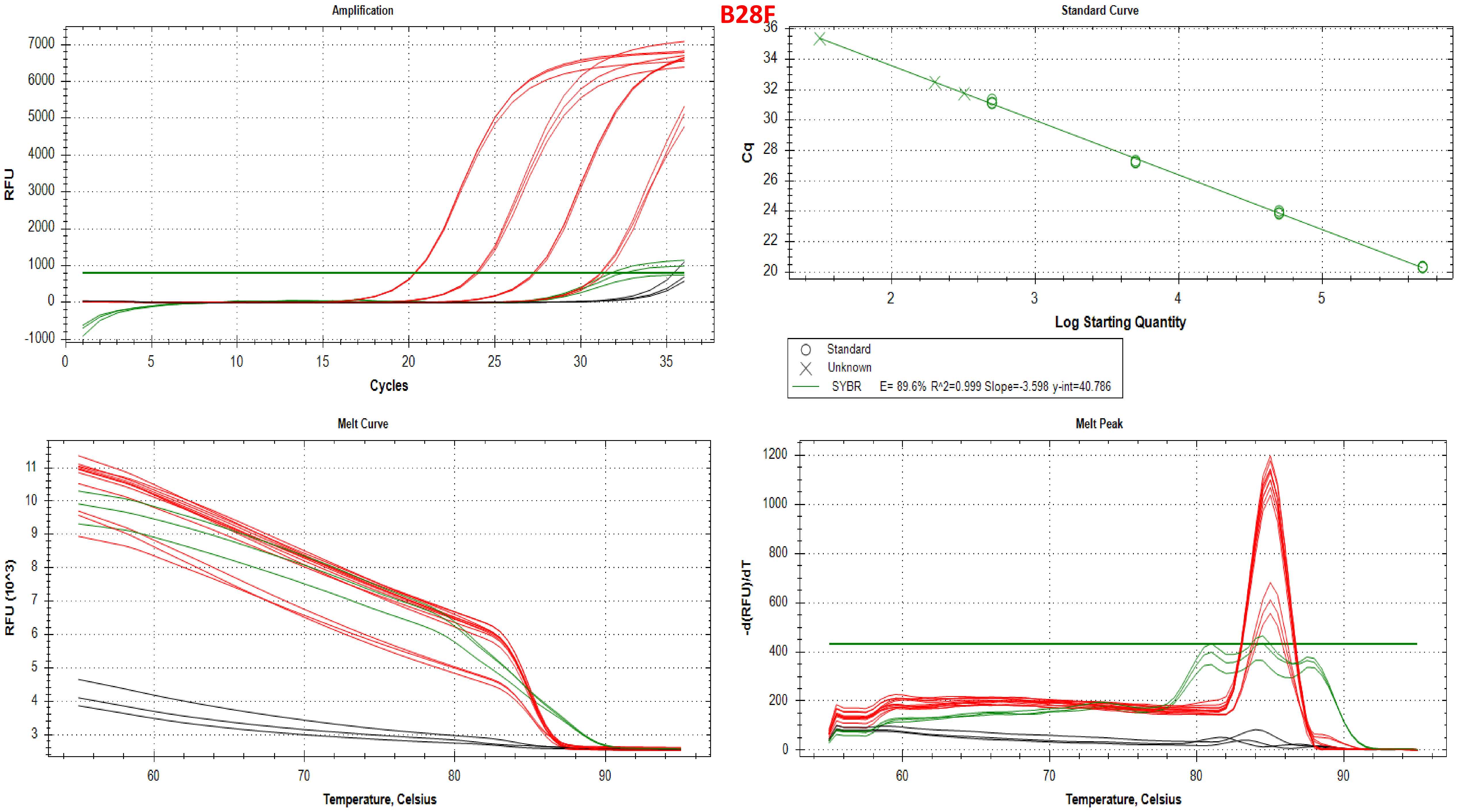

B28F
Amplification
RFU
Cycles
Standard Curve
Cq
Log Starting Quantity
Standard
Unknown
SYBR E= 89.6% R^2=0.999 Slope=-3.598 y-int=40.786
Melt Curve
RFU (10^3)
Temperature, Celsius
Melt Peak
-d(RFU)/dT
Temperature, Celsius

Figure S2: 16S DNA sequencing : Rarefaction curves for each sample

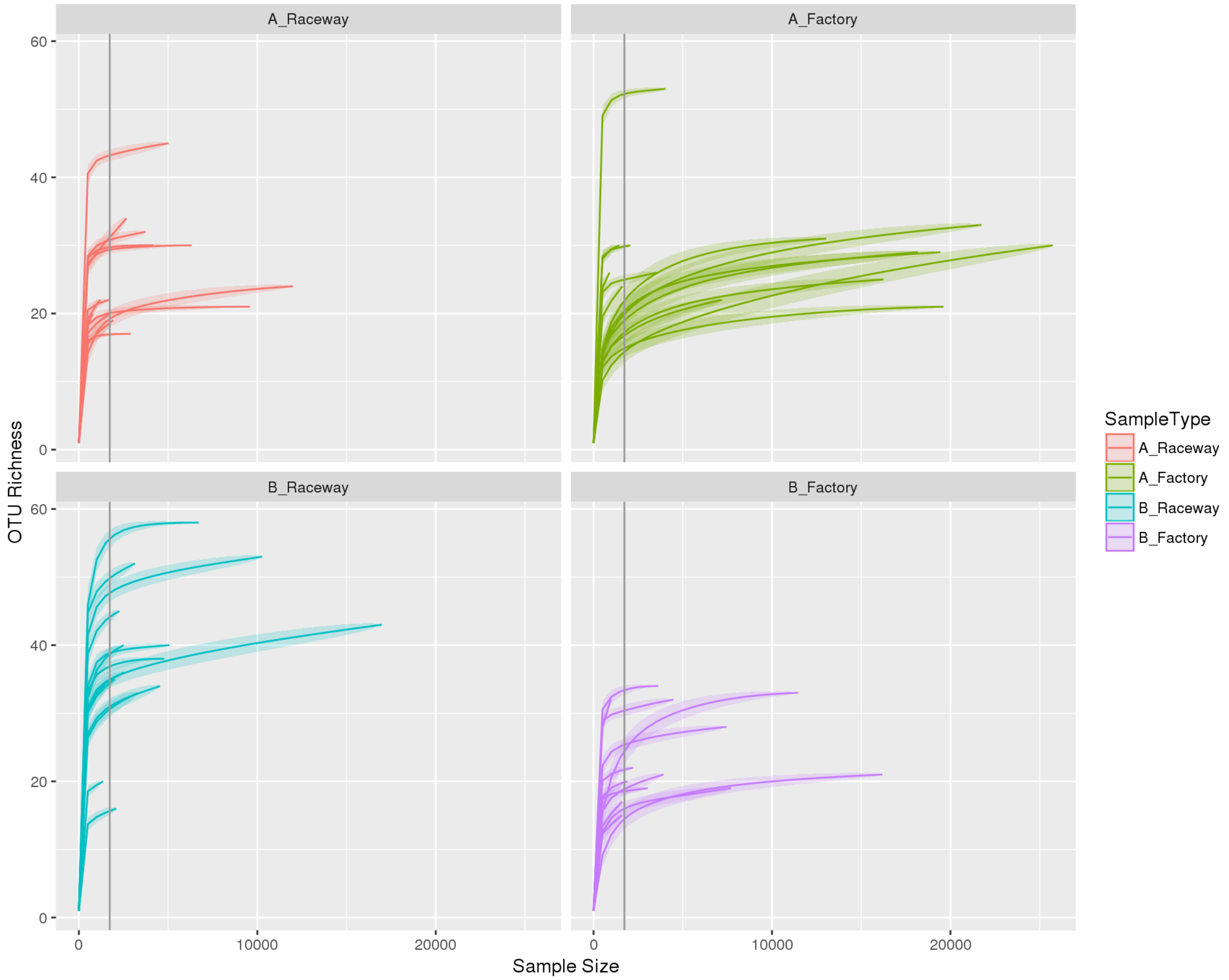

Supplement: Supplementary Figure 1 — PCR targeting gene tuf data. Standard curves chart, amplification chart, melting curve chart and melting peak chart for each samples. Bacterial loads were calculated from standard curves as described in 3.4. [file Image_1.PDF]
