# Supplementary material for: Antibiotic Resistance Genes and Bacterial Communities of Farmed Rainbow Trout Fillets (Oncorhynchus mykiss)
Source: Front Microbiol. 2020 Dec 3;11:590902. doi: 10.3389/fmicb.2020.590902 (PMC7744637; doi:10.3389/fmicb.2020.590902)
Supplement: Supplementary Table 1 — List of primers used in the qPCR array. Primer names indicate the targeted genes. [file Table_1.DOCX]

**Table S1**. List of primers used in the qPCR array. Primers names indicate targeted genes.

| **Primer pair #** | **Primer Name** | **Sequence (5’ 🡪 3’)** |
| --- | --- | --- |
| 1 | 16S-01 R | GGGTTGCGCTCGTTGC |
|  | 16S-01 F | ATGGYTGTCGTCAGCTCGTG |
| 2 | 16S-02 R | CCTACGGGAGGCAGCAG |
|  | 16S-02 F | ATTACCGCGGCTGCTGGC |
| 3 | aac(6')-I F | GACCGGATTAAGGCCGATG |
|  | aac(6')-I R | CTTGCCTTGATATTCAGTTTTTATAACCA |
| 4 | aac(6')-Ib-01 F | GTTTGAGAGGCAAGGTACCGTAA |
|  | aac(6')-Ib-01 R | GAATGCCTGGCGTGTTTGA |
| 5 | aac(6')-Ib-02 F | CGTCGCCGAGCAACTTG |
|  | aac(6')-Ib-02 R | CGGTACCTTGCCTCTCAAACC |
| 6 | aac(6')-Ib-03 F | AGAAGCACGCCCGACACTT |
|  | aac(6')-Ib-03 R | GCTCTCCATTCAGCATTGCA |
| 7 | aac(6')-II F | CGACCCGACTCCGAACAA |
|  | aac(6')-II R | GCACGAATCCTGCCTTCTCA |
| 8 | aacA F | AGAGCCTTGGGAAGATGAAGTTT |
|  | aacA R | TTGATCCATACCATAGACTATCTCATCA |
| 9 | aacC F | CGTCACTTATTCGATGCCCTTAC |
|  | aacC R | GTCGGGCGCGGCATA |
| 10 | aacC2 F | ACGGCATTCTCGATTGCTTT |
|  | aacC2 R | CCGAGCTTCACGTAAGCATTT |
| 11 | aadA-01 F | GTTGTGCACGACGACATCATT |
|  | aadA-01 R | GGCTCGAAGATACCTGCAAGAA |
| 12 | aadA-02 F | CGAGATTCTCCGCGCTGTA |
|  | aadA-02 R | GCTGCCATTCTCCAAATTGC |
| 13 | aadA1 F | AGCTAAGCGCGAACTGCAAT |
|  | aadA1 R | TGGCTCGAAGATACCTGCAA |
| 14 | aadA2-01 F | ACGGCTCCGCAGTGGAT |
|  | aadA2-01 R | GGCCACAGTAACCAACAAATCA |
| 15 | aadA2-02 F | CTTGTCGTGCATGACGACATC |
|  | aadA2-02 R | TCGAAGATACCCGCAAGAATG |
| 16 | aadA2-03 F | CAATGACATTCTTGCGGGTATC |
|  | aadA2-03 R | GACCTACCAAGGCAACGCTATG |
| 17 | aadA5-01 F | ATCACGATCTTGCGATTTTGCT |
|  | aadA5-01 R | CTGCGGATGGGCCTAGAAG |
| 18 | aadA5-02 F | GTTCTTGCTCTTGCTCGCATT |
|  | aadA5-02 R | GATGCTCGGCAGGCAAAC |
| 19 | aadA9-01 F | CGCGGCAAGCCTATCTTG |
|  | aadA9-01 R | CAAATCAGCGACCGCAGACT |
| 20 | aadA9-02 F | GGATGCACGCTTGGATGAA |
|  | aadA9-02 R | CCTCTAGCGGCCGGAGTATT |
| 21 | aadD F | CCGACAACATTTCTACCATCCTT |
|  | aadD R | ACCGAAGCGCTCGTCGTATA |
| 22 | aadE F | TACCTTATTGCCCTTGGAAGAGTTA |
|  | aadE R | GGAACTATGTCCCTTTTAATTCTACAATCT |
| 23 | acrA-01 F | CAACGATCGGACGGGTTTC |
|  | acrA-01 R | TGGCGATGCCACCGTACT |
| 24 | acrA-02 F | GGTCTATCACCCTACGCGCTATC |
|  | acrA-02 R | GCGCGCACGAACATACC |
| 25 | acrA-04 F | TACTTTGCGCGCCATCTTC |
|  | acrA-04 R | CGTGCGCGAACGAACAT |
| 26 | acrA-05 F | CGTGCGCGAACGAACA |
|  | acrA-05 R | ACTTTGCGCGCCATCTTC |
| 27 | acrB-01 F | AGTCGGTGTTCGCCGTTAAC |
|  | acrB-01 R | CAAGGAAACGAACGCAATACC |
| 28 | acrB-02 F | TGGTAGTGGGCGTCATTAACAC |
|  | acrB-02 R | GGCAACGTAATCCGAAATATCC |
| 29 | acrF F | GCGGCCAGGCACAAAA |
|  | acrF R | TACGCTCTTCCCACGGTTTC |
| 30 | acrR-01 F | GCGCTGGAGACACGACAAC |
|  | acrR-01 R | GCCTTGCTGCGAGAACAAA |
| 31 | acrR-02 F | GATGATACCCCCTGCTGTGAGA |
|  | acrR-02 R | ACCAAACAAGAAGCGCAAGAA |
| 32 | adeA F | CAGTTCGAGCGCCTATTTCTG |
|  | adeA R | CGCCCTGACCGACCAAT |
| 33 | ampC-02 F | GCAGCACGCCCCGTAA |
|  | ampC-02 R | TGTACCCATGATGCGCGTACT |
| 34 | ampC-03 F | AACAAAAGATCCCCGGTATGG |
|  | ampC-03 R | ACGCCCGTAAATGTTTTGCT |
| 35 | aph F | TTTCAGCAAGTGGATCATGTTAAAAT |
|  | aph R | CCAAGCTGTTTCCACTGTTTTTC |
| 36 | aphA1 F | TGAACAAGTCTGGAAAGAAATGCA |
|  | aphA1 R | CCTATTAATTTCCCCTCGTCAAAAA |
| 37 | aphA3-01 F | AAAAGCCCGAAGAGGAACTTG |
|  | aphA3-01 R | CATCTTTCACAAAGATGTTGCTGTCT |
| 38 | aphA3-02 F | CGGAATTGAAAAAACTGATCGAA |
|  | aphA3-02 R | ATACCGGCTGTCCGTCATTT |
| 39 | bacA-02 F | TTCCACGACACGATTAAGTCATTG |
|  | bacA-02 R | CGGCTCTTTCGGCTTCAG |
| 40 | bla1 F | GCAAGTTGAAGCGAAAGAAAAGA |
|  | bla1 R | TACCAGTATCAATCGCATATACACCTAA |
| 41 | blaACC F | CACACAGCTGATGGCTTATCTAAAA |
|  | blaACC R | AATAAACGCGATGGGTTCCA |
| 42 | blaCMY F | CCGCGGCGAAATTAAGC |
|  | blaCMY R | GCCACTGTTTGCCTGTCAGTT |
| 43 | blaCMY2-01 F | AAAGCCTCAT GGGTGCATAAA |
|  | blaCMY2-01 R | ATAGCTTTTGTTTGCCAGCATCA |
| 44 | blaCMY2-02 F | GCGAGCAGCCTGAAGCA |
|  | blaCMY2-02 R | CGGATGGGCTTGTCCTCTT |
| 45 | blaCTX-M-01 F | GGAGGCGTGACGGCTTTT |
|  | blaCTX-M-01 R | TTCAGTGCGATCCAGACGAA |
| 46 | blaCTX-M-02 F | GCCGCGGTGCTGAAGA |
|  | blaCTX-M-02 R | ATCGGATTATAGTTAACCAGGTCAGATTT |
| 47 | blaCTX-M-05 F | GCGATAACGTGGCGATGAAT |
|  | blaCTX-M-05 R | GTCGAGACGGAACGTTTCGT |
| 48 | blaDHA F | TGGCCGCAGCAGAAAGA |
|  | blaDHA R | CCGTTTTATGCACCCAGGAA |
| 49 | blaGES F | GCAATGTGCTCAACGTTCAAG |
|  | blaGES R | GTGCCTGAGTCAATTCTTTCAAAG |
| 50 | blaIMP-01 F | AACACGGTTTGGTGGTTCTTGTA |
|  | blaIMP-01 R | GCGCTCCACAAACCAATTG |
| 51 | blaIMP-02 F | AAGGCAGCATTTCCTCTCATTTT |
|  | blaIMP-02 R | GGATAGATCGAGAATTAAGCCACTCT |
| 52 | blaIMP-03 F | GGAATAGAGTGGCTTAATTC |
|  | blaIMP-03 R | GGTTTAACAAAACAACCACC |
| 53 | blaKPC-02 F | CAGCTCATTCAAGGGCTTTC |
|  | blaKPC-02 R | GGCGGCGTTATCACTGTATT |
| 54 | blaKPC-03 F | GCCGCCGTGCAATACAGT |
|  | blaKPC-03 R | GCCGCCCAACTCCTTCA |
| 55 | blaL1 F | CACCGGGTTACCAGCTGAAG |
|  | blaL1 R | GCGAAGCTGCGCTTGTAGTC |
| 56 | blaMOX/blaCMY F | CTATGTCAATGTGCCGAAGCA |
|  | blaMOX/blaCMY R | GGCTTGTCCTCTTTCGAATAGC |
| 57 | blaOCH F | GGCGACTTGCGCCGTAT |
|  | blaOCH R | TTTTCTGCTCGGCCATGAG |
| 58 | blaOKP F | GCCGCCATCACCATGAG |
|  | blaOKP R | GGTGACGTTGTCACCGATCTG |
| 59 | blaOXA1/blaOXA30 F | CGGATGGTTTGAAGGGTTTATTAT |
|  | blaOXA1/blaOXA30 R | TCTTGGCTTTTATGCTTGATGTTAA |
| 60 | blaOXA10-01 F | CGCAATTATCGGCCTAGAAACT |
|  | blaOXA10-01 R | TTGGCTTTCCGTCCCATTT |
| 61 | blaOXA58 F | GCAATTGCCTTTTAAACCTGA |
|  | blaOXA58 R | CTGCCTTTTCAACAAAACCC |
| 62 | blaOXY F | CGTTCAGGCGGCAGGTT |
|  | blaOXY R | GCCGCGATATAAGATTTGAGAATT |
| 63 | blaPSE F | TTGTGACCTATTCCCCTGTAATAGAA |
|  | blaPSE R | TGCGAAGCACGCATCATC |
| 64 | blaROB F | GCAAAGGCATGACGATTGC |
|  | blaROB R | CGCGCTGTTGTCGCTAAA |
| 65 | blaSHV-01 F | TCCCATGATGAGCACCTTTAAA |
|  | blaSHV-01 R | TTCGTCACCGGCATCCA |
| 66 | blaSHV-02 F | CTTTCCCATGATGAGCACCTTT |
|  | blaSHV-02 R | TCCTGCTGGCGATAGTGGAT |
| 67 | blaSHV-03 F | GCGTTATTTTCGCCTGTGTA |
|  | blaSHV-03 R | AGGTGCTCATCATGGGAAAG |
| 68 | blaSME F | AACGGCTTCATTTTTGTTTAG |
|  | blaSME R | GCTTCCGCAATAGTTTTATCA |
| 69 | blaTLA F | ACACTTTGCCATTGCTGTTTATGT |
|  | blaTLA R | TGCAAATTTCGGCAATAATCTTT |
| 70 | blaVEB F | CCCGATGCAAAGCGTTATG |
|  | blaVEB R | GAAAGATTCCCTTTATCTATCTCAGACAA |
| 71 | blaVIM F | GCACTTCTCGCGGAGATTG |
|  | blaVIM R | CGACGGTGATGCGTACGTT |
| 72 | blaZ F | GGAGATAAAGTAACAAATCCAGTTAGATATGA |
|  | blaZ R | TGCTTAATTTTCCATTTGCGATAAG |
| 73 | catA1 F | GGGTGAGTTTCACCAGTTTTGATT |
|  | catA1 R | CACCTTGTCGCCTTGCGTATA |
| 74 | catB3 F | GCACTCGATGCCTTCCAAAA |
|  | catB3 R | AGAGCCGATCCAAACGTCAT |
| 75 | cepA F | AGTTGCGCAGAACAGTCCTCTT |
|  | cepA R | TCGTATCTTGCCCGTCGATAAT |
| 76 | cfiA F | GCAGCGTTGCTGGACACA |
|  | cfiA R | GTTCGGGATAAACGTGGTGACT |
| 77 | cfr F | GCAAAATTCAGAGCAAGTTACGAA |
|  | cfr R | AAAATGACTCCCAACCTGCTTTAT |
| 78 | cfxA F | TCATTCCTCGTTCAAGTTTTCAGA |
|  | cfxA R | TGCAGCACCAAGAGGAGATGT |
| 79 | cmeA F | GCAGCAAAGAAGAAGCACCAA |
|  | cmeA R | AGCAGGGTAAGTAAAACTAAGTGGTAAATCT |
| 80 | cmlA1-01 F | TAGGAAGCATCGGAACGTTGAT |
|  | cmlA1-01 R | CAGACCGAGCACGACTGTTG |
| 81 | cmlA1-02 F | AGGAAGCATCGGAACGTTGA |
|  | cmlA1-02 R | ACAGACCGAGCACGACTGTTG |
| 82 | cmlA5 F | GCGCTCTTCGAGGATTCG |
|  | cmlA5 R | CCGCCCAAGCAGAAGTAGAC |
| 83 | cmx(A) F | GCGATCGCCATCCTCTGT |
|  | cmx(A) R | TCGACACGGAGCCTTGGT |
| 84 | dfrA1-01 F | GGAATGGCCCTGATATTCCA |
|  | dfrA1-01 R | AGTCTTGCGTCCAACCAACAG |
| 85 | dfrA1-02 F | TTCAGGTGGTGGGGAGATATAC |
|  | dfrA1-02 R | TTAGAGGCGAAGTCTTGGGTAA |
| 86 | dfrA12 F | CCTCTACCGAACCGTCACACA |
|  | dfrA12 R | GCGACAGCGTTGAAACAACTAC |
| 87 | disul F | TCATCTGCCAAACTCGTCGTTA |
|  | disul R | GTCAAAGAACGCCGCAATGT |
| 88 | emrD F | CTCAGCAGTATGGTGGTAAGCATT |
|  | emrD R | ACCAGGCGCCGAAGAAC |
| 89 | ereA F | CCTGTGGTACGGAGAATTCATGT |
|  | ereA R | ACCGCATTCGCTTTGCTT |
| 90 | ereB F | GCTTTATTTCAGGAGGCGGAAT |
|  | ereB R | TTTTAAATGCCACAGCACAGAATC |
| 91 | ermA-01 F | TTGAGAAGGGATTTGCGAAAAG |
|  | ermA-01 R | ATATCCATCTCCACCATTAATAGTAAACC |
| 92 | ermA-02 F | ACATTTTACCAAGGAACTTGTGGAA |
|  | ermA-02 R | GTGGCATGACATAAACCTTCATCA |
| 93 | ermB F | TAAAGGGCATTTAACGACGAAACT |
|  | ermB R | TTTATACCTCTGTTTGTTAGGGAATTGAA |
| 94 | ermC F | TTTGAAATCGGCTCAGGAAAA |
|  | ermC R | ATGGTCTATTTCAATGGCAGTTACG |
| 95 | ermF F | CAGCTTTGGTTGAACATTTACGAA |
|  | ermF R | AAATTCCTAAAATCACAACCGACAA |
| 96 | ermJ/ermD F | GGACTCGGCAATGGTCAGAA |
|  | ermJ/ermD R | CCCCGAAACGCAATATAATGTT |
| 97 | ermK-01 F | GTTTGATATTGGCATTGTCAGAGAAA |
|  | ermK-01 R | ACCATTGCCGAGTCCACTTT |
| 98 | ermK-02 F | GAGCCGCAAGCCCCTTT |
|  | ermK-02 R | GTGTTTCATTTGACGCGGAGTAA |
| 99 | ermT-01 F | GTTCACTAGCACTATTTTTAATGACAGAAGT |
|  | ermT-01 R | GAAGGGTGTCTTTTTAATACAATTAACGA |
| 100 | ermT-02 F | GTAAAATCCCTAGAGAATACTTTCATCCA |
|  | ermT-02 R | TGAGTGATATTTTTGAAGGGTGTCTT |
| 101 | ermX F | GCTCAGTGGTCCCCATGGT |
|  | ermX R | ATCCCCCCGTCAACGTTT |
| 102 | fabK F | TTTCAGCTCAGCACTTTGGTCAT |
|  | fabK R | AAGGCATCTTTTTCAGCCAGTTC |
| 103 | floR-01 F | ATTGTCTTCACGGTGTCCGTTA |
|  | floR-01 R | CCGCGATGTCGTCGAACT |
| 104 | fosX F | GATTAAGCCATATCACTTTAATTGTGAAAG |
|  | fosX R | TCTCCTTCCATAATGCAAATCCA |
| 105 | gapA F | CCGTTGAAGTGAAAGACGGTC |
|  | gapA R | AACCACTTTCTTCGCACCAGC |
| 106 | imiR F | CCGGACTAGAGCTTCATGTAAGC |
|  | imiR R | CCCACGCGGTACTCTTGTAAA |
| 107 | lmrA-01 F | TTCAGATGCAATGGCGTTTG |
|  | lmrA-01 R | ATAATCGGGAACATAATGAGCATAACTAC |
| 108 | lnuA-01 F | TGACGCTCAACACACTCAAAAA |
|  | lnuA-01 R | TTCATGCTTAAGTTCCATACGTGAA |
| 109 | lnuA-02 F | AGAATGAAAAAGAAGCTGAGCTTCTT |
|  | lnuA-02 R | AAGGTGGCAATTACGTTTTTCAAA |
| 110 | lnuB-01 F | TGAACATAATCCCCTCGTTTAAAGAT |
|  | lnuB-01 R | TAATTGCCCTGTTTCATCGTAAATAA |
| 111 | lnuB-02 F | AAAGGAGAAGGTGACCAATACTCTGA |
|  | lnuB-02 R | GGAGCTACGTCAAACAACCAGTT |
| 112 | lnuC F | TGGTCAATATAACAGATGTAAACCAGATTT |
|  | lnuC R | CACCCCAGCCACCATCAA |
| 113 | marR-01 F | GCGGCGTACTGGTGAAGCTA |
|  | marR-01 R | TGCCCTGGTCGTTGATGA |
| 114 | marR-02 F | TCTGGCGTTAGCTTCACCAGTAC |
|  | marR-02 R | GTGCAAAGGCTGGATCGAA |
| 115 | marR-03 F | GCTGTTGATGACATTGCTCACA |
|  | marR-03 R | CGGCGTACTGGTGAAGCTAAC |
| 116 | mcr-1 F | CGGTCAGTCCGTTTGTTC |
|  | mcr-1 R | CTTGGTCGGTCTGTAGGG |
| 117 | mcr-2 F | TGTTGCTTGTGCCGATTGGA |
|  | mcr-2 R | AGATGGTATTGTTGGTTGCTG |
| 118 | mcr-3 F | TTGGCACTGTATTTTGCATTT |
|  | mcr-3 R | TTAACGAAATTGGCTGGAACA |
| 119 | mcr-4 F | ATTGGGATAGTCGCCTTTTT |
|  | mcr-4 R | TTACAGCCAGAATCATTATCA |
| 120 | mcr-5 F | ATGCGGTTGTCTGCATTTATC |
|  | mcr-5 R | TCATTGTGGTTGTCCTTTTCTG |
| 121 | mdetl1 F | ATACAGCAGTGGATATTGGTTTAATTGT |
|  | mdetl1 R | TGCATAAGGTGAATGTTCCATGA |
| 122 | mdtE/yhiU F | CGTCGGCGCACTCGTT |
|  | mdtE/yhiU R | TCCAGACGTTGTACGGTAACCA |
| 123 | mdtF F | CCACCATCGGGCTTTCC |
|  | mdtF R | CCCTTCTTTCTGCATCATCTCA |
| 124 | mecA F | GGTTACGGACAAGGTGAAATACTGAT |
|  | mecA R | TGTCTTTTAATAAGTGAGGTGCGTTAATA |
| 125 | mefA F | CCGTAGCATTGGAACAGCTTTT |
|  | mefA R | AAACGGAGTATAAGAGTGCTGCAA |
| 126 | mexB F | CTGGAGATCGACGACGAGAAG |
|  | mexB R | GAAATCGTTGACGTAGCTGGAA |
| 127 | mexE F | GGTCAGCACCGACAAGGTCTAC |
|  | mexE R | AGCTCGACGTACTTGAGGAACAC |
| 128 | mexF F | CCGCGAGAAGGCCAAGA |
|  | mexF R | TTGAGTTCGGCGGTGATGA |
| 129 | mphA-01 F | CTGACGCGCTCCGTGTT |
|  | mphA-01 R | GGTGGTGCATGGCGATCT |
| 130 | mphA-02 F | TGATGACCCTGCCATCGA |
|  | mphA-02 R | TTCGCGAGCCCCTCTTC |
| 131 | mphB F | CGCAGCGCTTGATCTTGTAG |
|  | mphB R | TTACTGCATCCATACGCTGCTT |
| 132 | mphC F | CGTTTGAAGTACCGAATTGGAAA |
|  | mphC R | GCTGCGGGTTTGCCTGTA |
| 133 | msrA F | CTGCTAACACAAGTACGATTCCAAAT |
|  | msrA R | TCAAGTAAAGTTGTCTTACCTACACCATT |
| 134 | msrC-01 F | TCAGACCGGATCGGTTGTC |
|  | msrC-01 R | CCTATTTTTTGGAGTCTTCTCTCTAATGTT |
| 135 | msrC-02 F | GAATCACTTGTCCGCAGTTTGTT |
|  | msrC-02 R | CGTACACAACGGTTTCGTCAGA |
| 136 | mtrC-01 F | GGACGGGAAGATGGTCCAA |
|  | mtrC-01 R | CGTAGCGTTCCGGTTCGAT |
| 137 | mtrD-01 F | TGCGCGTAGTCGTTCATCTC |
|  | mtrD-01 R | CGTTCCAATTTCCTGATGATTG |
| 138 | mtrD-02 F | GGTCGGCACGCTCTTGTC |
|  | mtrD-02 R | TGAAGAATTTGCGCACCACTAC |
| 139 | mtrD-03 F | CCGCCAAGCCGATATAGACA |
|  | mtrD-03 R | GGCCGGGTTGCCAAA |
| 140 | ndm F | GGCCACACCAGTGACAATATCA |
|  | ndm R | CAGGCAGCCACCAAAAGC |
| 141 | nimE F | TGCGCCAAGATAGGGCATA |
|  | nimE R | GTCGTGAATTCGGCAGGTTTA |
| 142 | nisB-01 F | GGGAGAGTTGCCGATGTTGTA |
|  | nisB-01 R | AGCCACTCGTTAAAGGGCAAT |
| 143 | nisB-02 F | CGGTTGAACGGCGTGAA |
|  | nisB-02 R | TTTCCACCCAGGTTTGCTACTATT |
| 144 | oleC F | CCCGGAGTCGATGTTCGA |
|  | oleC R | GCCGAAGACGTACACGAACAG |
| 145 | oprD F | ATGAAGTGGAGCGCCATTG |
|  | oprD R | GGCCACGGCGAACTGA |
| 146 | oprJ F | ACGAGAGTGGCGTCGACAA |
|  | oprJ R | AAGGCGATCTCGTTGAGGAA |
| 147 | penA F | AGACGGTAACGTATAACTTTTTGAAAGA |
|  | penA R | GCGTGTAGCCGGCAATG |
| 148 | pikR1 F | TCGACATGCGTGACGAGATT |
|  | pikR1 R | CCGCGAATTAGGCCAGAA |
| 149 | pikR2 F | TCGTGGGCCAGGTGAAGA |
|  | pikR2 R | TTCCCCTTGCCGGTGAA |
| 150 | pmrA F | TTTGCAGGTTTTGTTCCTAATGC |
|  | pmrA R | GCAGAGCCTGATTTCTCCTTTG |
| 151 | pncA F | GCAATCGAGGCGGTGTTC |
|  | pncA R | TTGCCGCAGCCAATTCA |
| 152 | qac-02 F | GGAGATTTAGCTCATGTAGCTGAAGAA |
|  | qac-02 R | AAGCTGTTTTATCCCCGTAGCTTTA |
| 153 | qacA F | TGGCAATAGGAGCTATGGTGTTT |
|  | qacA R | AAGGTAACACTATTTTCGGTCCAAATC |
| 154 | qacE∆1-01 F | TCGCAACATCCGCATTAAAA |
|  | qacE∆1-01 R | ATGGATTTCAGAACCAGAGAAAGAAA |
| 155 | qacE∆1-02 F | CCCCTTCCGCCGTTGT |
|  | qacE∆1-02 R | CGACCAGACTGCATAAGCAACA |
| 156 | qacH-01 F | GTGGCAGCTATCGCTTGGAT |
|  | qacH-01 R | CCAACGAACGCCCACAA |
| 157 | qacH-03 F | GTCGGTGTTGCTTATGCAGTCT |
|  | qacH-03 R | CAACCAGGCAATGGCTGTAA |
| 158 | qnrA F | AGGATTTCTCACGCCAGGATT |
|  | qnrA R | CCGCTTTCAATGAAACTGCAA |
| 159 | qnrB F | GCGACGTTCAGTGGTTCAGA |
|  | qnrB R | GCTGCTCGCCAGTCGAA |
| 160 | rarD-01 F | GCGGGTGTGGTCACTACGAT |
|  | rarD-01 R | AGCGTTGGGCCGATATACTG |
| 161 | rarD-02 F | TGACGCATCGCGTGATCT |
|  | rarD-02 R | AAATTTTCTGTGGCGTCTGAATC |
| 162 | rpoB F | CGAACATCGGTCTGATCAACTC |
|  | rpoB R | GTTGCATGTTCGCACCCAT |
| 163 | sat4 F | GAATGGGCAAAGCATAAAAACTTG |
|  | sat4 R | CCGATTTTGAAACCACAATTATGATA |
| 164 | spcN-01 F | AAAAGTTCGATGAAACACGCCTAT |
|  | spcN-01 R | TCCAGTGGTAGTCCCCGAATC |
| 165 | speA F | GCAAGAGGTATTTGCTCAACAAGA |
|  | speA R | CAGGGTCACCCTCATAAAGAAAA |
| 166 | str F | AATGAGTTTTGGAGTGTCTCAACGTA |
|  | str R | AATCAAAACCCCTATTAAAGCCAAT |
| 167 | strB F | GCTCGGTCGTGAGAACAATCT |
|  | strB R | CAATTTCGGTCGCCTGGTAGT |
| 168 | sul1-01 F | CAGCGCTATGCGCTCAAG |
|  | sul1-01 R | ATCCCGCTGCGCTGAGT |
| 169 | sul1-02 F | TCCGATGGAGGCCGGTATCTGG |
|  | sul1-02 R | CGGGAATGCCATCTGCCTTGAG |
| 170 | sul1-03 F | GCCGATGAGATCAGACGTATTG |
|  | sul1-03 R | CGCATAGCGCTGGGTTTC |
| 171 | tet(32) F | CCATTACTTCGGACAACGGTAGA |
|  | tet(32) R | CAATCTCTGTGAGGGCATTTAACA |
| 172 | tet(35) F | ACCCCATGACGTACCTGTAGAGA |
|  | tet(35) R | CAACCCACACTGGCTACCAGTT |
| 173 | tet(37) F | GAGAACGTTGAAAAGGTGGTGAA |
|  | tet(37) R | AACCAAGCCTGGATCAGTCTCA |
| 174 | tet(38) F | TTAATGTGGCGGTATCTGTAGGTATT |
|  | tet(38) R | TTGCCTGGGAAATTTAATGCTTT |
| 175 | tetA-01 F | GCTGTTTGTTCTGCCGGAAA |
|  | tetA-01 R | GGTTAAGTTCCTTGAACGCAAACT |
| 176 | tetA-02 F | CTCACCAGCCTGACCTCGAT |
|  | tetA-02 R | CACGTTGTTATAGAAGCCGCATAG |
| 177 | tetB-01 F | AGTGCGCTTTGGATGCTGTA |
|  | tetB-01 R | AGCCCCAGTAGCTCCTGTGA |
| 178 | tetB-02 F | GCCCAGTGCTGTTGTTGTCAT |
|  | tetB-02 R | TGAAAGCAAACGGCCTAAATACA |
| 179 | tetC-01 F | CATATCGCAATACATGCGAAAAA |
|  | tetC-01 R | AAAGCCGCGGTAAATAGCAA |
| 180 | tetC-02 F | ACTGGTAAGGTAAACGCCATTGTC |
|  | tetC-02 R | ATGCATAAACCAGCCATTGAGTAAG |
| 181 | tetC-03 F | TGCGTTGATGCAATTTCTATGC |
|  | tetC-03 R | GGAATGGTGCATGCAAGGAG |
| 182 | tetD-01 F | TGCCGCGTTTGATTACACA |
|  | tetD-01 R | CACCAGTGATCCCGGAGATAA |
| 183 | tetD-02 F | TGTCATCGCGCTGGTGATT |
|  | tetD-02 R | CATCCGCTTCCGGGAGAT |
| 184 | tetD-03 F | CTGGACGCGATGGGAATT |
|  | tetD-03 R | TCCGCTTCCGGGAGATATT |
| 185 | tetE F | TTGGCGCTGTATGCAATGAT |
|  | tetE R | CGACGACCTATGCGATCTGA |
| 186 | tetG-01 F | TCAACCATTGCCGATTCGA |
|  | tetG-01 R | TGGCCCGGCAATCATG |
| 187 | tetG-02 F | CATCAGCGCCGGTCTTATG |
|  | tetG-02 R | CCCCATGTAGCCGAACCA |
| 188 | tetH F | TTTGGGTCATCTTACCAGCATTAA |
|  | tetH R | TTGCGCATTATCATCGACAGA |
| 189 | tetJ F | GGGTGCCGCATTAGATTACCT |
|  | tetJ R | TCGTCCAATGTAGAGCATCCATA |
| 190 | tetK F | CAGCAGTCATTGGAAAATTATCTGATTATA |
|  | tetK R | CCTTGTACTAACCTACCAAAAATCAAAATA |
| 191 | tetL-02 F | ATGGTTGTAGTTGCGCGCTATAT |
|  | tetL-02 R | ATCGCTGGACCGACTCCTT |
| 192 | tetM-01 F | CATCATAGACACGCCAGGACATAT |
|  | tetM-01 R | CGCCATCTTTTGCAGAAATCA |
| 193 | tetM-02 F | TAATATTGGAGTTTTAGCTCATGTTGATG |
|  | tetM-02 R | CCTCTCTGACGTTCTAAAAGCGTATTAT |
| 194 | tetM-03 F | GCAATTCTACTGATTTCTGC |
|  | tetM-03 R | CTGTTTGATTACAATTTCCGC |
| 195 | tetO-01 F | ATGTGGATACTACAACGCATGAGATT |
|  | tetO-01 R | TGCCTCCACATGATATTTTTCCT |
| 196 | tetO-02 F | CAACATTAACGGAAAGTTTATTGTATACCA |
|  | tetO-02 R | TTGACGCTCCAAATTCATTGTATC |
| 197 | tetQ F | CGCCTCAGAAGTAAGTTCATACACTAAG |
|  | tetQ R | TCGTTCATGCGGATATTATCAGAAT |
| 198 | tetR-03 F | CGCGATGGAGCAAAAGTACAT |
|  | tetR-03 R | AGTGAAAAACCTTGTTGGCATAAAA |
| 199 | tetS F | TTAAGGACAAACTTTCTGACGACATC |
|  | tetS R | TGTCTCCCATTGTTCTGGTTCA |
| 200 | tetT F | CCATATAGAGGTTCCACCAAATCC |
|  | tetT R | TGACCCTATTGGTAGTGGTTCTATTG |
| 201 | tetU-01 F | GTGGCAAAGCAACGGATTG |
|  | tetU-01 R | TGCGGGCTTGCAAAACTATC |
| 202 | tetU-02 F | AACAGCGGGTTAAGTGTGCAA |
|  | tetU-02 R | ATGGTATCATTCAGTTTTCCGACAAT |
| 203 | tetV F | GCGGGAACGACGATGTATATC |
|  | tetV R | CCGCTATCTCACGACCATGAT |
| 204 | tetW F | ATGAACATTCCCACCGTTATCTTT |
|  | tetW R | ATATCGGCGGAGAGCTTATCC |
| 205 | tetX F | AAATTTGTTACCGACACGGAAGTT |
|  | tetX R | CATAGCTGAAAAAATCCAGGACAGTT |
| 206 | tolC-01 F | GGCCGAGAACCTGATGCA |
|  | tolC-01 R | AGACTTACGCAATTCCGGGTTA |
| 207 | tolC-02 F | CAGGCAGAGAACCTGATGCA |
|  | tolC-02 R | CGCAATTCCGGGTTGCT |
| 208 | tolC-03 F | GCCAGGCAGAGAACCTGATG |
|  | tolC-03 R | CGCAATTCCGGGTTGCT |
| 209 | trfA F | ACGAAGAAATGGTTGTCCTGTTC |
|  | trfA R | CGTCAGCTTGCGGTACTTCTC |
| 210 | ttgA F | ACGCCAATGCCAAACGATT |
|  | ttgA R | GTCACGGCGCAGCTTGA |
| 211 | uidA F | AACCACGCGTCTGTTGACTG |
|  | uidA R | CCCGGTTGCCAGAGGTG |
| 212 | vanA F | AAAAGGCTCTGAAAACGCAGTTAT |
|  | vanA R | CGGCCGTTATCTTGTAAAAACAT |
| 213 | vanB-01 F | TTGTCGGCGAAGTGGATCA |
|  | vanB-01 R | AGCCTTTTTCCGGCTCGTT |
| 214 | vanB-02 F | CCGGTCGAGGAACGAAATC |
|  | vanB-02 R | TCCTCCTGCAAAAAAAGATCAAC |
| 215 | vanC-02 F | CCTGCCACAATCGATCGTT |
|  | vanC-02 R | CGGCTTCATTCGGCTTGATA |
| 216 | vanC1 F | AGGCGATAGCGGGTATTGAA |
|  | vanC1 R | CAATCGTCAATTGCTCATTTCC |
| 217 | vanC2/vanC3 F | TTTGACTGTCGGTGCTTGTGA |
|  | vanC2/vanC3 R | TCAATCGTTTCAGGCAATGG |
| 218 | vanD F | CAGAGGAACATAATGTTTCGATAAAATCT |
|  | vanD R | GCCGGATTTTGTGATTCCAA |
| 219 | vanG F | ATTTGAATTGGCAGGTATACAGGTTA |
|  | vanG R | TGATTTGTCTTTGTCCATACATAATGC |
| 220 | vanHB F | GAGGTTTCCGAGGCGACAA |
|  | vanHB R | CTCTCGGCGGCAGTCGTAT |
| 221 | vanHD F | GTGGCCGATTATACCGTCATG |
|  | vanHD R | CGCAGGTCATTCAGGCAAT |
| 222 | vanRA-01 F | CCCTTACTCCCACCGAGTTTT |
|  | vanRA-01 R | TTCGTCGCCCCATATCTCAT |
| 223 | vanRB F | GCCCTGTCGGATGACGAA |
|  | vanRB R | TTACATAGTCGTCTGCCTCTGCAT |
| 224 | vanRC F | TGCGGGAAAAACTGAACGA |
|  | vanRC R | CCCCCCATACGGTTTTGATTA |
| 225 | vanRC4 F | AGTGCTTTGGCTTATCTCGAAAA |
|  | vanRC4 R | TCCGGCAGCATCACATCTAA |
| 226 | vanRD F | TTATAATGGCAAGGATGCACTAAAGT |
|  | vanRD R | CGTCTACATCCGGAAGCATGA |
| 227 | vanSA F | CGCGTCATGCTTTCAAAATTC |
|  | vanSA R | TCCGCAGAAAGCTCAATTTGTT |
| 228 | vanSC-01 F | ATCAACTGCGGGAGAAAAGTCT |
|  | vanSC-01 R | TCCGCTGTTCCGCTTCTT |
| 229 | vanSE F | TGGCCGAAGAAGCAGGAA |
|  | vanSE R | CAATAATACTCGTCAAAGGAGTTCTCA |
| 230 | vanTC-01 F | CACACGCATTTTTTCCCATCTAG |
|  | vanTC-01 R | CAGCCAACAGATCATCAAAACAA |
| 231 | vanTG F | CGTGTAGCCGTTCCGTTCTT |
|  | vanTG R | CGGCATTACAGGTATATCTGGAAA |
| 232 | vanWB F | CGGACAAAGATACCCCCTATAAAG |
|  | vanWB R | AAATAGTAAATTGCTCATCTGGCACAT |
| 233 | vanWG F | ACATTTTCATTTTGGCAGCTTGTAC |
|  | vanWG R | CCGCCATAAGAGCCTACAATCT |
| 234 | vanXA F | CGCTAAATATGCCACTTGGGATA |
|  | vanXA R | TCAAAAGCGATTCAGCCAACT |
| 235 | vanXB F | AGGCACAAAATCGAAGATGCTT |
|  | vanXB R | GGGTATGGCTCATCAATCAACTT |
| 236 | vanYB F | GGCTAAAGCGGAAGCAGAAA |
|  | vanYB R | GATATCCACAGCAAGACCAAGCT |
| 237 | vanYD-01 F | AAGGCGATACCCTGACTGTCA |
|  | vanYD-01 R | ATTGCCGGACGGAAGCA |
| 238 | vatB-01 F | GGAAAAAGCAACTCCATCTCTTGA |
|  | vatB-01 R | TCCTGGCATAACAGTAACATTCTGA |
| 239 | vatC-01 F | CGGAAATTGGGAACGATGTT |
|  | vatC-01 R | GCAATAATAGCCCCGTTTCCTA |
| 240 | vatD F | TGCAATAGTAGCTGCTAATTCTGTTGTT |
|  | vatD R | TGTTTTATTTCGTTAGCAGGATTTCC |
| 241 | vatE F | GACCGTCCTACCAGGCGTAA |
|  | vatE R | TTGGATTGCCACCGACAATT |
| 242 | vgaA-02 F | GACGGGTATTGTGGAAAGCAA |
|  | vgaA-02 R | TTTCCTGTACCATTAGATCCGATAATT |
| 243 | vgaB-01 F | TAAAAGAGAATAAGGCGCAAGGA |
|  | vgaB-01 R | TGTTTAGTAGCATGTTGCATTTTCC |
| 244 | vgb-02 F | CCACGATGGCTGCCTTTG |
|  | vgb-02 R | GGCCATGCAGGACGGATAT |
| 245 | vgbB-01 F | CAGCCGGATTCTGGTCCTT |
|  | vgbB-01 R | TACGATCTCCATTCAATTGGGTAAA |
| 246 | yceE/mdtG-01 F | TGGCACAAAATATCTGGCAGTT |
|  | yceE/mdtG-01 R | TTGTGTGGCGATAAGAGCATTAG |
| 247 | yceL/mdtH-01 F | TCGGGATGGTGGGCAAT |
|  | yceL/mdtH-01 R | CGATAACCGAGCCGATGTAGA |
| 248 | yyaR F | CCGTTGCAAGAAGATTATAGAAAAAA |
|  | yyaR R | CAAGCATAAGACCGCATAAATGAT |
